# Modular synthesis, host–guest complexation and solvation-controlled relaxation of nanohoops with donor–acceptor structures†

**DOI:** 10.1039/d2sc05804a

**Published:** 2022-11-10

**Authors:** Han Deng, Zilong Guo, Yaxin Wang, Ke Li, Qin Zhou, Chang Ge, Zhanqiang Xu, Sota Sato, Xiaonan Ma, Zhe Sun

**Affiliations:** Department of Chemistry, Institute of Molecular Plus 92 Weijin Road Tianjin 300072 China xiaonanma@tju.edu.cn zhesun@tju.edu.cn; Department of Applied Chemistry, Integrated Molecular Structure Analysis Laboratory, Social Cooperation Program, The University of Tokyo Hongo Bunkyo-ku Tokyo 113-8656 Japan; Haihe Laboratory of Sustainable Chemical Transformations Tianjin 300072 China

## Abstract

Carbon nanohoops with donor–acceptor (D–A) structures are attractive electronic materials and biological fluorophores, but their synthesis is usually challenging. Moreover, the preparation of D–A nanohoop fluorophores exhibiting high fluorescence quantum yields beyond 500 nm remains a key challenge. This study presents a modular synthetic approach based on an efficient metal-free cyclocondensation reaction that readily produced nine congeners with D–A or donor–acceptor–donor′ (D–A–D′) structures, one of which is water-soluble. The tailored molecular design of nanohoops enabled a systematic and detailed study of their host–guest complexation with fullerene, optical properties, and charge transfer (CT) dynamics using X-ray crystallography, fluorescence titration, steady and ultrafast transient absorption spectroscopy, and theoretical calculations. The findings revealed intriguing physical properties associated with D–A motifs, such as tight binding with fullerene, moderate fluorescence quantum yields (37–67%) beyond 540 nm, and unique solvation-controlled CT relaxation of D–A–D′ nanohoops, where two CT states (D–A and A–D′) can be effectively tuned by solvation, resulting in dramatically changed relaxation pathways in different solvents.

## Abstract 2

A modular synthetic approach based on cyclocondensation reaction is introduced to produce nine nanohoops with tunable donor–acceptor structures.

## Graphical abstract

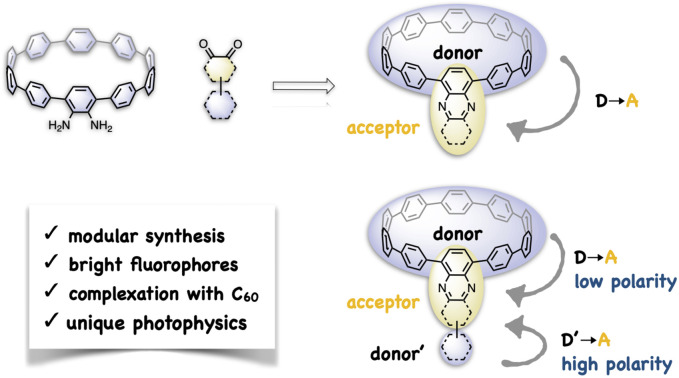

## Introduction

**Fig. 1:**
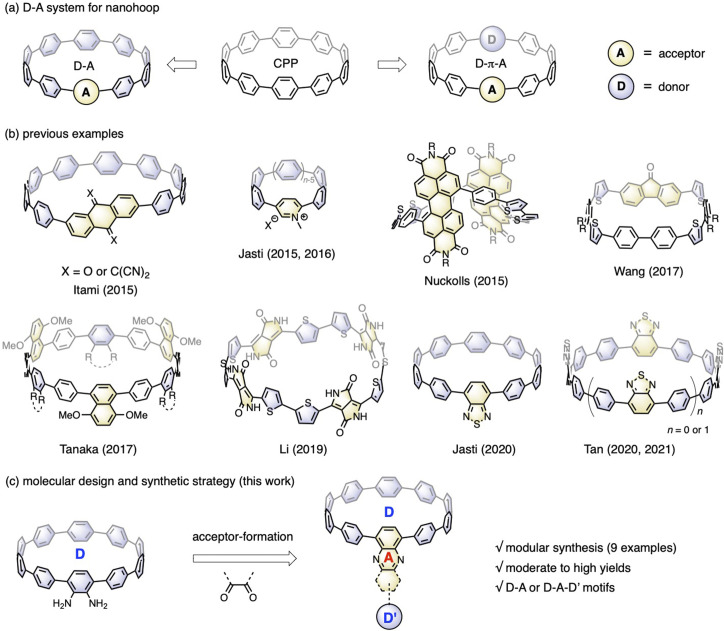
(a) Two types of nanohoops bearing D–A moieties; (b) previously reported examples of D–A nanohoops; (c) molecular design and synthetic strategy of this work for D–A nanohoops.

Incorporating electronic donor (D) and acceptor (A) units into the molecular backbone endows organic materials with attractive features, such as the redistribution of frontier orbitals,^1^ solvatofluorochromism,^2^ and captodative effect.^3^ These features are highly important for material applications in the fields of organic electronics,^4^ photovoltaics^5^ and biology.^6^ In contrast to conjugated polymers with linearly aligned D–A motifs,^7^ the advent of cycloparaphenylene (CPP),^8^ also known as carbon nanohoops, has made it possible to confine the D–A units into a cyclic geometry with quasi-infinite conjugation,^9^ bringing about exciting new features in association with the unique geometric and electronic structures of nanohoops.^10^Fig. 1a shows the two main D–A nanohoops reported so far. The first type has one acceptor in the molecular backbone, while the rest of the CPP subunits are considered donors, because their bent π-scaffold results in higher energy for the highest occupied molecular orbital (HOMO) compared to the linear counterparts. With this design concept, Itami reported two D–A nanohoops with anthraquinone and tetracyanoanthraquinodimethane installed as acceptors.^11^ Jasti reported pyridinium embedded nanohoops with various hoop sizes^12^ and then a D–A nanohoop containing a benzothiadiazole (BT) moiety.^13^ The second type involves aligning D and A units alternately, often with phenylene acting as a spacer. Examples of this genre include molecular systems prepared by Nuckolls,^14^ Wang,^15^ Tanaka,^16^ Li,^17^ and Tan,^18^ in which imide, fluorenone, ester, diketopyrrolopyrrole, and BT were employed as acceptors and thiophenylene and phenylene were introduced as donors (Fig. 1b). Applications in the fields of organic electronics^19^ and bioimaging^20^ have started to emerge, even though there are now just a few examples available.

Nanohoops with D–A motifs are particularly intriguing for bioimaging, considering their low cytotoxicity.^20^ However, applications in biological systems usually require a red emission with a high fluorescence quantum yield (FQY). Unfortunately, most of the abovementioned D–A bearing nanohoops experienced a significant drop in FQY with red-shifted emission, with only a few BT-containing candidates retaining a moderate-to-high FQY with an emission wavelength above 500 nm.^13^ To further meet the application demand, an expanded library of molecules with a well-established structure–property relationship is highly desired. To this end, Jasti *et al.* conducted a systematic theoretical analysis with 18 D–A motifs theoretically examined, thus providing a theoretical blueprint for the molecular design.^21^ To make these motifs synthetically accessible, a modular synthetic approach is desirable.

According to the nanohoop-forming strategies discovered by Jasti/Bertozzi,^8*a*^ Yamago,^22^ Itami,^23^ and Tsuchido/Osakada,^24^ the donor and acceptor units can be introduced before or during the macrocyclization stage. When the donor and acceptor moieties are changed using this synthetic strategy, synthons must be changed from the beginning of the synthetic route, which slows down the rapid expansion of the material scope. In a previous study, we prepared a diamino-[10]CPP derivative *via* a multi-step synthetic sequence and discovered that it could undergo a cyclocondensation reaction efficiently with tetraketone compounds.^25^ The cyclocondensation reaction could transform the formerly electron-donating amino-containing phenylene into an electron-withdrawing moiety. Additionally, stronger electron donors (D′) can be readily introduced by a transition-metal-catalyzed cross-coupling reaction. Therefore, starting from diamino-[10]CPP as a key precursor in this study, a series of D–A or D–A–D′ systems are readily accessible by simply changing the diketone moieties (Fig. 1c). The obtained molecular structures are similar to those of CPPs embedded with polycyclic aromatic hydrocarbons (PAHs) reported by Du,^26^ but with an additional D–A feature. This modular synthetic strategy enabled the accumulation of nine congeners with deliberate structural design, and their electronic structures, host–guest chemistry and ultrafast photophysics were systematically investigated.

## Results and discussion

### Synthesis and electronic structures

**Scheme 1:**
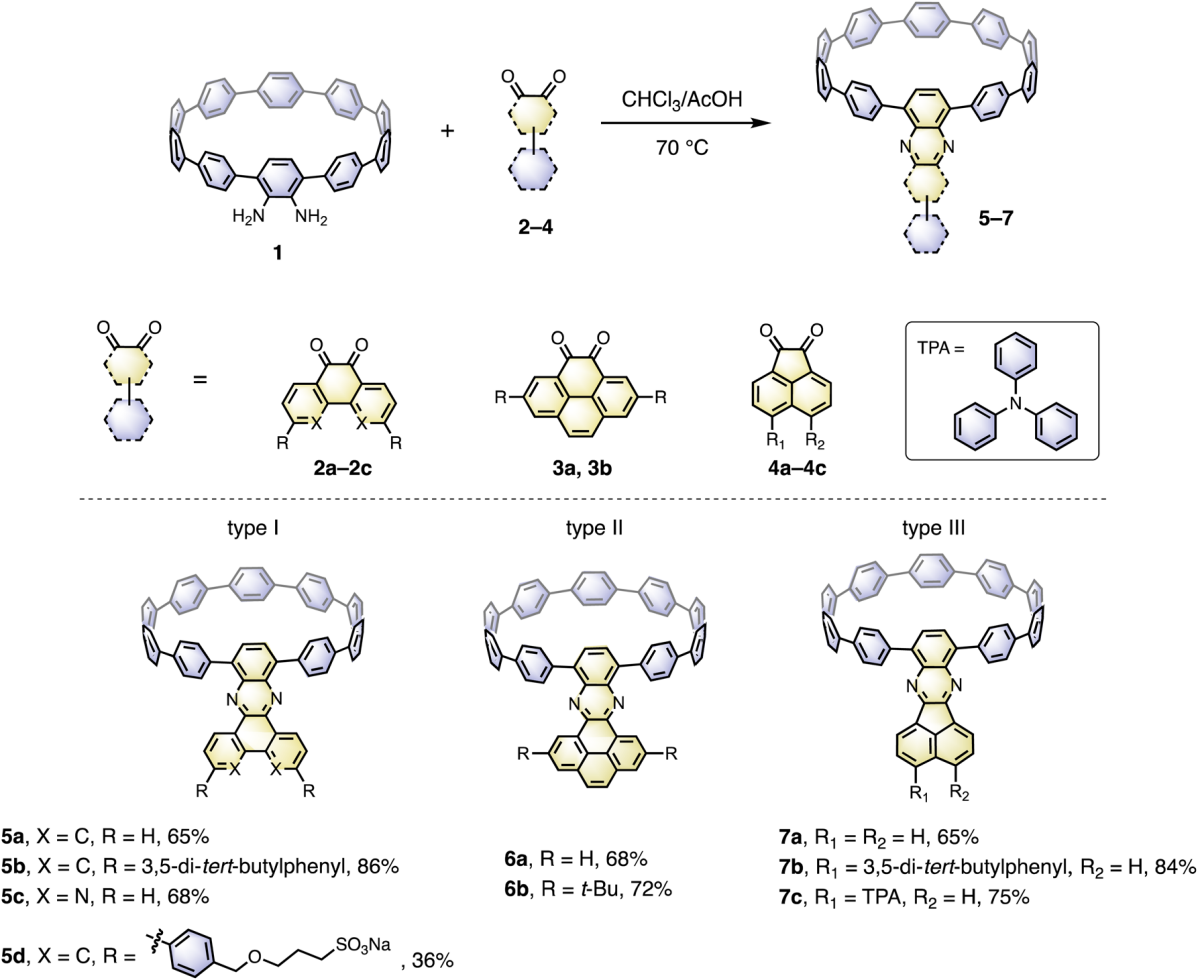
Synthesis of nanohoops with a D–A structure.

The cyclocondensation reaction between diamine and diketone is a robust, metal-free reaction commonly adopted to construct complicated polycyclic aromatic systems.^27^ We discovered that this reaction could be readily applicable to a [10]CPP diamine derivative (1) and various diketones (**2–4**) under standard reaction conditions containing only chloroform and acetic acid as solvents (Scheme 1). The diketones were either commercially available or easily prepared according to the literature procedures.^28^ The reactions proceeded with moderate to good yields, and the workup and purification procedures were simple and handy (see ESI† for details). After the condensation, the formerly electron donating phenylene with a diamino-group was converted into an electron-withdrawing unit, thus forming a D–A nanohoop. With different diketones, phenanthrene- (type I, 5a–5d), pyrene- (type II, 6a, 6b) and acenaphthylene-type (type III, 7a–7c) congeners were prepared. Notably, compound 5c with further nitrogen-doping could also be obtained, representing an attractive motif for metal complexation.^29^ In addition, aliphatic or aromatic substituents can be readily introduced onto the acceptor units, to improve the solubility and to further tune the electronic nature of the nanohoops. Consequently, a water-soluble congener 5d was prepared by introducing two water solubilizing sulfonate substituents (Scheme S2†).^30^ An asymmetric D–A–D′ motif 7c, with vertically aligned CPP as a weaker donor and triphenylamine (TPA) as a stronger donor, was also successfully prepared.

**Fig. 2:**
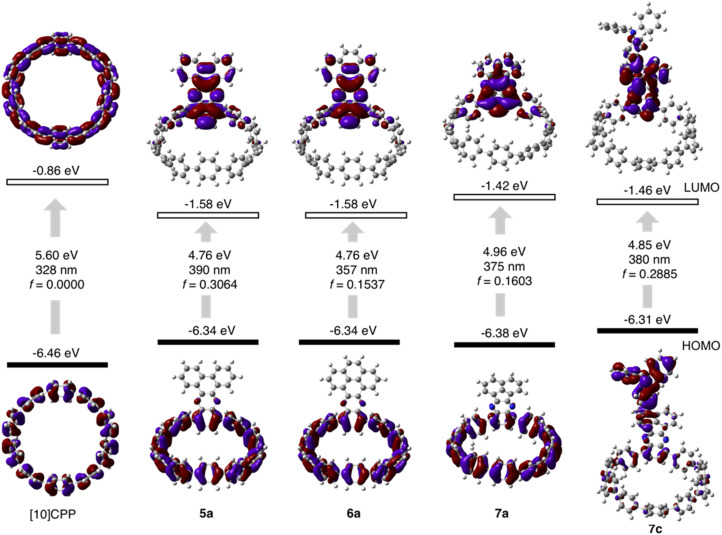
Calculated frontier orbital distributions and energy levels of [10]CPP and D–A nanohoops in this study.

The molecular structures of nanohoops were elucidated using nuclear magnetic resonance (NMR) spectra and high-resolution mass spectra. After extensive trials, we were unable to obtain single crystal structures, but were able to obtain the crystal structure of 6a after its encapsulation with C_60_ (*vide infra*), which supported the formation of the nanohoop structure. To investigate the electronic features resulting from the D–A motifs, density functional theory (DFT) calculations at the M06-2X/6-311G** level were conducted to provide the frontier molecular orbitals of model compounds of each type, 5a, 6a and 7a, together with the D–A–D′ motif 7c (Fig. 2). In comparison to [10]CPP with a fully delocalized HOMO and the lowest unoccupied molecular orbital (LUMO), spatial separation of frontier orbitals was observed for all four molecules, with the HOMO localized on the donor and LUMO distributed on the acceptor. A concomitant reduction of the LUMO and elevation of the HOMO were also found, leading to a narrowed HOMO–LUMO gap. The connection of an additional strong TPA donor in 7c led to elevation of the HOMO and reduction in LUMO levels. The cyclic voltammograms of 5a, 6a, 7a and 7c showed only oxidation waves, and half-wave potentials were in line with the calculated HOMO levels (Fig. S1 and Table S1†). According to the time-dependent DFT (TD-DFT) calculation, the HOMO → LUMO transition, which was Laporte forbidden for [10]CPP,^31^ was partially allowed for the D–A nanohoops in this study because of the broken symmetry in orbitals.

### Host–guest complexation with C_60_

**Fig. 3:**
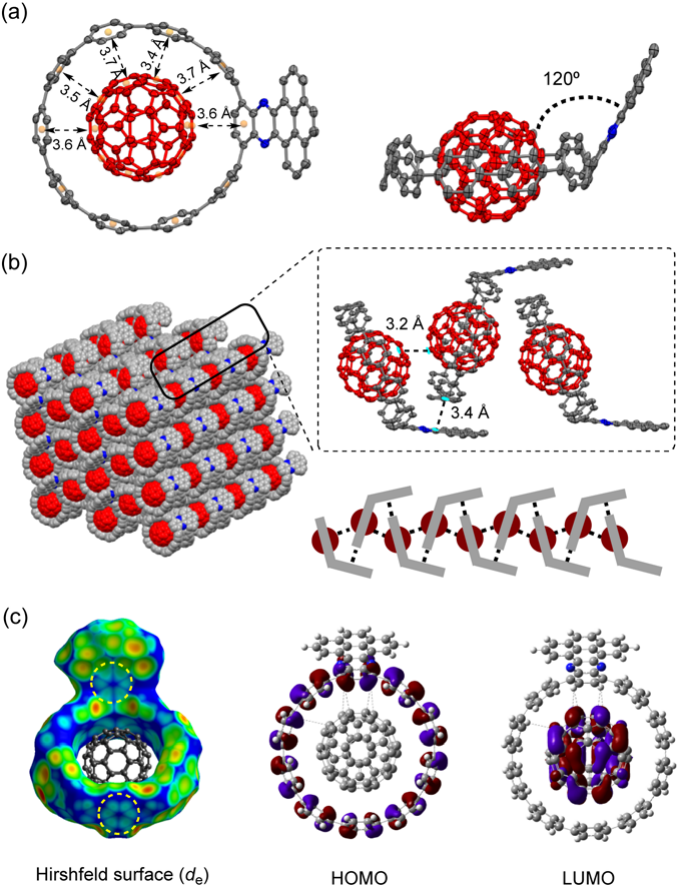
(a) Top view and side view of the X-ray crystallographic structure of 6a⊃C_60_ at 160 K; (b) the packing structure; (c) Hirshfeld surface analysis and frontier molecular orbitals of 6a⊃C_60_.

Recently, several C_60_ hosts have been prepared to realize different functions.^32^ As a well-studied C_60_ host, [10]CPP possessed ideal size complementarity for encapsulating C_60_ with noncovalent van der Waals (vdW) interaction.^33^ Considering that C_60_ is a well-known acceptor itself, it is interesting how nanohoop hosts with D–A features could influence the complexation. We first disclose the complexation in the solid state. The single crystal structure of 6a⊃C_60_ was obtained by slow diffusion of *n*-hexane into a CHCl_3_ solution of 6a and C_60_ at −20 °C. Note that the single crystal of the complex was easier to achieve than the free-standing 6a, suggesting that C_60_ serves as a template for the crystal growth.^34^ As shown in Fig. 3a, a 1 : 1 host–guest complex formed with the C_60_ sited in the center of the nanohoop. The intermolecular distances of the host and the guest were in the range of 3.4–3.7 Å, indicating a dominant vdW interaction. The C_60_ molecules were separated by a distance of 3.2 Å, and the continuous vdW contact between the C_60_ molecules suggested the possibility of a conductive pathway for electrons, which is potentially useful for electronic applications. Although the incorporation of the π-extended acceptor had an insignificant influence on the circular shape of the nanohoop (Fig. S2†), the torsional angles of the acceptor and the adjacent phenyl (52° and 54°) were larger than the rest of the CPP units (45° on average, Table S3†). This resulted in a tilting dihedral angle of 120° between the acceptor unit and the CPP mean plane. In the packing structure, each molecule was zipped together by the π–π interaction between neighbouring C_60_ and between the acceptor unit and phenyl (Fig. 3b), to form a one-dimensional stacking. Such interactions sewing the complexes together can be further visualized by *d*_e_ mapping from the Hirshfeld analysis,^35^ which demonstrated a similar pattern of close contact in the interior of the acceptor and exterior of the phenyl ring (Fig. 3c). The solvent molecules of *n*-hexane and CHCl_3_ were filled in the curved space between the two complexes (Fig. S4†). The frontier orbitals of the complex were calculated using DFT at the LC-BLYP/6-311G* level^36^ with basis set superposition error (BSSE) correction.^37^ The HOMO was mainly localized on the host and the LUMO on the guest, similar to the case of [10]CPP, whereas the LUMO+3 orbital was distributed on the acceptor unit (Fig. S19†). The calculation suggested that the charge transfer between the host and guest dominated in the complex.

The binding behavior was then investigated in the solution phase with four nanohoops, 5a, 6a, 7a and 7c, as model compounds. Upon the addition of C_60_ into the *o*-DCB solution of hosts, instant fluorescence quenching was observed, in agreement with the charge transfer nature of the complex (Fig. S19†). The binding stoichiometry in solution was estimated to be 1 : 1, based on the titration experiments at different concentrations that are all well fitted to the 1 : 1 model (Fig. S9 and Table S4†).^38^ The ^1^H NMR spectrum of 6b⊃C_60_ measured in CDCl_3_ at −30 °C shows a significant change in chemical shifts of the phenylene protons compared to the free-standing 6b, whereas the protons on nitrogen-containing PAH were less affected (Fig. S10†). As presented in Table 1, triplicate fluorescence quenching titration in *o*-dichlorobenzene (*o*-DCB) gave similar binding constants for all four complexes, in the order of 10^5^ to 10^6^ M^−1^, which was two to three orders of magnitude higher than that of [10]CPP⊃C_60_ measured in the same solvent (Fig. S5–S8†).^33*a*^ The enhancement in binding could be due to (1) an enlargement of the π–π interaction area^26*a*^ and (2) an elevation in the HOMO level of the D–A nanohoop which facilitated the strong electrostatic interactions between the host and guest.

**Table 1:** Binding constants and Gibbs energies of complexes measured in *o*-DCB

	*K*_a_ (M^−1^)	Δ*G* at 298 K (kcal mol^−1^)
5a⊃C_60_	(9.10 ± 0.12) × 10^5^	−8.10 ± 0.01
6a⊃C_60_	(1.05 ± 0.21) × 10^6^	−8.18 ± 0.02
7a⊃C_60_	(7.69 ± 0.12) × 10^5^	−7.79 ± 0.01
7c⊃C_60_	(7.61 ± 0.60) × 10^5^	−7.78 ± 0.04
[10]CPP⊃C_60_ (ref. 33a)	(6.02 ± 0.18) × 10^3^	−5.15 ± 0.02

### Optical and excited state properties

**Fig. 4:**
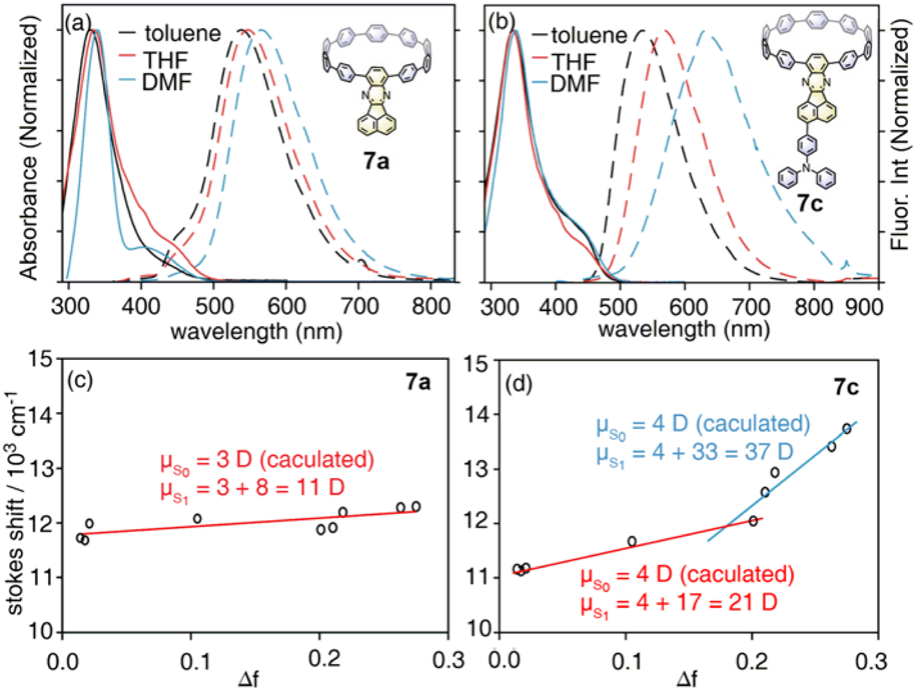
Absorption (solid lines) and fluorescence spectra (dashed lines) of (a) 7a and (b) 7c in selected solvents, and the corresponding Lippert–Mataga fittings of (c) 7a and (d) 7c.

The absorption and emission spectra of 9 synthesized D–A nanohoops were measured in CH_2_Cl_2_ and water solutions (Fig. 4, S14 and S16†) and the data are summarized in Table 2. All compounds showed maximum absorptions around 330 nm, which is typical of the CPP structure. The shoulder peaks in the region of 400–500 nm are from the partly allowed HOMO → LUMO transition (Fig. 2). Compared to the emission of the parent [10]CPP (466 nm), the emissions were substantially red-shifted to a region of 540–610 nm, falling in the optical window desirable for biological applications. Gratifyingly, a moderate FQY was retained at a level of 37–67%, in contrast to other nitrogen containing D–A CPPs.^12^ This could be due to the absence of a nonradiative relaxation channel in our system. Note that 5d exhibits a FQY of 16% in an aqueous solution, which is comparable to Jasti's water soluble nanohoop but with a 69 nm redshift in emission (510 nm *vs.* 579 nm).^30*a*^ The plot of wavenumber against the solvent parameter *E*_T_(30)^39^ revealed positive solvatofluorochromism due to intramolecular charge transfer (Fig. S15†).

**Table 2:** Optical properties of [10]CPP and D–A nanohoops in this study

	*λ*_abs_/nm	*λ*_em_/nm	FQY (%)	Solvent
5a	334, 448, 453	590	67	CH_2_Cl_2_
5b	333, 418	590	53	CH_2_Cl_2_
5c	331	602	43	CH_2_Cl_2_
5d	337, 394, 417	593	56	CH_2_Cl_2_
5d	338, 392, 429	579	16	H_2_O
6a	333, 423, 447	593	60	CH_2_Cl_2_
6b	322, 428, 449	593	52	CH_2_Cl_2_
7a	329	555	40	CH_2_Cl_2_
7b	333	542	37	CH_2_Cl_2_
7c	332	579	65	CH_2_Cl_2_
[10]CPP^8*c*^	338	466	65	CH_2_Cl_2_

With model systems 7a and 7c, the photophysics of D–A and D–A–D′ nanohoops were further investigated using steady and ultrafast spectroscopic measurements. As shown in Fig. 4, the absorption spectra of 7a and 7c were dominated by a pronounced peak at approximately 330 nm, corresponding to a local excited state of CPP rings (LE_CPP_) as revealed by natural transition orbital analysis (NTO, see Fig. S20†),^40^*i.e.* S_0_ → S_3_ (7a, 3.590 eV) and S_0_ → S_4_ states (7c, 3.585 eV) with pronounced oscillator strength. As forbidden transitions, the local excited states of the acceptor (LE_A_) were also revealed through calculations for 7a (S_2_) and 7c (S_3_) with low oscillator strength. Meanwhile, weak absorption in the 400–500 nm regime was observed for 7a and 7c as a shoulder of the LE_CPP_ peak. For 7a (D–A), the NTO analysis (Fig. S20a†) indicated pronounced charge transfer (CT_CPP→A_) characteristics of the lowest-lying S_1_ state (∼3.3 eV) below the LE_A_ (S_2_) state. However, the case of 7c (D–A–D′) was more complicated, *i.e.* both S_1_ and S_2_ states exhibited a CT character but corresponded to different donors. Under weak solvation (calculated using PCM = toluene), the S_0_ → S_1_ transition of 7c corresponds to CT_CPP→A_ whereas TPA acts as a donor (CT_A←TPA_) for S_0_ → S_2_ with an even higher oscillator strength (Fig. S20b†). Two plausible CT states (CT_CPP→A_ and CT_A←TPA_) might be involved in the relaxation of 7c due to its unique asymmetric D–A–D′ structure with weak and intense donors, *i.e.* CPP (D) and TPA (D′), respectively.

To verify the assignment, the quantitative CT/LE contribution of each excited state was estimated using hole–electron analysis of 7a and 7c (see Table S11†)^41,42^ Regarding D–A nanohoop 7a, the LE_CPP_ state (S_3_) was confirmed with approximately 85% hole–electron overlapping and LE% up to approximately 94%. However, a weak (∼24%) CT character was observed for the S_1_ state (CT_CPP→A_). Note that the calculated LE/CT contribution is highly dependent on the pre-setting of D/A fragments.^41,43^ The CPP was set as a donor for 7a, but more options must be considered for asymmetric D–A–D′ (7c). Without loss of generality, we calculated CT% of each excited state of 7c with three pre-settings (Table S11†): (1) TPA as a donor, the S_2_ state exhibits an ∼31% CT character; (2) CPP as a donor, the S_1_ state exhibits approximately a 19% CT character; (3) TPA and CPP as donors, both S_2_ and S_1_ exhibit a CT character of approximately 37% and 24%, respectively. The results confirmed that both S_1_ and S_2_ of 7c were CT states but with different donors (*i.e.* CT_CPP→A_ and CT_A←TPA_, respectively). The higher CT% and oscillator strength of CT_A←TPA_ indicate that TPA is stronger than CPP for donating electrons. According to the results, the existence of TPA might lead to dramatically changed photophysics of 7c. The LE_CPP_ (S_4_) and LE_A_ (S_3_) states were also confirmed, and the assignment of S_1_ to S_4_ states of 7a and 7c is summarized in Table 3.

**Table 3:** The TD-DFT (M06-2X/6-311G**, PCM = toluene) calculated excitation energy, oscillator strength and tentative assignment for low-lying excited states of 7a and 7c

States	**7a** (CPP-A)			**7c** (CPP-A-TPA)		
	Excitation energy (eV)	Oscillator strength	Assignment	Excitation energy (eV)	Oscillator strength	Assignment
S_1_	3.3005	0.1603	CT_CPP→A_	3.2590	0.2885	CT_CPP→A_
S_2_	3.4710	0.0080	LE_A_	3.3421	1.0278	CT_A←TPA_
S_3_	3.5901	1.0510	LE_CPP_	3.4929	0.0021	LE_A_
S_4_	3.6597	0.2025	CT_CPP→A_	3.5852	0.7621	LE_CPP_

Due to the dipolar character, the energy level of CT states can be effectively tuned by the solvation effect,^44^*i.e.* solvatochromism. As CT_A←TPA_ is considered to be more dipolar than CT_CPP→A_, more pronounced solvatochromism can be expected for CT_A←TPA_, which was further verified (Fig. 4, bottom panel). As shown in Fig. 4, 7a exhibited a linear dependence of the Stokes shift on solvent polarity (Δ*f*), whereas the Lippert–Mataga fitting^45^ resulted in approximately 10.95 debye dipole moment of the S_1_ state (*μ*_S1_, for details see ESI, Section 5.2†). In contrast, segmented dependence of the Stokes shift was observed with Δ*f* for D–A–D′ 7c. In the weak solvation regime (low polarity solvents, Δ*f* < 0.2), *μ*_S1_ was estimated to be approximately 20.98 debye. However, a different S_1_ state of 7c with almost doubled *μ*_S1_ (∼37.10 debye) was observed in a strong solvation regime (Δ*f* > 0.2), which was further confirmed using fluorescence lifetime measurements. As shown in Fig. S18,† the fluorescence decay (S_1_ lifetime) of 7a was similar in toluene, tetrahydrofuran (THF) and dimethylformamide (DMF), corresponding to the emission of an identical S_1_ state, *i.e.* CT_CPP→A_.^46^ However, the fluorescence lifetime of 7c in DMF was significantly shorter than that in toluene and THF, indicating a different S_1_ state of 7c under weak and strong solvation conditions. Compared with the CT_CPP→A_ (S_1_) state in low polarity solvents, we believe that the CT_A←TPA_ state of 7c was energetically lowered down due to strong solvation in high polarity solvents, *e.g.* DMF, while solvatochromism of the CT_CPP→A_ state was less pronounced due to a lower dipole moment. Consequently, CT_A←TPA_ becomes the lowest-lying S_1_ state of 7c in DMF, whereas CT_CPP→A_ serves as a S_1_ state in toluene and THF, which may lead to dramatically changed ultrafast relaxation dynamics of 7c in solvents of different polarity.

## Ultrafast charge transfer dynamics

**Fig. 5:**
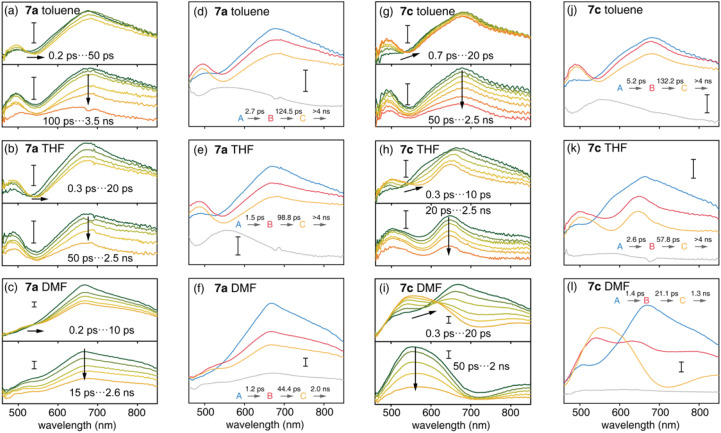
Transient absorption spectra (a–c and g–i) and species-associated spectra (SAS) (d–f and j–l) of 7a and 7c in toluene (upper panel), THF (medium panel) and DMF (lower panel). Time constants were obtained from target analysis with a four step sequential model. The scale bar of signal intensity represents 1 mOD.

The S_1_ state plays an important role in the photophysics of organic chromophores.^47^ Compared with the D–A nanohoop (7a), the coexistence of CT_CPP→A_ and CT_A←TPA_ states may provide the D–A–D′ nanohoop (7c) with unique electronic relaxation where the energy levels of two CT states might be effectively tuned by solvation. This was further characterized by using femtosecond transient absorption (fs-TA) spectroscopy. Upon excitation at 330 nm, fs-TA spectra of 7a and 7c in toluene, THF and DMF solutions were measured in the 460–850 nm probe range with approximately 200 fs temporal resolution (Fig. 5).

As shown in Fig. 5(a–c), D–A nanohoops (7a) exhibited a similar fs-TA signal in toluene, THF and DMF. According to the TD-DFT calculation (Table 3), the LE_CPP_ state of 7a is initially populated by 330 nm excitation, corresponding to a broad feature (550–850 nm, maximum at ∼690 nm) which was consistent with the reported excited state absorption (ESA) band of [10]CPP.^48^ The decay of the LE_CPP_ signal was accompanied by the rise of a new band at ∼490 nm, corresponding to an ultrafast charge transfer process (LE_CPP_ → CT_CPP→A_).^49^

Meanwhile, spectral depletion at approximately 525 nm was observed in the initial TA spectra and red-shifts were observed in subsequent delay steps up to several picoseconds, which was attributed to dynamic Stokes shifting of the stimulated emission (SE) band, *i.e.* the solvation of the CT_CPP→A_ state. Note that the solvation of CT states is typically ultrafast in simple organic dipolar solvents,^50^*i.e.* down to the sub-picosecond time scale. Therefore, the SE dynamic shifting might not be fully disentangled from the LE_CPP_ → CT_CPP→A_ process due to the limitation of temporal resolution. The subsequent relaxation of solvated CT_CPP→A_ was further observed in several nanoseconds.

The D–A–D′ nanohoop (7c) in toluene exhibited nearly identical fs-TA spectra of 7a (Fig. 5g) as expected, because CT_CPP→A_ still serves as the lowest-lying state for 7c under weak solvation conditions. In THF, the ESA band (CT_CPP→A_) was observed to be slightly extended to a longer wavelength (Fig. 5h). Furthermore, 7c showed different TA responses under strong solvation conditions in DMF (Fig. 5i). The dynamic shifting of the SE band (CT_CPP→A_) eventually leads to a pronounced ESA band in the 500–650 nm regime, which is similar to the reported ESA band of the solvated CT state with a TPA donor of a symmetric D–A–D chromophore (Fig. S21†).^51^ Furthermore, as discussed above, the energy level of CT_CPP→A_ and CT_A←TPA_ of 7c can be effectively tuned by solvation. In DMF, the energy level of solvated CT_A←TPA_ becomes even lower than CT_CPP→A_ due to stronger solvation. As a result, relaxation of CT_CPP→A_ → CT_A←TPA_ was observed. Therefore, we assigned the formed broad ESA band of 7c in DMF to the solvated CT_A←TPA_ state, which is unobservable under weak solvation conditions.

We further conducted a quantitative target analysis on all TA data, which can be well reproduced using a sequential model containing four independent species (A → B → C → D), and the estimated time constants are summarized in Table 4. The subtracted species-associated spectra (SAS) are illustrated in Fig. 5, while the concentration evolution of each species and time trace at the selected probe wavelength can be seen in Fig. S22 and S23† respectively.

**Table 4:** Target analysis estimated time constants and their assignments

		A → B, *τ*_1_ (ps)	Assignment	B → C, *τ*_2_ (ps)	Assignment	C→, *τ*_3_ (ns)	Assignment
7a	Toluene	2.7	LE_CPP_ → CT_CPP→A_ and solvation of CT_CPP→A_	124	Fast structural relaxation of CT_CPP→A_	>4	Slow relaxation of CT_CPP→A_
	THF	1.5		98.8		>4	
	DMF	1.2		44.4		2.0	
7c	Toluene	5.2	LE_CPP_ → CT_CPP→A_ and solvation of CT_CPP→A_	132	Fast structural relaxation of CT_CPP→A_	>4	Slow relaxation of CT_CPP→A_
	THF	2.6		57.8		>4	
	DMF	1.4		21.1	CT_CPP→A_ → CT_A←TPA_	1.3	Relaxation of CT_A←TPA_

**Fig. 6:**
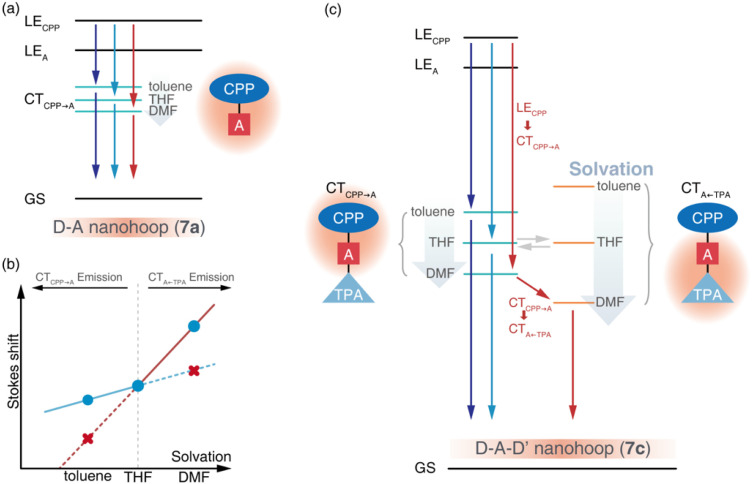
Energy level diagrams illustrating the excited-state dynamics of (a) 7a and (b and c) 7c in solvents of different polarity. The arrows with a solid line represent transition between states, while the arrows as filled bands represent energetic level lowering of CT states due to solvation.

The initial process (A → B) of 7a exhibited ultrafast time constants (*τ*_1_ < 3 ps) and became shorter with increasing solvent polarity, which was consistent with the reported formation and solvation of the CT state.^51,52^ The increased CT_CPP→A_ band at 490 nm and red-shifted SE depletion on SAS of species B further confirmed our assignment of process A → B, *i.e.* LE_CPP_ → CT_CPP→A_ and solvation of CT_CPP→A_. The subsequent process (B → C, *τ*_2_) for tens of picoseconds with an unchanged TA shape was assigned to the fast relaxation of CT_CPP→A_, in which structural relaxation might play a key role as widely reported for organic fluorescent chromophores.^51,52^ The slow relaxation of CT_CPP→A_ (C→, *τ*_3_) leaves a structureless TA signal within nanoseconds, which might include several slow relaxation channels such as intersystem crossing, fluorescent decay or photochemical reactions. The relaxation mechanism of 7a is summarized in Fig. 6a.

The target analysis on TA data of D–A–D′ nanohoops (7c) in toluene led to comparable results of 7a. As shown in Fig. 6c, the solvated CT_CPP→A_ is energetically lower than CT_A←TPA_ under weak solvation, *i.e.* in toluene. Consequently, solvated CT_CPP→A_ formed within *τ*_1_ = 5.2 ps and subsequently relaxed bi-exponentially (*τ*_2_ = 132 ps and *τ*_3_ > 4 ns) without involving CT_A←TPA_. In THF, the energy levels of solvated CT_CPP→A_ and CT_A←TPA_ might be nearly degenerate due to stronger solvation than in toluene. Therefore, a photostationary state including CT_CPP→A_ and CT_A←TPA_ might form within the observable *τ*_1_ = 2.6 ps, which was observed as the spectrally extended SAS of the TA response (species B and C, Fig. 5k) and further decayed bi-exponentially (*τ*_2_ = 57.8 ps and *τ*_3_ > 4 ns). Finally, the strong solvation in DMF changed the relative energy levels between CT_CPP→A_ and CT_A←TPA_. As shown in the Lippert–Mataga model of 7c (Fig. 6b), the energy level of solvated CT_A←TPA_ was below CT_CPP→A_ in DMF. As a result, the formed (*τ*_1_ = 1.4 ps) solvated CT_CPP→A_ subsequently relaxed to CT_A←TPA_ with a time constant of *τ*_2_ = 21.1 ps, corresponding to the observed broad ESA band which dominated the SAS of species C. The formed CT_A←TPA_ further decayed, with a faster decay rate (*τ*_3_ = 1.3 ns) than CT_CPP→A_ (*τ*_3_ = 2.0 ns), indicating their different states of origin.

The relaxation of the solvated CT state has been widely investigated in dipolar solvents.^53^ Meanwhile, symmetric D–A–D and A–D–A chromophores have attracted attention due to the unique excited-state symmetry breaking relaxation,^54^*i.e.* the initially populated quadrupolar state decays to a dipolar state under strong solvation conditions. With model systems 7a and 7c, we demonstrated a unique photophysics mechanism of an asymmetric D–A–D′ chromophore, in which the relative energy levels of two CT states (D–A and A–D′) are highly dependent on solvation. As a result, the relaxation pathway of the D–A–D′ chromophore can be effectively controlled using different solvation conditions.

## Conclusions

In summary, this study described a versatile “late stage” synthetic approach that uses an effective metal-free cyclocondensation reaction, which readily produces nanohoops with D–A or D–A–D′ structures with tailored molecular design in moderate to high yields. Nine congeners, which belong to three structural genres, were synthesized and systematically investigated in terms of host–guest chemistry, optical properties and ultrafast charge transfer dynamics. Importantly, all compounds retain a moderate FQY (37–67%) with emission wavelengths ranging from 540–610 nm. Particularly, one of the nanohoops is water soluble with a FQY up to 16% at 579 nm in the aqueous solution, which is a feature desirable for biological applications. The solvation-controlled charge transfer relaxation of D–A–D′ nanohoops was demonstrated using ultrafast spectroscopic measurements and TD-DFT calculations. The energy level of two CT states (D–A and A–D′) can be effectively tuned by solvation of D–A–D′ nanohoops, which leads to dramatically changed relaxation pathways in different solvents. We envision that the synthetic strategy presented in this study will rapidly expand the scope of nanohoop fluorophores with D–A structures, paving the way for practical applications particularly in the biological field.

## Data availability

The ESI† contains a detailed description of the synthetic method, computational method and the supplementary spectroscopic and crystallographic data.

## Author contributions

Z. S. and X. M. supervised the project. H. D., K. L., Q. Z., C. G. and Z. X. performed synthetic experiments and the study of host–guest chemistry. Z. G. and Y. W. performed spectral measurements and theoretical calculations. S. S. performed crystallographic analysis. All authors analyzed the data, discussed the results, and contributed to the manuscript writing.

## Conflicts of interest

There are no conflicts to declare.

## Supplementary Material

SC-013-D2SC05804A-s001

SC-013-D2SC05804A-s002
