# Supplementary material for: Modular synthesis, host–guest complexation and solvation-controlled relaxation of nanohoops with donor–acceptor structures
Source: Chem Sci. 2022 Nov 10;13(47):14080–9. doi: 10.1039/d2sc05804a (PMC9728570; doi:10.1039/d2sc05804a)
Supplement: SC-013-D2SC05804A-s001 [file SC-013-D2SC05804A-s001.pdf]

Electronic Supplementary Material (ESI) for Chemical Science.
This journal is © The Royal Society of Chemistry 2022

Supporting information

Modular Synthesis, Host-Guest Complexation and Solvation-Controlled Relaxation of Nanohoops with Donor-Acceptor Structures

Han Deng,[†a] Zilong Guo,[†a] Yaxin Wang,[a] Ke Li,[a] Qin Zhou,[a] Chang Ge,[a] Zhanqiang Xu,[a] Sota Sato,[b] Xiaonan Ma,*[a] and Zhe Sun*[ac]

[a]Institute of Molecular Plus, Department of Chemistry, 92 Weijin Road, Tianjin 300072, China

E-mail: xiaonanma@tju.edu.cn, zhesun@tju.edu.cn

[b]Department of Applied Chemistry, Integrated Molecular Structure Analysis Laboratory, Social Cooperation Program, The University of Tokyo, Hongo, Bunkyo-ku, Tokyo 113-8656 Japan.

[c]Haihe Laboratory of Sustainable Chemical Transformations. Tianjin 300072, China.

[†]These authors contributed equally

Table of contents

1. Synthetic methods S3
2. Electrochemical Analysis S12
3. X-ray crystallographic data S12
4. Complexation with C_{60} S16
5. Additional spectroscopic data S19
6. Theoretical calculations S25
7. Ultrafast transient absorption measurements S32
8. $^{1}H/^{13}C$ NMR and MS spectra for new compounds S35
9. References S51

1. Synthetic methods

1.1 General information

All reagents were purchased from the commercial source and used without further purification. Compound **1**[1], 3,6-Bis(3,5-di-*tert*-butylphenyl)phenanthrene-9,10-dione **(2b)**[2], **3b**[3], **4c**[4], **8**[5] were prepared according to reported method. Anhydrous tetrahydrofuran (THF) and toluene was distilled with sodium. Dichloromethane (DCM) was dried over CaH_2 and distilled prior to use. All experiments were performed under argon atmosphere. Column chromatography was performed with silica gel (200-300 mesh). Proton (^1H) NMR and carbon (^{13}C) NMR spectra were recorded on Bruker Ascend 600 NMR spectrometer with tetramethylsilane (TMS) as the internal standard. The abbreviations of s, d, t and dd represent the multiplicities of singlet, doublet, triplet and double-doublet, respectively. Chemical shifts were given in ppm relative to residue protons ($CDCl_3$: δ 7.26 for ^1H, δ 77.16 for ^{13}C; 1,1,2,2-$C_2D_2Cl_4$: δ 6.00 for ^1H, δ 73.78 for ^{13}C). High resolution Electrospray Ionization (ESI) mass spectra were performed on Bruker Micro TOF-Q II instrument. Matrix Assisted Laser Desorption Ionization (MALDI) mass spectra were performed on Bruker Daltonics Autoflex TOF equipment. High resolution Atmospheric Pressure Chemical Ionization (APCI) mass spectra were recorded by ESI on a quadrupole-orbitrap mass spectrometer (QExactive, ThermoFisher Scientific).

The electrochemical measurements were carried out in anhydrous DCM containing *n*-Bu_4NPF_6 as supporting electrolyte under argon atmosphere by CHI 620E electrochemical analyzer. A three-electrode system with glassy carbon as working electrode, Ag/$AgNO_3$ as reference electrode, Pt wire as counter electrode was applied. The potential was calibrated against ferrocene/ferrocenium couple.

The steady UV/vis absorption and fluorescence spectra were recorded on SHIMADZU UV-2600 and FL-1000 spectrophotometer, respectively. The fluorescence quantum yields were determined in absolute values with integrating sphere. The diffraction data

were collected on XtaLAB Synergy diffractometer equipped with Hypix6000HE detector using a single-wavelength X-ray source.

1.2 Synthesis

Synthesis of 5-(3,5-di-*tert*-butylphenyl)acenaphthylene-1,2-dione (4b).

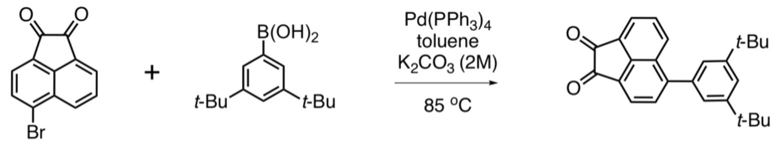

4b

5-Bromoacenaphthylene-1,2-dione (50 mg, 0.19 mmol, 1.0 eq.) and 3,5-di-*tert*-butylphenylboronic acid (68 mg, 0.29 mmol, 1.5 eq.), $Pd(PPh_3)_4$ (13 mg, 0.01 mmol, 0.03 eq.), toluene (2 mL) and K_2CO_3 (2 M in deionized water, 1.5 mL) were added to a Schlenk flask under argon atmosphere. The reaction mixture was stirred overnight at 85 °C. Subsequently, the reaction was quenched with water and the crude mixture was extracted with DCM. The combined organic phase was washed 3 times with water and dried over $MgSO_4$. Then, the solvent was removed under reduced pressure and the obtained crude mixture was purified by column chromatography (silica gel, petroleum ether/DCM = 1/1) to afford yellow fluffy crystals (34 mg, 49 %). ^{1}H NMR (600 MHz, $CDCl_3$) δ = 8.40 (d, J = 8.5 Hz, 1H), 8.19 (d, J = 7.2 Hz, 1H), 8.13 (d, J = 6.8 Hz, 1H), 7.87 – 7.76 (m, 2H), 7.60 (s, 1H), 7.43 (s, 2H), 1.41 (s, 18H) ppm. ^{13}C NMR (150 MHz, $CDCl_3$) δ = 188.92, 188.01, 151.67, 147.81, 146.94, 137.48, 131.98, 129.77, 129.13, 128.95, 128.52, 127.68, 123.93, 123.03, 122.34, 122.10, 35.22, 31.64 ppm. HRMS (ESI) *m/z* $[M+H]^+$ calculated for $C_{26}H_{27}O_2$ 371.2005, found 371.1989.

General procedure for the synthesis of 5a-7c:

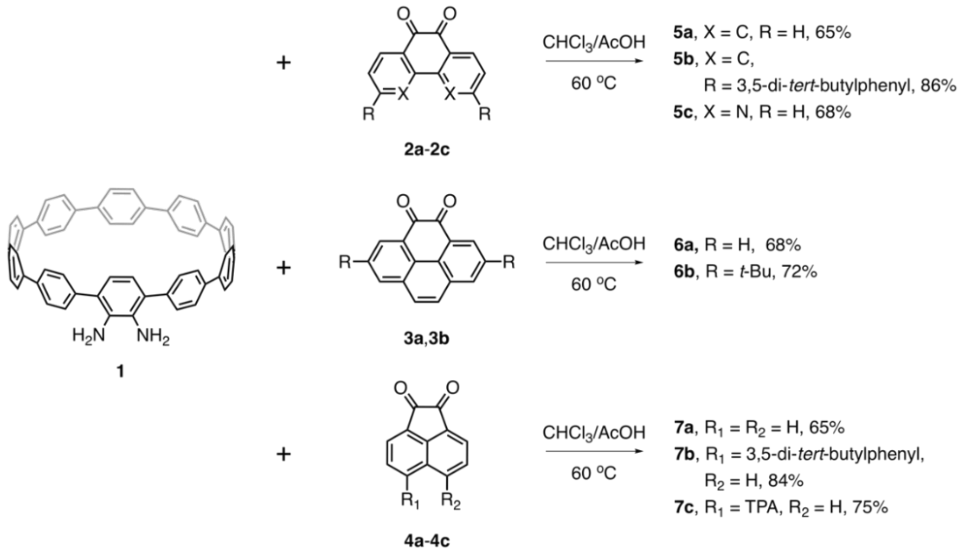

Scheme S1. Synthesis of nanohoops (**5a-7c**) with D-A structure.

Under Argon atmosphere, **1** (20 mg, 0.025 mmol, 1.0 eq.) and diketones (**2a**-**4c**) (0.03 mmol, 1.2 eq.) were added to a 50 mL Schlenk flask. Then 4 mL degassed $CHCl_3$ and 1 mL degassed acetic acid were injected to the flask and heated to 60 °C. The reaction was stirred for 48 hours. For reactions affording **5b** and **6b**, the reaction was quenched by adding deionized water and extracted with DCM. The crude material was washed with brine, dried over $MgSO_4$ and concentrated, which was further purified by column chromatography (silica gel, petroleum ether/DCM = 1/1). For other reactions**,** 20 mL methanol was added to reaction solution after the reaction mixture was cooled down to room temperature. The resulting mixture was sonicated and filtered, and the products were washed three times with methanol and dried under reduce pressure.

5a: ^{1}H NMR (600 MHz, 1,1,2,2-$C_2D_2Cl_4$) δ = 9.37 (d, *J* = 7.8 Hz, 2H), 8.65 (d, *J* = 8.3 Hz, 2H), 7.97 (d, *J* = 8.5 Hz, 4H), 7.87 (t, *J* = 7.7 Hz, 2H), 7.78 (t, *J* = 7.4 Hz, 2H), 7.72 (d, *J* = 8.3 Hz, 4H), 7.69 (d, *J* = 8.3 Hz, 4H), 7.62 – 7.57 (m, 26H) ppm. DEPT 135 ^{13}C NMR (150 MHz, 1,1,2,2-$C_2D_2Cl_4$) δ = 131.99, 130.16, 129.70, 127.89, 127.10, 127.02, 126.93, 126.59, 126.19, 122.75 ppm. HRMS (ESI) *m/z* $[M+Na]^+$ calculated for $C_{74}H_{46}N_2Na$ 985.3553, found 985.3549.

5b: ^{1}H NMR (600 MHz, $CDCl_3$) δ = 9.44 (d, *J* = 8.2 Hz, 2H), 8.91 (s, 2H), 8.30 (d, *J* = 8.1 Hz, 2H), 8.03 (d, *J* = 8.2 Hz, 2H), 7.96 (d, *J* = 8.7 Hz, 4H), 7.71 – 7.69 (m, 6H), 7.67 (d, *J* = 8.7 Hz, 4H), 7.59 (d, *J* = 8.8 Hz, 4H), 7.58 – 7.54 (m, 24H), 1.45 (s, 18H), 1.41 (s, 18H) ppm. DEPT 135 ^{13}C NMR (150 MHz, $CDCl_3$) δ = 132.16, 131.15, 130.03, 128.44, 127.41, 127.31, 127.21, 127.13, 126.88, 123.23, 122.82, 122.13, 121.91, 121.66, 121.61, 31.51 ppm. MS (MALDI-TOF) *m/z* $[M]^+$ calculated for $C_{102}H_{86}N_2$ 1338.679, found 1338.654.

5c:^{1}H NMR (600 MHz, $CDCl_3$) δ = 9.60 (d, *J* = 9.7 Hz, 2H), 9.30 (d, *J* = 6.1 Hz, 2H), 7.88 (d, *J* = 8.7 Hz, 4H), 7.80 (dd, *J* = 8.0, 4.4 Hz, 2H), 7.69 (d, *J* = 8.7 Hz, 4H), 7.65 (d, *J* = 8.8 Hz, 4H), 7.59 (d, *J* = 8.0 Hz, 6H), 7.56 – 7.54 (m, 20H) ppm. DEPT 135 ^{13}C NMR (150 MHz, $CDCl_3$) δ = 152.58, 134.04, 132.94, 129.96, 127.43, 127.31, 127.24, 127.01, 124.28 ppm. HRMS (ESI) *m/z* $[M+Na]^+$ calculated for $C_{72}H_{44}N_4Na$ 987.3458, found 987.3412.

6a: ^{1}H NMR (600 MHz, 1,1,2,2-$C_2D_2Cl_4$) δ = 9.56 (d, *J* = 7.5 Hz, 2H), 8.36 (d, *J* = 8.0 Hz, 2H), 8.14 (d, *J* = 9.9 Hz, 4H), 8.03 (d, *J* = 8.0 Hz, 4H), 7.75 (d, *J* = 8.5 Hz, 4H), 7.70 (d, *J* = 8.0 Hz, 4H), 7.63 – 7.57 (m, 26H) ppm. DEPT 135 ^{13}C NMR (150 MHz, 1,1,2,2-$C_2D_2Cl_4$) δ = 135.79, 132.18, 132.10, 129.90, 129.79, 129.01, 127.77, 127.10, 127.02, 126.93, 126.79, 126.61, 123.96 ppm. HRMS (ESI) *m/z* $[M+H]^+$ calculated for $C_{76}H_{47}N_2$ 987.3734, found 987.3741.

6b: ^{1}H NMR (600 MHz, $CDCl_3$) δ = 9.61 (d, *J* = 1.9 Hz, 2H), 8.29 (d, *J* = 1.9 Hz, 2H), 8.07 (d, *J* = 8.8 Hz, 4H), 8.04 (s, 2H), 7.69 (dd, *J* = 14.3, 8.8 Hz, 8H), 7.62 (s, 2H), 7.58 (s, 2H), 7.56 (s, 2H), 7.55 (d, *J* = 2.5 Hz, 20H), 1.61(s, 18H) ppm. DEPT 135 ^{13}C NMR (600 MHz, $CDCl_3$) δ = 132.12, 130.09, 127.43, 127.36, 127.31, 127.28, 127.18, 126.63, 125.67, 121.70, 31.74 ppm. HRMS (ESI) *m/z* $[M+H]^+$ calculated for $C_{84}H_{62}N_2$ 1099.4986, found 1099.4973.

7a: ^{1}H NMR (600 MHz, 1,1,2,2-$C_2D_2Cl_4$) δ = 8.51 (d, *J* = 6.7 Hz, 2H), 8.19 (d, *J* = 8.3 Hz, 2H), 7.93 – 7.86 (m, 6H), 7.71 – 7.66 (m, 8H), 7.63 – 7.58 (m, 24H), 7.44 (s, 2H) ppm. DEPT 135 ^{13}C NMR (150 MHz, 1,1,2,2-$C_2D_2Cl_4$) δ = 131.85, 129.96, 129.37, 128.77, 128.47, 127.95, 127.09, 127.03, 126.92, 126.54, 125.19, 122.05 ppm. HRMS (ESI) *m/z* $[M+Na]^+$ calculated for $C_{72}H_{44}N_2Na$ 959.3397, found 959.3377

7b: ^{1}H NMR (600 MHz, 1,1,2,2-$C_2D_2Cl_4$) δ = 8.58 (d, *J* = 7.1 Hz, 1H), 8.55 (d, *J* = 6.8 Hz, 1H), 8.31 (d, *J* = 8.4 Hz, 1H), 8.07 (d, *J* = 7.8 Hz, 1H), 7.95 – 7.86 (m, 5H), 7.80 – 7.75 (m, 6H), 7.72 – 7.67 (m, 7H), 7.62 – 7.58 (m, 18H), 7.50 (d, *J* = 4.0 Hz, 4H), 7.45 (s, 2H), 1.44 (s, 18H) ppm. DEPT 135 ^{13}C NMR (150 MHz, 1,1,2,2-$C_2D_2Cl_4$) δ = 129.97, 127.10, 126.93, 126.54, 126.40, 124.12, 31.21 ppm. MS (MALDI-TOF) *m/z* $[M]^+$ calculated for $C_{86}H_{64}N_2$ 1124.507, found 1124.500.

7c: ^{1}H NMR (600 MHz, 1,1,2,2-$C_2D_2Cl_4$) δ = 8.55 (t, *J* = 7.1 Hz, 2H), 8.38 (d, *J* = 8.6 Hz, 1H), 7.89 (d, *J* = 8.5 Hz, 5H), 7.69 (dd, *J* = 13.5, 8.4 Hz, 8H), 7.62 – 7.56 (m, 26H), 7.44 (s, 2H), 7.35 (t, *J* = 7.5 Hz, 5H), 7.28 (d, *J* = 8.2 Hz, 2H), 7.25 (d, J = 8.2 Hz, 4H), 7.11 (t, *J* = 7.4 Hz, 2H) ppm. DEPT 135 ^{13}C NMR (150 MHz, 1,1,2,2-$C_2D_2Cl_4$) δ = 131.81, 131.71, 130.60, 129.96, 129.17, 129.10, 128.81, 128.50, 128.36, 127.03, 126.93, 126.54, 124.77, 124.46, 123.00, 122.54, 122.16, 121.99 ppm. MS (MALDI-TOF) *m/z* $[M]^+$ calculated for $C_{90}H_{57}N_3$ 1179.455, found 1179.443.

Procedure for the synthesis of 5d:

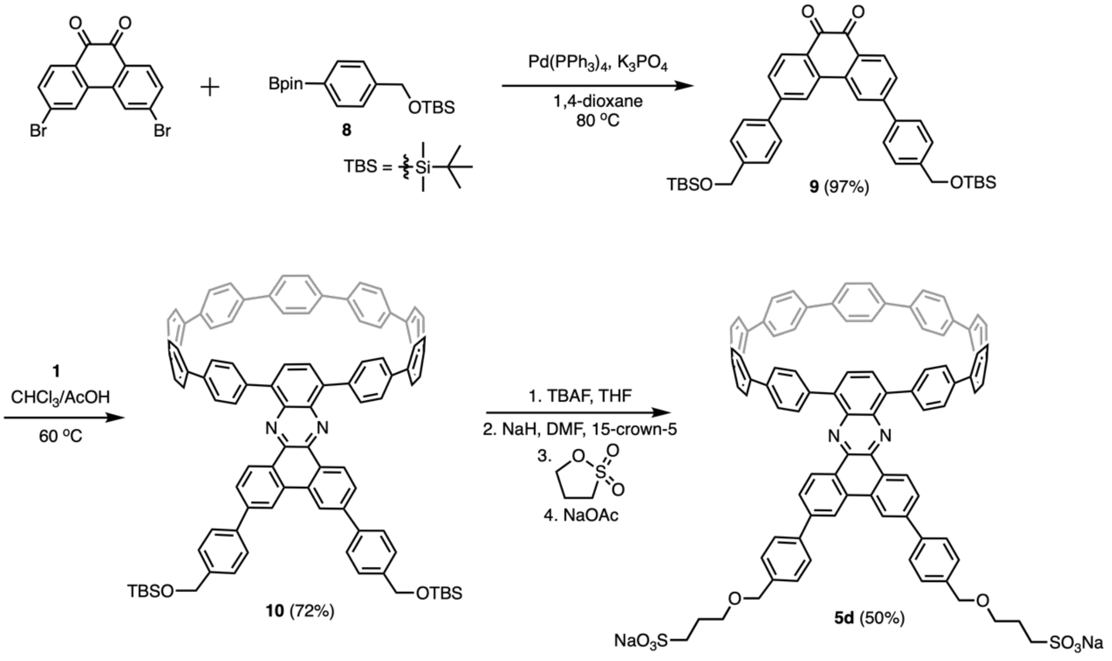

Scheme S2. Synthesis of a water-soluble nanohoop (**5d**) with two water solubilizing sulfonate substituents.

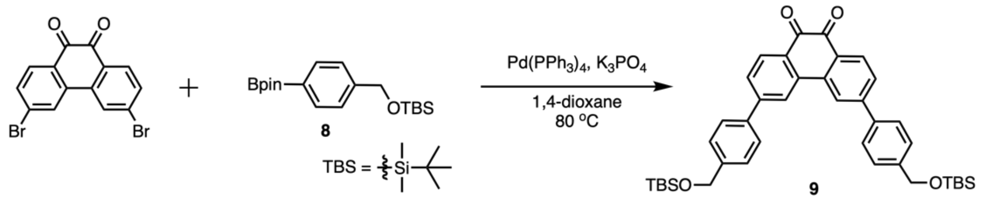

To a 100-mL round-bottom-flask containing a magnetic stirring bar were added compound **8** (238 mg, 0.68 mmol, 2.5 eq.), 3,6-dibromophenanthrene-9,10-dione (100 mg, 0.27 mmol, 1.0 eq.), and $Pd(PPh_3)_4$ (19 mg, 0.02 mmol, 0.07 eq.) under Argon atmosphere. Then degassed 1,4-dioxane (11 mL) was added to the round-bottom-flask. The prepared mixture was heated for 10 minutes at 80 °C before degassed K_3PO_4 solution (1.1 mL, 2 M in deionized water) was added. The solution was then stirred at 80 °C overnight. After the reaction mixture was cooled down to room temperature, the residue was extracted with $CHCl_3$, dried over Na_2SO_4, and concentrated under reduced pressure. The crude product was purified by silica gel column chromatography (Petroleum ether/EtOAc = 4:1 to 1:1) to afford **9** (171 mg, 97%). ^1H NMR (600 MHz, $CDCl_3$) δ = 8.27 (d, J = 8.1 Hz, 4H), 7.68 (d, J = 6.5 Hz, 6H), 7.51 (d, J = 7.9 Hz, 4H),

4.84 (s, 4H), 0.98 (s, 18H), 0.15 (s, 12H) ppm. ^{13}C NMR (150 MHz, $CDCl_3$) δ = 180.00, 148.68, 142.67, 138.05, 136.12, 131.14, 129.81, 128.17, 127.18, 126.69, 122.49, 77.16, 64.51, 29.63, 25.90, 18.38 ppm. HRMS (ESI) *m/z* $[M+H]^+$ calculated for $C_{40}H_{49}O_4Si_2$ 649.3169, found 649.3176.

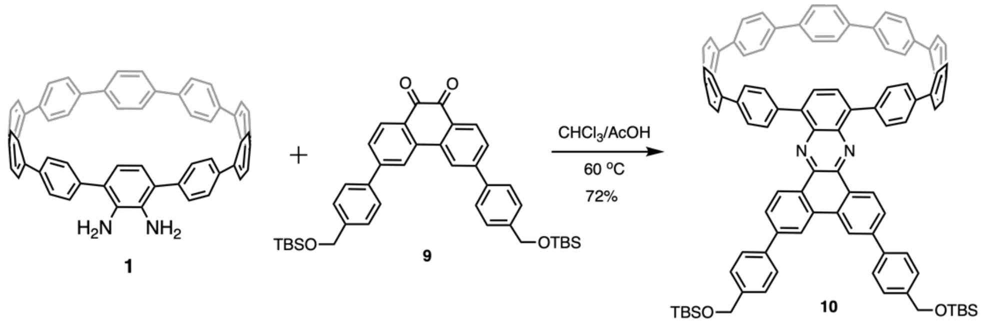

Under Argon atmosphere, **1** (20 mg, 0.025 mmol, 1.0 eq.) and compound **9** (21 mg, 0.03 mmol, 1.2 eq.) were added to a 50 mL Schlenk flask. Then 4 mL degassed $CHCl_3$ and 1 mL degassed acetic acid were injected to the flask and the reaction mixture was heated to 60 °C and stirred for 48 hours. After the reaction mixture was cooled down to room temperature, methanol (20 mL) was added to reaction solution and the resulting mixture was sonicated and filtered, the crude material was washed three times with methanol and dried under reduce pressure to give compound **10** (25 mg, 72%). ^{1}H NMR (400 MHz, $CDCl_3$) δ = 9.42 (d, *J* = 8.2 Hz, 2H), 8.86 (s, 2H), 7.96 (dd, *J* = 16.2, 8.3 Hz, 6H), 7.82 (d, *J* = 7.8 Hz, 4H), 7.68 (dd, *J* = 19.7, 8.5 Hz, 8H), 7.62 – 7.50 (m, 30H), 4.87 (s, 4H), 1.00 (s, 18H), 0.17 (s, 12H) ppm. DEPT 135 ^{13}C NMR (100 MHz, $CDCl_3$) δ = 132.21, 130.03, 127.41, 127.31, 127.22, 126.88, 126.64, 121.41, 64.66, 25.96 ppm. MS (MALDI-TOF) *m/z* $[M]^+$ calculated for $C_{100}H_{86}N_2O_2Si_2$ 1403.970, found 1404.145.

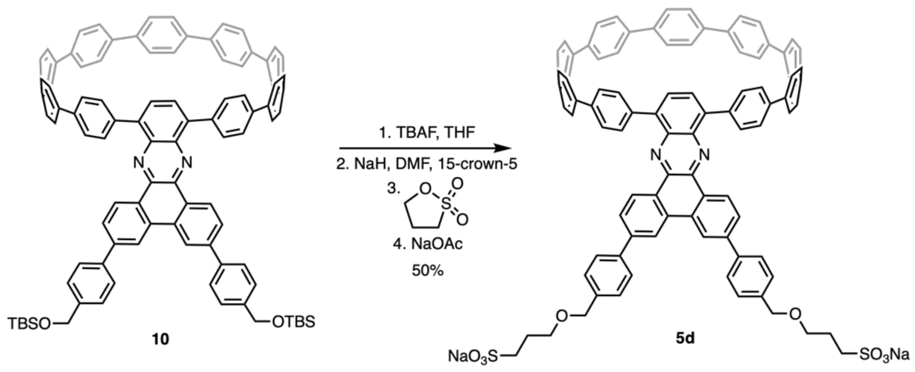

Under Argon atmosphere, compound **10** (25 mg, 0.02 mmol, 1.00 eq.) was added to a flame-dried flask and dissolved in anhydrous THF (3 mL). The reaction mixture was cooled to 0 °C for 10 minutes. Then *tert*-butyl ammonium fluoride (TBAF) (1.0 M in THF, 0.40 mL, 0.40 mmol, 20.0 eq.) was added dropwise. The resulting solution was allowed to slowly warm to room temperature stirred for 2 hours before quenching with deionized water (5 mL). The mixture was concentrated to remove the THF and then was collected by vacuum filtration and rinsed with deionized water and methanol. The resulting yellow solid was dried thoroughly with high vacuum and then was suspended in DMF (1.5 mL) in a flame-dried flask at room temperature. The solution was stirred for 15 minutes to promote dissolution of the nanohoop, and then sodium hydride (4 mg, 0.1 mmol, 5.00 eq.) was added to the mixture. The suspension was stirred for 30 minutes, then 15-crown-5 (25 µL, 0.16 mmol, 8.00 eq.) was added to the flask and stirred for another 30 minutes. The suspension was added to neat 1,3-propane sultone (0.02 mL, 0.2 mmol, 10 eq.) and the reaction mixture became a light orange color. After stirring for 16 hours, the reaction was quenched with the addition of deionized water (5.00 mL) and the mixture was further stirred for 15 minutes. Sodium acetate (24 mg, 0.3 mmol, 15.0 eq.) was added to the light yellow solution and the mixture was stirred for an additional 30 minutes. The crude product was filtered and then washed with ethyl acetate and methanol to give **5d** (14 mg, 50%). ^{1}H NMR (400 MHz, $CDCl_3$) δ = 9.44 (d, J = 8.3 Hz, 4H), 8.86 (s, 4H), 8.03-7.94 (m, 10H), 7.86 (d, J = 7.9 Hz, 6H), 7.71 – 7.65 (m, 10H), 7.60 – 7.52 (m, 18H), 4.84 (d, J = 5.8 Hz, 4H), 3.69 (s, 4H), 3.48 (d,

J = 5.1 Hz, 4H), 1.78 – 1.75 (m, 4H) ppm. ^{13}C NMR spectrum could not be obtained due to low solubility. HRMS (ESI) *m/z* $[M-Na]^-$ calculated for $C_{94}H_{68}N_2NaO_8S_2^-$ 1439.4320, found 1439.4625.

2. Electrochemical Analysis

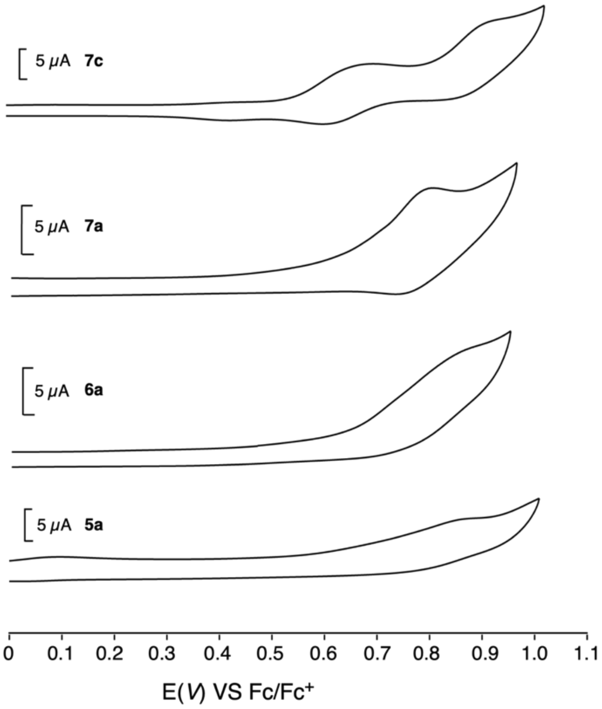

Figure S1. Cyclic voltammograms of **5a**, **6a**, **7a** and **7c** in anhydrous DCM containing *n*-BuNPF$_6$ as electrolyte.

Table S1. Oxidation potentials of cyclic voltammograms of **5a**, **6a**, **7a** and **7c**.

	**5a**	**6a**	**7a**	**7c**
$E_{1/2}^{ox}$ (V)	0.60	0.61	0.63	0.54

3. X-ray crystallographic data

The single crystals of **6a**⊃C$_{60}$ suitable for X-ray crystallographic analysis were obtained by slow diffusion of *n*-hexane into a CHCl$_3$ solution of **6a** and C$_{60}$ at -20 °C in air. The single crystal X-ray diffraction studies were performed on the XtaLAB Synergy diffractometer using Cu Kα radiation (λ = 1.54184 Å) equipped with Hypix6000HE detector. The dark crystal was mounted on a goniometer head with the help of glycerol and a nylon loop, which was immediately transferred to diffractometer. The obtained diffraction data were processed by the program suite CrysAlispro (Rigaku Oxford Diffraction, CrysAlisPro Software system, version 1.171.40_64.53, Rigaku Corporation, Oxford, UK, 2019). The structures were solved with dual-space method using SHELXT program[6] and refined with non-linear least square method using SHELXL program[7] embedded in the program suite OLEX2.[8] The detailed diffraction

data collection and refinement parameters of **6a**⊃C_{60} were listed in Table S2. The crystallographic data were deposited in Cambridge Crystallographic Data Centre (CCDC 2158588). The data can be achieved free of charge from www.ccdc.cam.au.uk/data_request/cif.

Table S2. Crystal data and structure refinement for **6a**⊃C_{60} at 160 K.

CCDC No.	2158588
Empirical formula	$C_{154.40}$ $H_{88.40}$ $Cl_{1.20}$ N_2
Formula weight	2014.01
Temperature	160(2) K
Wavelength	1.54184 Å
Crystal system	orthorhombic
Space group	$P2_12_12_1$
Unit cell dimensions	a = 16.6132(6) Å<br>b = 18.7606(12) Å<br>c = 32.1149(13) Å<br>α = 90°<br>β = 90°<br>γ = 90°
Volume	10009.4(8) $Å^3$
Z	4
Density (calculated)	1.336 g/cm^3
Absorption coefficient	0.870 mm^{-1}
F(000)	4197
Crystal size	0.05 × 0.05 × 0.05
Theta range for data collection	2.7330° to 51.4110°
Limiting indices	$-17 \leq h \leq 17, -20 \leq k \leq 17, -34 \leq l \leq 30$
Reflections collected	25299
Independent reflections	11961 [R_{int} = 0.0786, R_{sigma} =0.1162]
Radiation	Cu Kα (λ = 1.54184)
Data / restraints / parameters	11961 / 585 /1608
Goodness-of-fit on F2	0.968
Final R indices [I>2σ(I)]	R_1 = 0.0742, wR_2 = 0.1687
R indices (all data)	R_1 = 0.1498, wR_2 = 0.2103
Largest diff. peak and hole	0.243 and -0.270 e • $Å^{-3}$

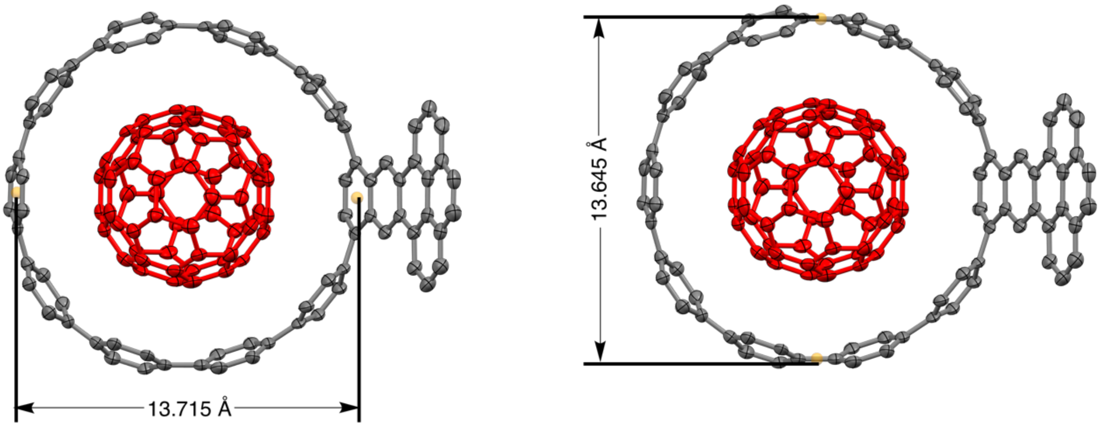

Figure S2. ORTEP drawing of **6a**⊃C_{60}. Thermal ellipsoids are set with 30% probability. Solvent molecules and hydrogen atoms are omitted.

Table S3. Dihedral angles of **6a**⊃C_{60}.

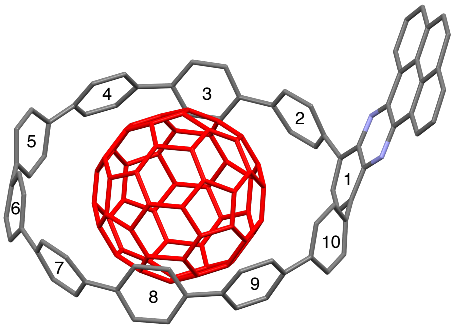

	**2&3**	**3&4**	**4&5**	**5&6**	**6&7**	**7&8**	**8&9**	**9&10**	**1&2**	**1&10**
dihedral angle (°)	38.36	48.33	49.77	44.31	44.71	46.41	45.00	41.16	54.37	52.21

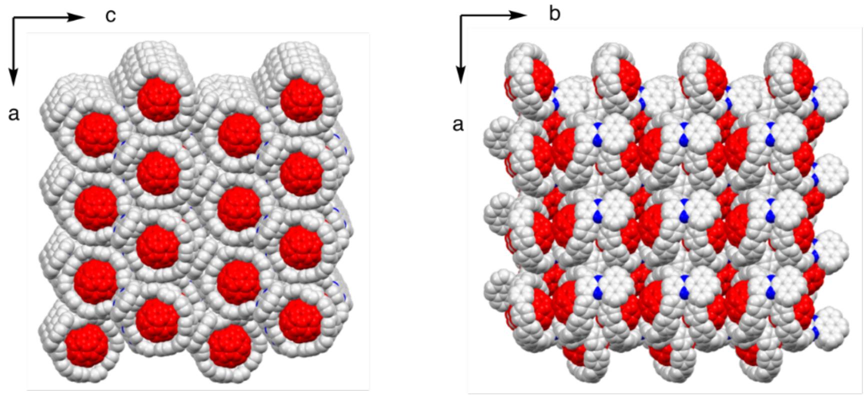

Figure S3. Packing structure of complexes of **6a**⊃C_{60}. **6a** and C_{60} were colored in gray and red, respectively.

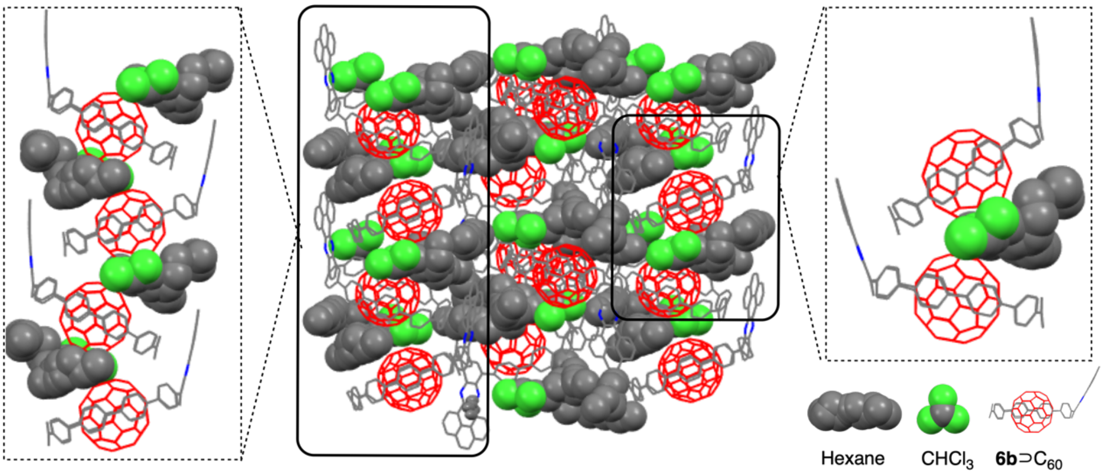

Figure S4. Packing structure of complexes of **6a**⊃C_{60} with solvents. **6a** and C_{60} were colored in dark gray and red (wireframe model), solvents were colored in gray and green (spacefill model), respectively.

4. Complexation with C_{60}

Fluorescence quenching experiments were conducted by adding C_{60} to nanohoop hosts to afford binding constants K_a in *o*-DCB. A 4-mL-solution of host in *o*-DCB (5.0×10^{-7} mol/L) was prepared, and then 1 mL of the solution was used to prepare the C_{60} (guest) solution (2.50×10^{-5} mol/L). The remaining solution of the host is then transferred to a cuvette and the C_{60} (guest) solution is added to the cuvette by using a Microliter Syringes. The changes in the fluorescent intensity at 375 nm were used for obtaining binding constants. The K_a values of the four nonahoops (**5a**, **6a**, **7a**, **7c**) were obtained using *Bindfit* developed by Thordarson[9] from triplicated titrations for each sample.

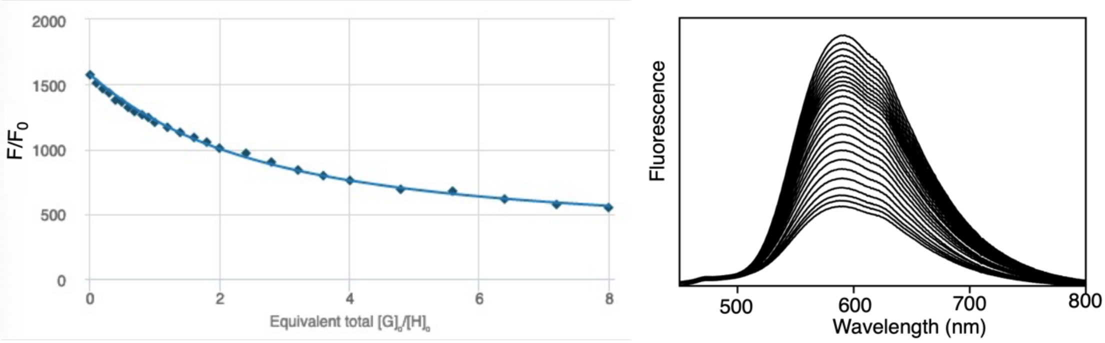

Figure S5. Curve-fitting of **5a** in *o*-DCB to a 1:1 binding model to afford $K_a = (9.10 \pm 0.12) \times 10^5$ M^{-1}.

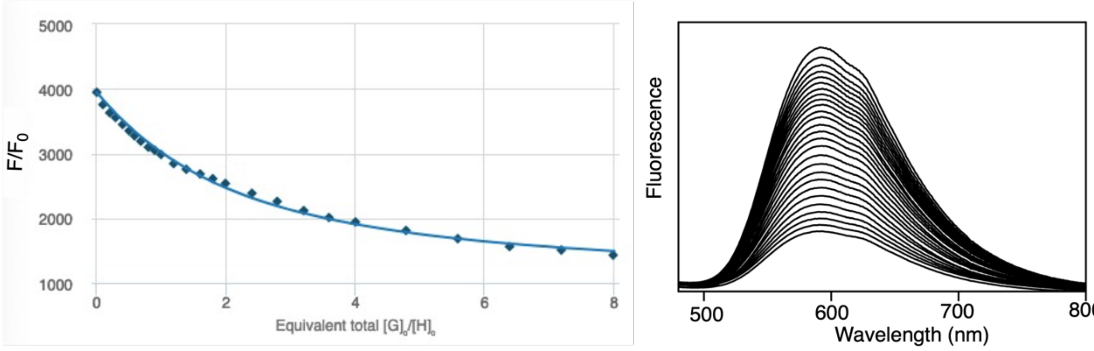

Figure S6. Curve-fitting of **6a** in *o*-DCB to a 1:1 binding model using Bindfit to afford $K_a = (1.05 \pm 0.21) \times 10^6$ M^{-1}.

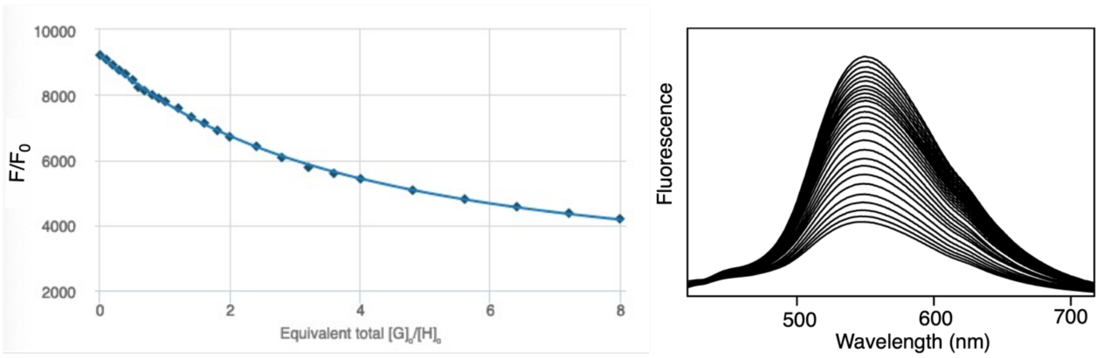

Figure S7. Curve-fitting of **7a** in *o*-DCB to a 1:1 binding model using Bindfit to afford K_a = (7.69 ± 0.12) × 10^5 M^{-1}.

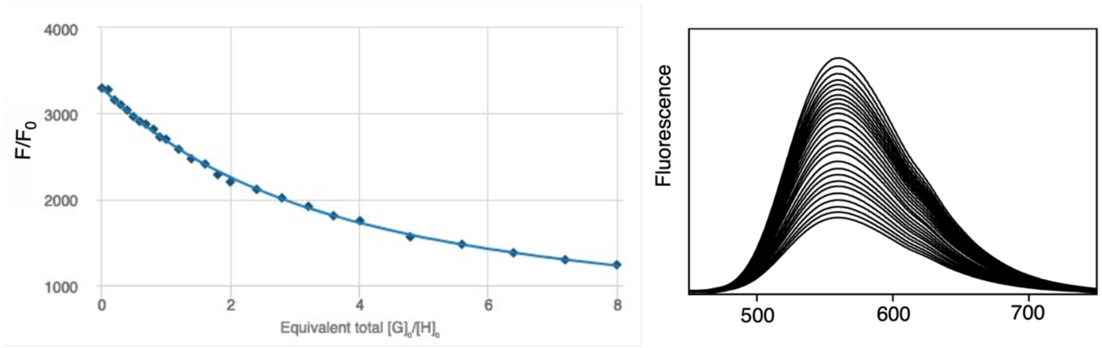

Figure S8. Curve-fitting of **7c** in *o*-DCB to a 1:1 binding model using Bindfit to afford K_a = (7.61 ± 0.60) × 10^5 M^{-1}.

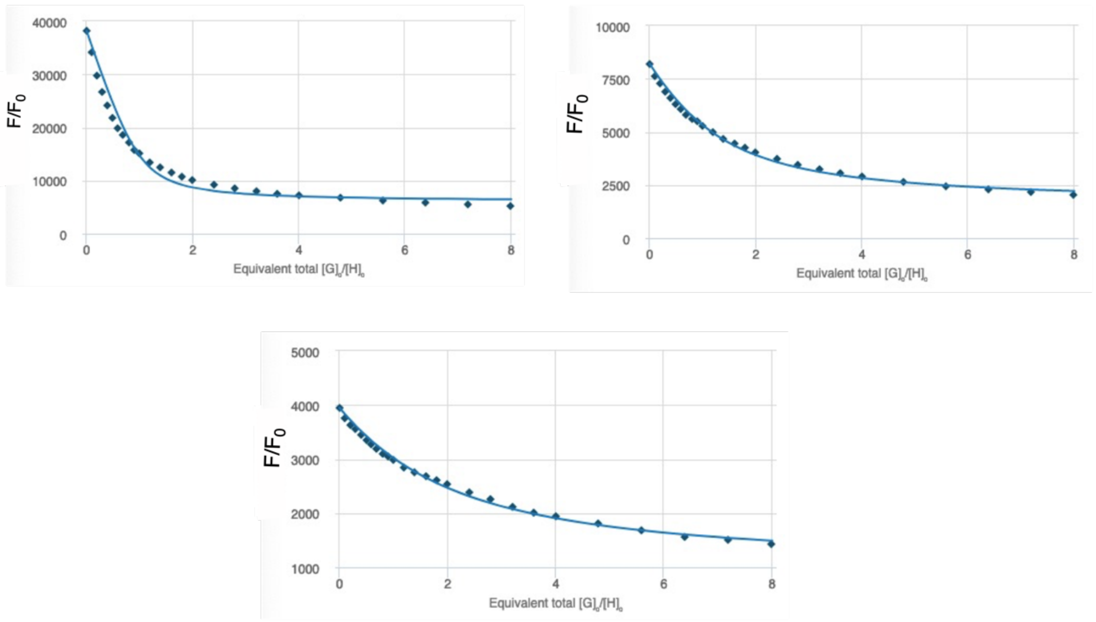

Figure S9. Curve-fitting of **6a** in *o*-DCB for obtaining the K_a (M^{-1}) to a 1:1 binding model using Bindfit at three different concentrations (5.0 × 10^{-6} mol L^{-1}, 1.0 × 10^{-6} mol L^{-1}, 5.0 × 10^{-7} mol L^{-1}), R^2 measures the goodness of fit.

Table S4. Association constants of **6a** towards the C_{60} determined by global nonlinear regression analysis to 1:1 models by three different concentrations of **6a**.[9]

Concentration (M)	cov_{fit}	K_a (M^{-1})
5.0×10^{-7}	$(2.70 \pm 1.96) \times 10^{-3}$	$(1.06 \pm 0.21) \times 10^{6}$
5.0×10^{-6}	$(3.64 \pm 0.99) \times 10^{-2}$	$(2.26 \pm 0.19) \times 10^{6}$
1.0×10^{-6}	$(5.17 \pm 0.36) \times 10^{-3}$	$(1.47 \pm 0.19) \times 10^{6}$

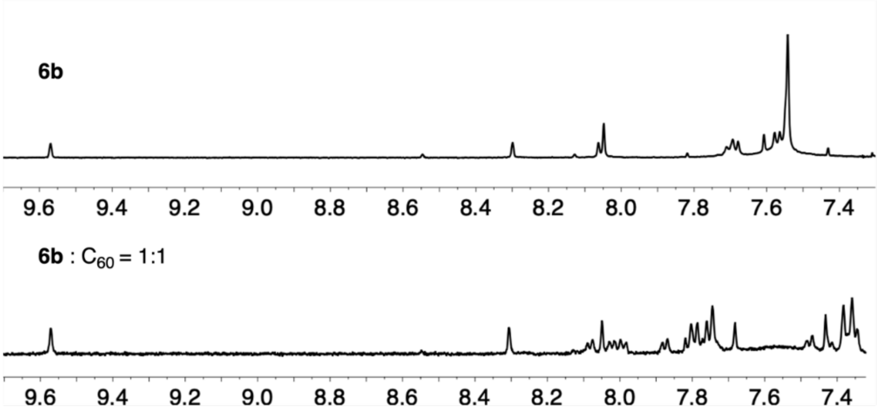

Figure S10. ^{1}H NMR spectra of **6b** and **6b**⊃C_{60} ($CDCl_3$, 600 MHz, 243 K).

5. Additional spectroscopic data

5.1 Steady spectroscopic data

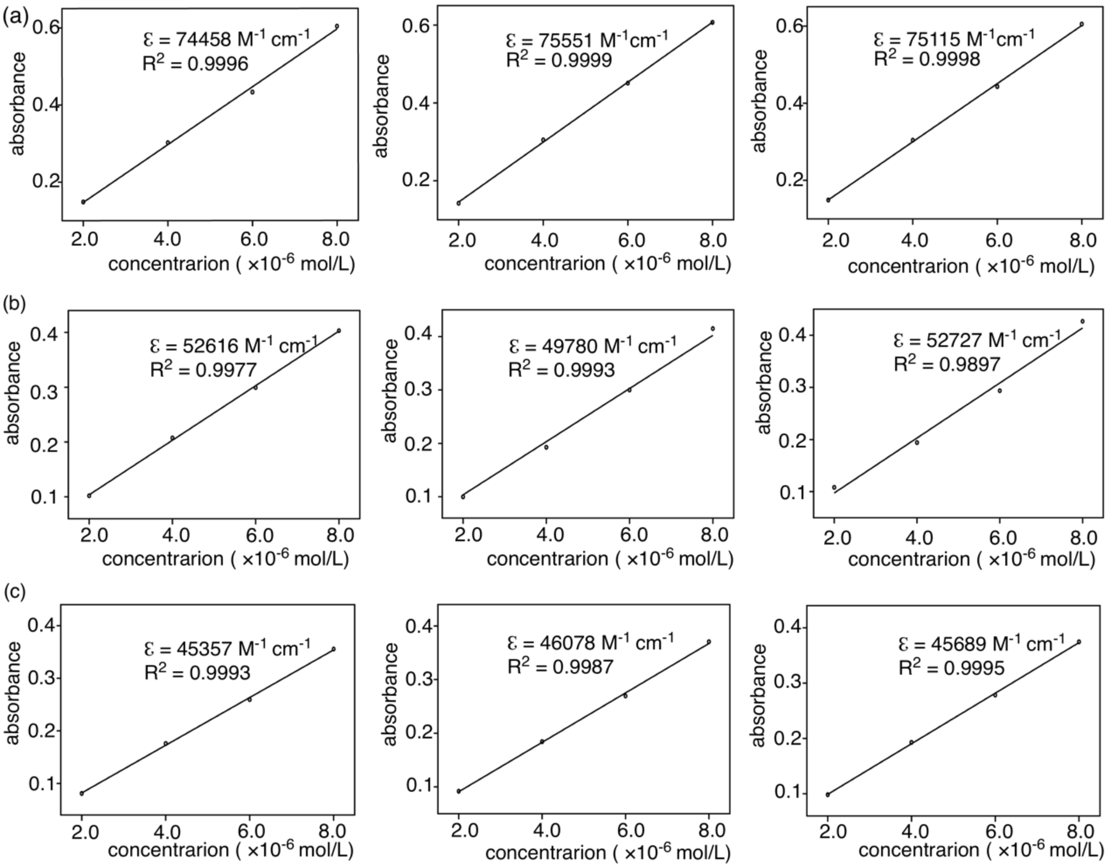

Figure S11. Absorbance versus concentration for extinction coefficient determination of **5a** (a), **5b** (b) and **5c** (c) at absorbance maximum with triplicate experiments.

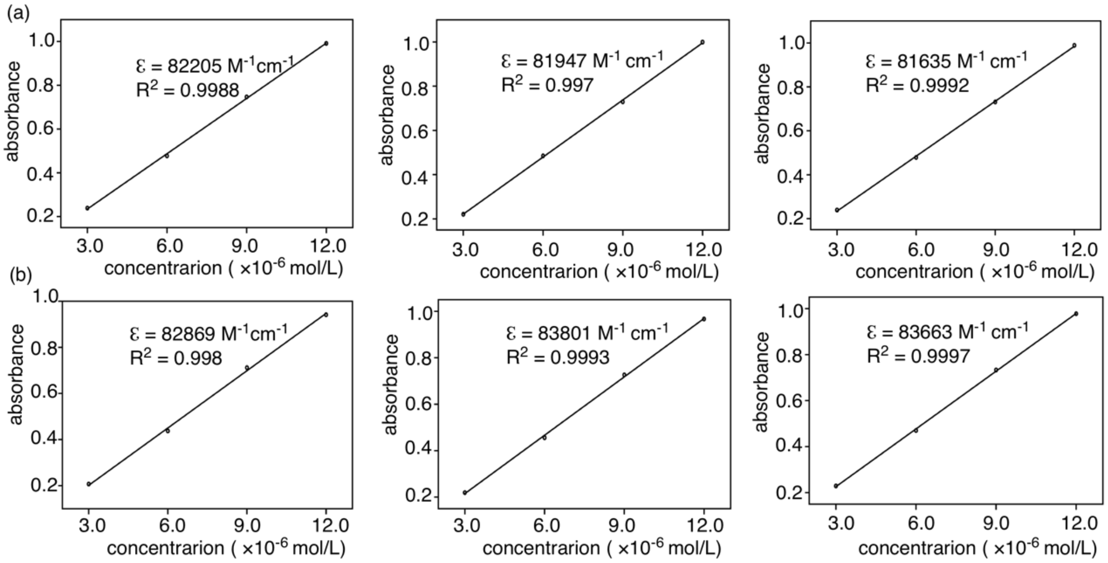

Figure S12. Absorbance versus concentration for extinction coefficient determination of **6a** (a), **6b** (b) at absorbance maximum with triplicate experiments.

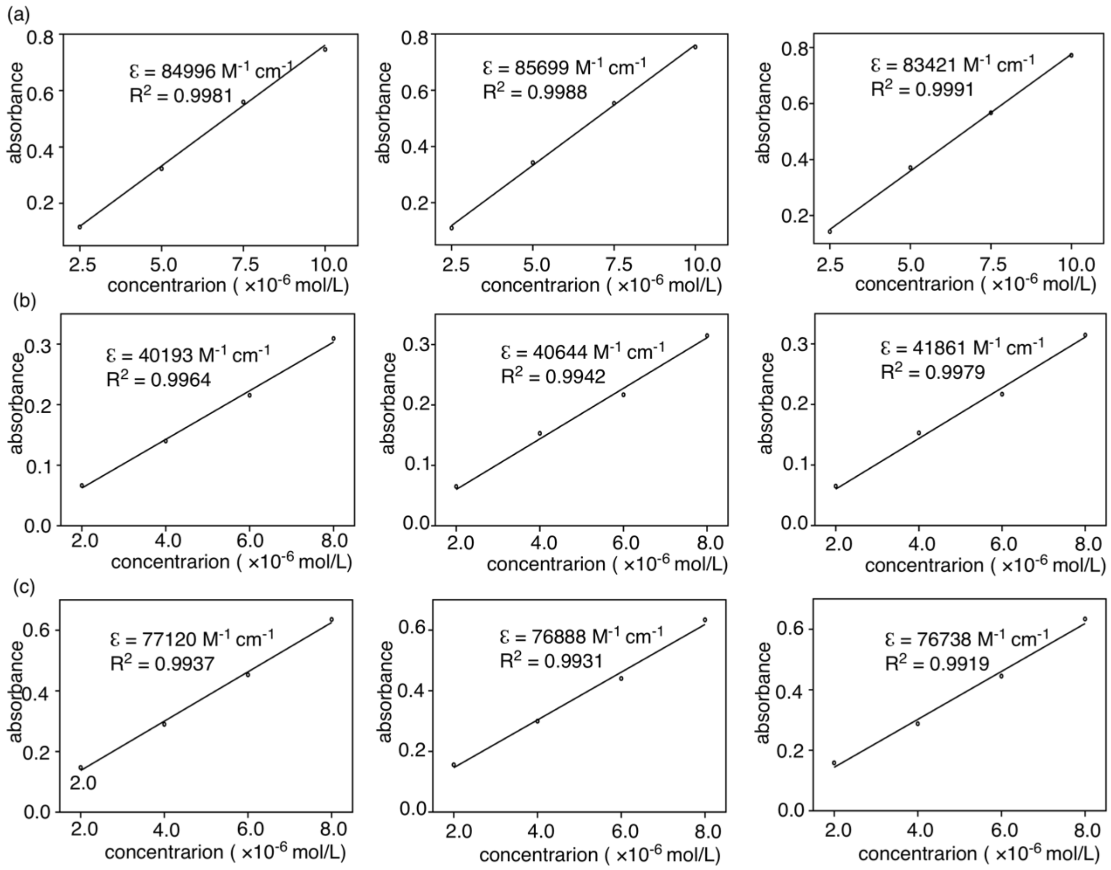

Figure S13. Absorbance versus concentration for extinction coefficient determination of **7a** (a), **7b** (b) and **7c** (c) at absorbance maximum with triplicate experiments.

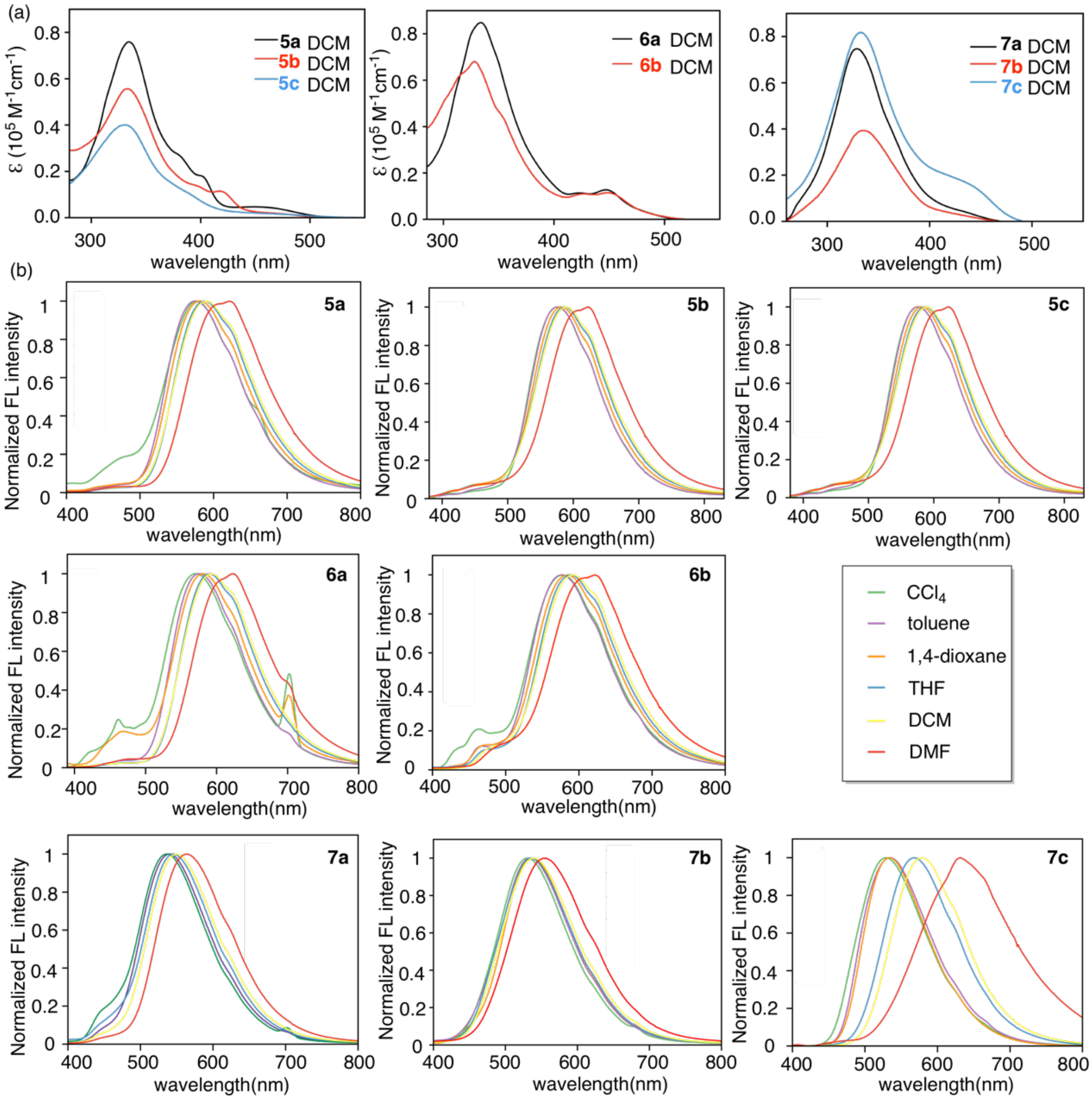

Figure S14. Absorption spectra of all eight nanohoops (a), fluorescence spectra of **5a-5c** (b), **6a,6b** (c) and **7a-7c** (d) in different solvents.

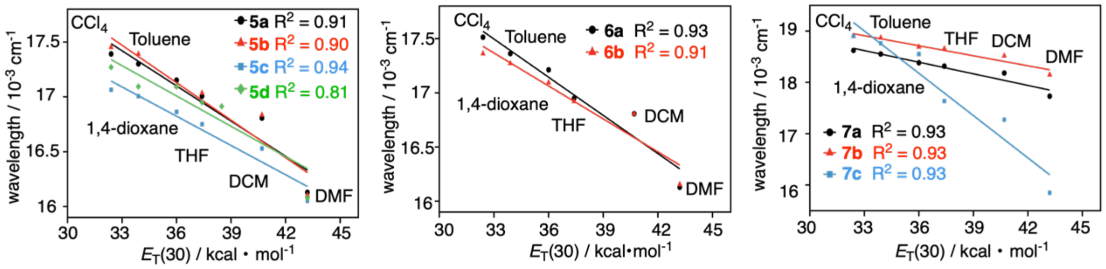

Figure S15. A plot of emission maxima (wavenumber) of **5a-5d**, **6a-6b** and **7a-7c** in various solvents against $E_T(30)$.

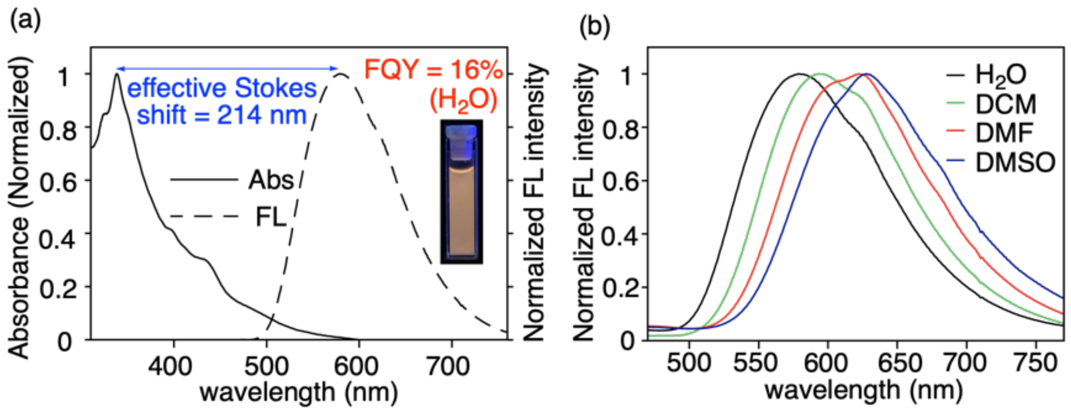

Figure S16. (a) Absorption spectra and fluorescence spectra of **5d** in H_2O; (b) Fluorescence spectra of **5d** in different solvents.

5.2 Solvatochromic behaviors

The solvent and solute interaction, *i.e.* solvation, affects the energy level of the lowest-lying excited state and its fluorescent emission. Here, we employed the Stokes shift against the orientation polarizability (Δf) using the Lippert−Mataga equation[10, 11]

$$\bar{\nu}_{abs} - \bar{\nu}_{em} = \frac{2}{hc}\left(\frac{\varepsilon - 1}{2\varepsilon + 1} - \frac{n^2 - 1}{2n^2 + 1}\right)\frac{(\mu_{S1} - \mu_{S0})^2}{a^3} + cons$$

$$= \frac{2}{hc}\frac{(\mu_{S1} - \mu_{S0})^2}{a^3}\Delta f + cons \qquad \text{Eq. S1}$$

where $\bar{\nu}_{\mathrm{abs}}$ and $\bar{\nu}_{\mathrm{em}}$ are the wavenumbers (cm^{-1}) of the absorption and emission spectral peaks, respectively, *h* is the Planck's constant, *c* is the speed of light, *a* is the radius of Onsager cavity around the fluorophore, *ε* and *n* are the dielectric constant and refractive index of the solvent, respectively, and $\mu_{\mathrm{S1}} - \mu_{\mathrm{S0}}$ ($\Delta\mu$) is the dipole moment difference between the excited state (μ_{S1}) and ground state (μ_{S0}). The Lippert−Mataga relationship between Stokes shifts ($\bar{\nu}_{\mathrm{abs}} - \bar{\nu}_{\mathrm{em}}$) of the synthesized compounds and solvent polarity are shown in Figure S17. According to quantum chemical calculations described as described in section 5, the Onsager radiuses and μ_{S0} of **7a-c** are obtained. From the slopes of the Lippert−Mataga plots, $\Delta\mu$ and μ_{S1} of **7a-c** are estimated. These results are summarized in Table S5.

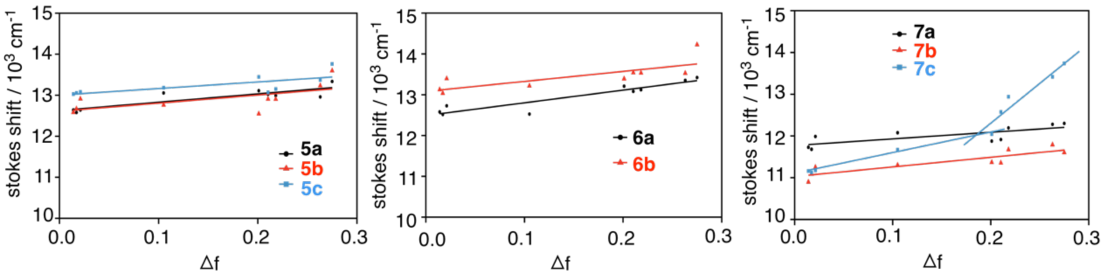

Figure S17. The solvatochromic Lippert-Mataga models of **5a-5c**, **6a-6b** and **7a-7c**.

Table S5. Calculated parameters of **7a-c** by TD-DFT (M06-2X/6-311G**) and Lippert−Mataga plots fitting.

	Calculated a (Å)	Estimated $\Delta\mu$ (Debye)	Calculated μ_{S0} (Debye)	Estimated μ_{S1} (Debye)
**7a**	7.64	8.37	2.58	10.95
**7b**	8.38	11.61	3.40	15.01
**7c**	8.27	16.70 [a]	4.28	20.98 [a]
		32.82 [b]		37.10 [b]

[a]$\Delta\mu$ and μ_{S1} estimated from the slope of Lippert−Mataga model fitted in the low polar regime (Δf < 0.2); [b]$\Delta\mu$ and μ_{S1} estimated from the slope of Lippert−Mataga model fitted in the high polar regime (Δf > 0.2).

5.3 Fluorescence lifetime measurements

Fluorescence lifetimes (Figure S18) were determined using a time correlated single-photon counting (TCSPC) spectrometer (PTI QuantaMaster 800, HORIBA) with a 340 nm nanosecond LED excitation source, the instrument response function (IRF) was measured to be 0.6 ns width.

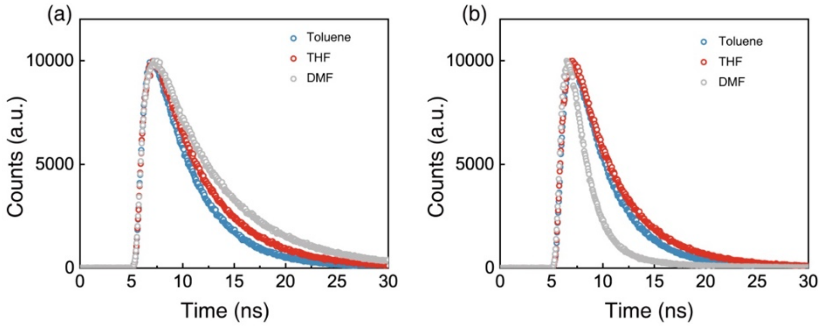

Figure S18. Fluorescence decay kinetics of **7a** (a) and **7c** (b) in different solvents. The fitted fluorescence lifetimes of **7a** were 4.47 ns, 5.39 ns and 6.74 ns for toluene, THF and DMF, respectively. The fitted fluorescence lifetimes of 7c were 3.98 ns, 4.72 ns and 1.31 ns for toluene, THF and DMF, respectively.

6. Theoretical calculations

6.1 General Information

All electronic structure calculations were performed by using Gaussian 09 software package.[12] The ground and excited-state geometries of [10]CPP, **5a**, **6a**, **7a** and **7c** were optimized at M06-2X/6-311G** level.[13] All optimized structures were confirmed to be true minima by vibrational analysis with no imaginary frequency. The polarized continuum model (PCM, toluene) was applied to include solvation in all electronic structure calculations. The vertical transition of low-lying excited-states (S_1~S_4) of [10]CPP, **5a**, **6a**, **7a** and **7c** were summarized in Table S6 to S9. The natural transition orbital (NTO) and hole-electron distribution analysis (Figure S20) were performed by Multiwfn software.[14] The charge transfer weight (CT%) of each excited-states was quantitatively evaluated by calculating the acceptor fragment contributions to hole/electron wave functions (Table S10).[15, 16] Molecular orbital diagram of **6a**$\supset C_{60}$ and [10]CPP$\supset C_{60}$ shown in Figure S19 were obtained from DFT calculation at LC-BLYP/6-311G* with basis set superposition errors (BSSE) correction.[17]

6.2 Vertical transition properties

Table S6. The TD-DFT (M06-2X/6-311G**, PCM = toluene) calculated vertical transition of low-lying (S_1~S_4) excited-states of [10]CPP.

State	Energy (eV)	Wavelength (nm)	Oscillator Strength	Major Contributions
S_1	3.7733	328.58	0.0000	H→L (64%)<br>H-1→L+2 (10%)<br>H-2→L+1 (10%)
S_2	4.1322	300.04	2.6036	H-1→L (46%)<br>H→L+1 (23%)<br>H→L+2 (20%)
S_3	4.1326	300.02	2.6026	H-2→L+1 (45%)<br>H→L+2 (24%)<br>H→L+1 (20%)
S_4	4.7140	263.01	0.0000	H→L+3 (22%)<br>H-1→L+2 (18%)<br>H-2→L+1 (18%)

Table S7. The TD-DFT (M06-2X/6-311G**, PCM = toluene) calculated vertical transition of low-lying (S_1~S_4) excited-states of **5a**.

State	Energy (eV)	Wavelength (nm)	Oscillator Strength	Major Contributions
S_1	3.1765	390.32	0.3064	H→L (64%)<br>H-1→L(34%)
S_2	3.4655	357.76	0.0074	H-17→L (42%)<br>H-7→L (35%)<br>H-11→L (10%)
S_3	3.7241	332.92	0.3694	H→L+1 (58%)<br>H-2→L+2 (12%)
S_4	3.7706	328.82	1.3042	H-4→L (74%)<br>H-2→L (15%)

Table S8. The TD-DFT (M06-2X/6-311G**, PCM = toluene) calculated vertical transition of low-lying (S_1~S_4) excited-states of **6a**.

State	Energy (eV)	Wavelength (nm)	Oscillator Strength	Major Contributions
S_1	3.1828	389.54	0.2898	H→L (63%)<br>H-1→L(35%)
S_2	3.4693	357.38	0.1537	H-4→L (35%)<br>H-18→L (26%)<br>H-7→L (22%)
S_3	3.4905	355.20	0.4356	H-3→L (59%)<br>H-18→L (19%)
S_4	3.7241	332.92	0.3324	H-1→L+1 (59%)<br>H-2→L+3 (14%)

Table S9. The TD-DFT (M06-2X/6-311G**, PCM = toluene) calculated vertical transition of low-lying (S_1~S_4) excited-states of **7a**.

State	Energy (eV)	Wavelength (nm)	Oscillator Strength	Major Contributions
S_1	3.3005	375.70	0.1603	H→L (47%)<br>H-1→L (34%)
S_2	3.4710	357.25	0.0080	H-7→L (51%)<br>H-17→L (29%)
S_3	3.5901	345.39	1.0510	H→L+2 (66%)<br>H-1→L (16%)
S_4	3.6597	338.83	0.2025	H-1→L+1 (40%)<br>H→L+1 (32%)

Table S10. The TD-DFT (M06-2X/6-311G**, PCM = toluene) calculated vertical transition of low-lying (S_1~S_4) excited-states of **7c**.

State	Energy (eV)	Wavelength (nm)	Oscillator Strength	Major Contributions
S_1	3.2590	380.48	0.2885	H→L (39%)<br>H-3→L (16%)<br>H-1→L+1 (13%)<br>H-3→L+1 (11%)
S_2	3.3421	371.02	1.0278	H→L+1 (36%)<br>H→L (27%)<br>H-1→L (18%)
S_3	3.4929	355.01	0.0021	H-8→L+1 (28%)<br>H-8→L (26%)<br>H-20→L+1 (16%)<br>H-20→L (15%)
S_4	3.5852	345.87	0.7621	H-2→L+3 (36%)<br>H-1→L+2 (20%)<br>H-3→L+1 (13%)

6.3 Molecular orbital diagram of 6a⊃C_{60} from TD-DFT calculation

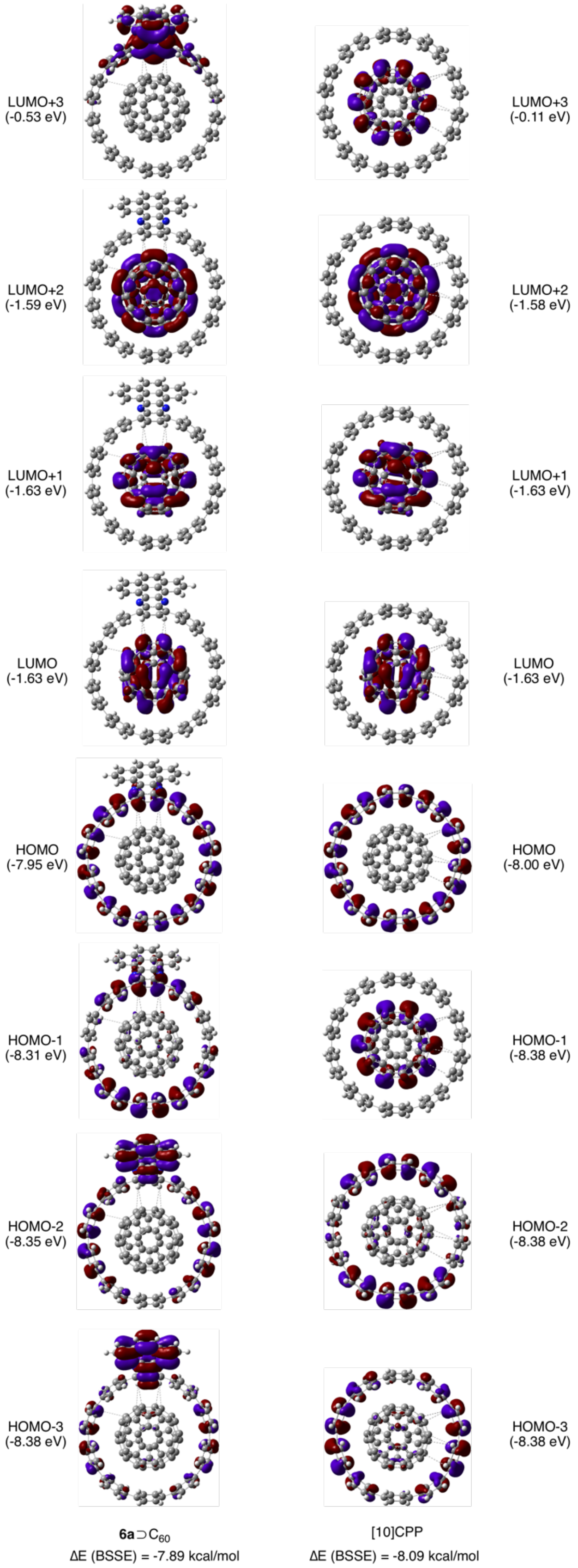

Figure S19. Molecular orbital diagram of **6a**⊃C_{60} and [10]CPP⊃C_{60} from TD-DFT calculation at LC-BLYP/6-311G* with BSSE correction.

6.4 Quantitative parameters for hole-electron analysis

Table S11. The calculated charge transfer parameters from hole-electron analysis of **7a** and **7c** by TD-DFT (M06-2X/6-311G**, PCM = toluene).

<table>
<tr><th></th><th></th><th>[a]Hole</th><th>[b]Electron</th><th>[c]Overlap</th><th>[d]CT%</th><th>[e]LE%</th></tr>
<tr><td rowspan="4">7a (CPP-A)</td><td>S_1</td><td>61.02 %</td><td>36.70 %</td><td>47.32 %</td><td>24.32 %</td><td>75.68%</td></tr>
<tr><td>S_2</td><td>11.23 %</td><td>6.20 %</td><td>8.35 %</td><td>5.03 %</td><td>94.97%</td></tr>
<tr><td>S_3</td><td>87.65 %</td><td>81.63 %</td><td>84.58 %</td><td>6.02 %</td><td>93.98%</td></tr>
<tr><td>S_4</td><td>31.69 %</td><td>5.62 %</td><td>13.35 %</td><td>26.07 %</td><td>73.93%</td></tr>
<tr><td rowspan="4">7c (CPP-A-TPA, CPP as donor)</td><td>S_1</td><td>50.27 %</td><td>31.35 %</td><td>39.70 %</td><td>18.92 %</td><td>81.08%</td></tr>
<tr><td>S_2</td><td>17.58 %</td><td>11.80 %</td><td>14.40 %</td><td>5.79 %</td><td>94.21%</td></tr>
<tr><td>S_3</td><td>9.98 %</td><td>5.53 %</td><td>7.43 %</td><td>4.45 %</td><td>95.55%</td></tr>
<tr><td>S_4</td><td>77.80 %</td><td>72.38 %</td><td>75.04 %</td><td>5.42 %</td><td>94.58%</td></tr>
<tr><td rowspan="4">7c (CPP-A-TPA, TPA as donor)</td><td>S_1</td><td>90.85 %</td><td>96.36 %</td><td>93.56 %</td><td>5.51 %</td><td>94.49%</td></tr>
<tr><td>S_2</td><td>55.75 %</td><td>86.94 %</td><td>69.62 %</td><td>31.19 %</td><td>68.81%</td></tr>
<tr><td>S_3</td><td>98.96 %</td><td>98.80 %</td><td>98.88 %</td><td>0.16 %</td><td>99.84%</td></tr>
<tr><td>S_4</td><td>97.25 %</td><td>99.59 %</td><td>98.42 %</td><td>2.34 %</td><td>97.66%</td></tr>
<tr><td rowspan="4">7c (CPP-A-TPA, CPP and TPA as donors)</td><td>S_1</td><td>59.42 %</td><td>34.99 %</td><td>45.60 %</td><td>24.43 %</td><td>75.57%</td></tr>
<tr><td>S_2</td><td>61.83 %</td><td>24.85 %</td><td>39.20 %</td><td>36.98 %</td><td>63.02%</td></tr>
<tr><td>S_3</td><td>11.02 %</td><td>6.73 %</td><td>8.61 %</td><td>4.29 %</td><td>95.71%</td></tr>
<tr><td>S_4</td><td>80.55 %</td><td>72.79 %</td><td>76.57 %</td><td>7.77 %</td><td>92.23%</td></tr>
</table>

[a]Contribution percentage of CPP unit to the hole distribution of hole-electron analysis; [b]Contribution percentage of CPP unit to the electron distribution of hole-electron analysis; [c]Hole-electron overlap percentage of hole-electron analysis; [d]Contribution percentage of CT character for specific states; [e]Contribution percentage of LE character for specific states, LE% = 1 – CT%.

6.5 Natural transition orbital (NTO) analysis

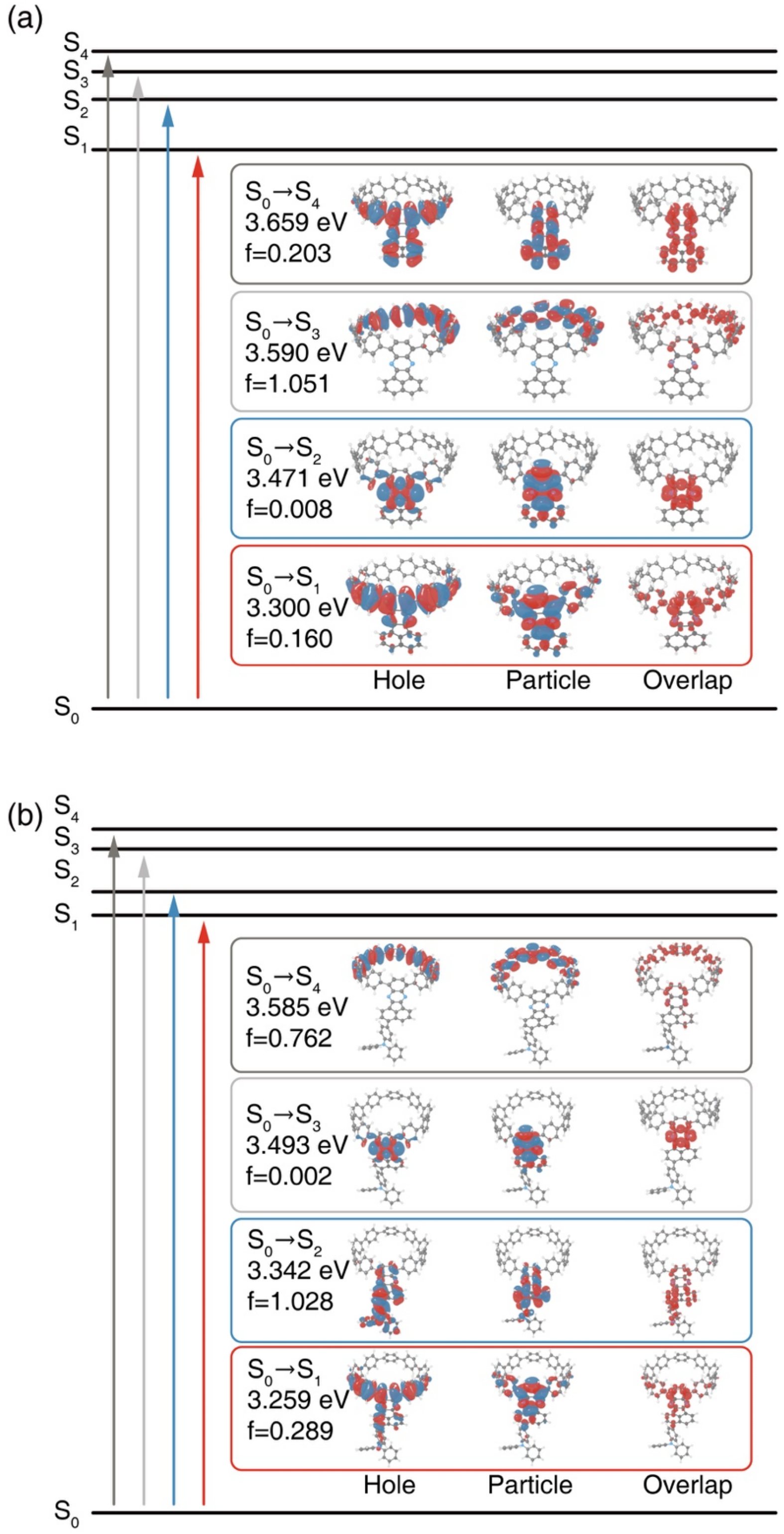

Figure S20. Natural transition orbital (NTO) analysis of **7a** (a) and **7c** (b) calculated by TD-DFT (M06-2X/6-311G**, PCM = toluene).

7. Ultrafast transient absorption measurements

7.1 General

A commercial Yb:KGW laser system (33 kHz, ~180 fs, 1030 nm) was employed as main laser source for the transient absorption measurements. The excitation pulses at 330 nm were generated from the second-harmonic output of a collinear optical parametric amplifier, while the probe pulses were produced from a static sapphire window, the probe spectrum extends from 460 nm to 850 nm. Pump and probe beams were spatially overlapped in a quartz cuvette with 500 μm sample thickness, the sample optical density was controlled to 0.1 – 0.3 at 330 nm. The polarizations of the pump and probe beams were set to the magic angle of 54.7°. After passing the sample, the probe pulses were dispersed in a grating spectrometer and detected by a linear Si detector array. The measured TA data were evaluated via target analysis with the software package Glotaran based on the R-package TIMP.[18,19]

7.2 Comparison of observed ESA band and reported one

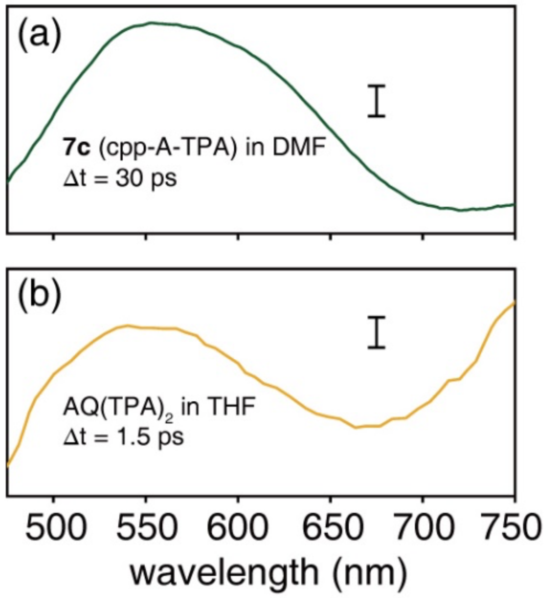

Figure S21. Comparison of TA spectra. (a) Observed TA spectra of **7c** in DMF at 30 ps delay; (b) reported TA spectra of $AQ(TPA)_2$ in THF at 1.5 ps delay.[20]

7.3 Target analysis of TA data

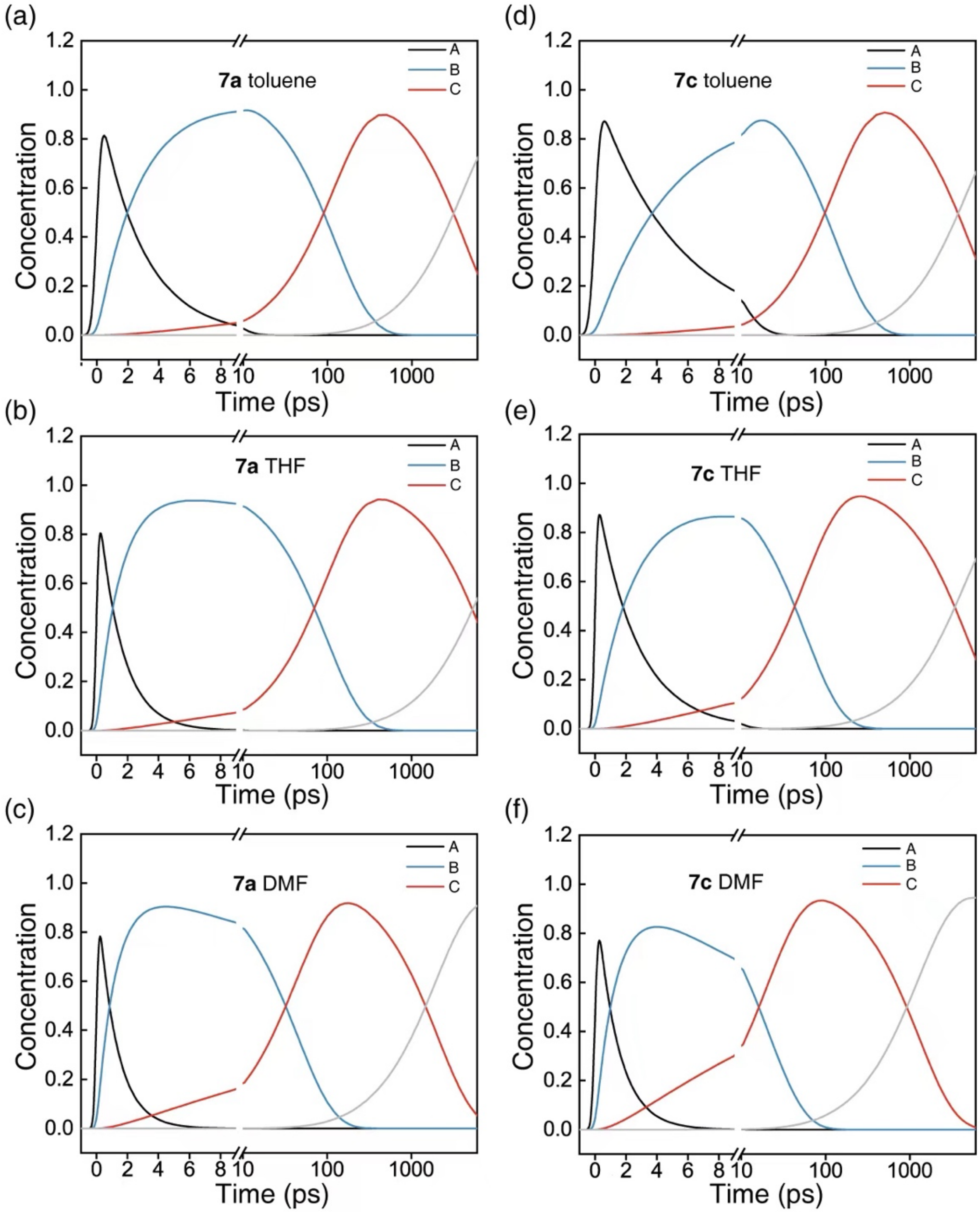

Figure S22. Concentration evolution of transient species of **7a** (a-c) and **7c** (d-f) in toluene, THF and DMF solutions.

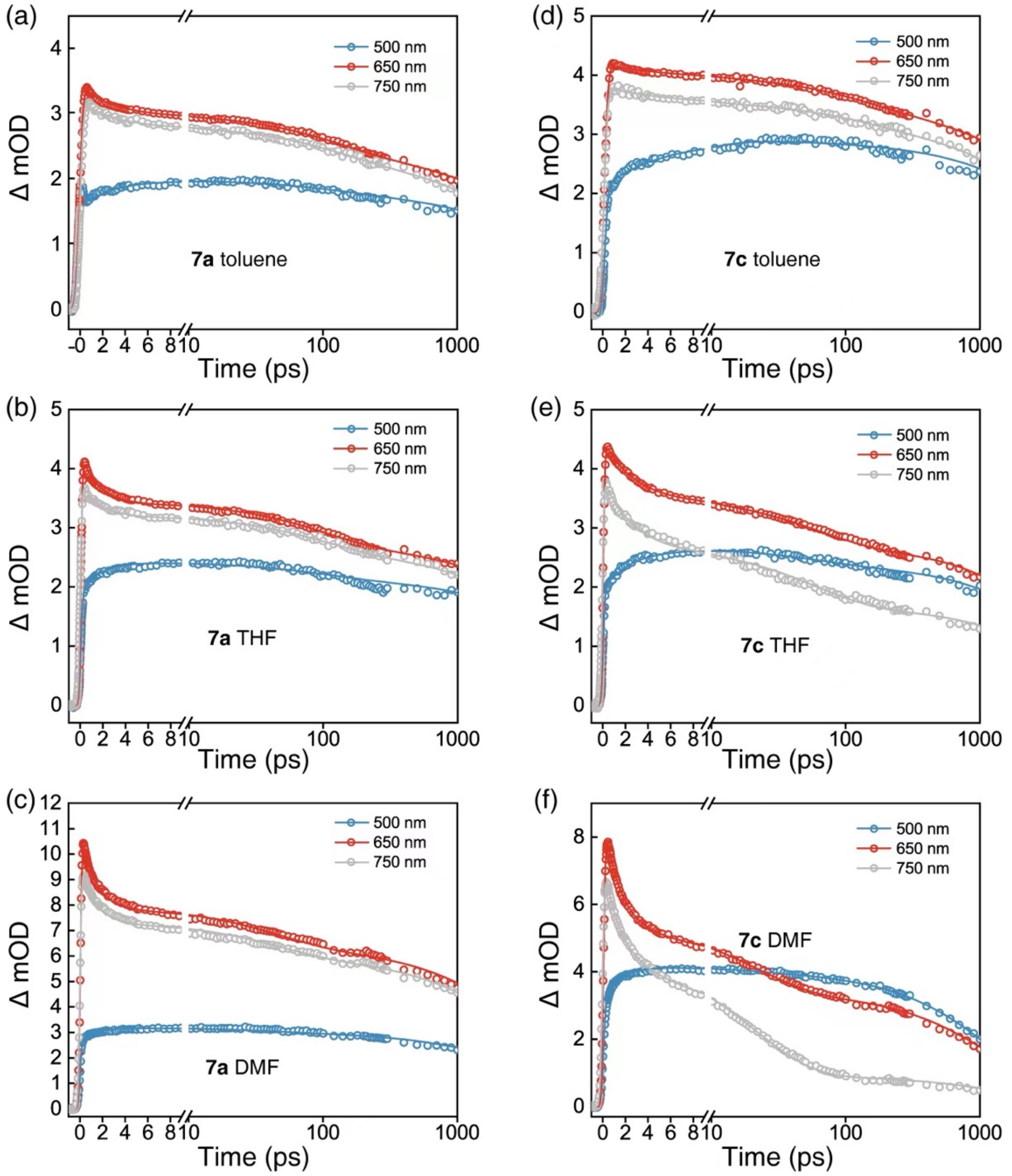

Figure S23. Time traces at selected probe wavelength of fs-TA of **7a** (a-c) and **7c** (d-f) in toluene, THF and DMF solutions.

8. $^1H/^{13}C$ NMR and MS spectra for new compounds

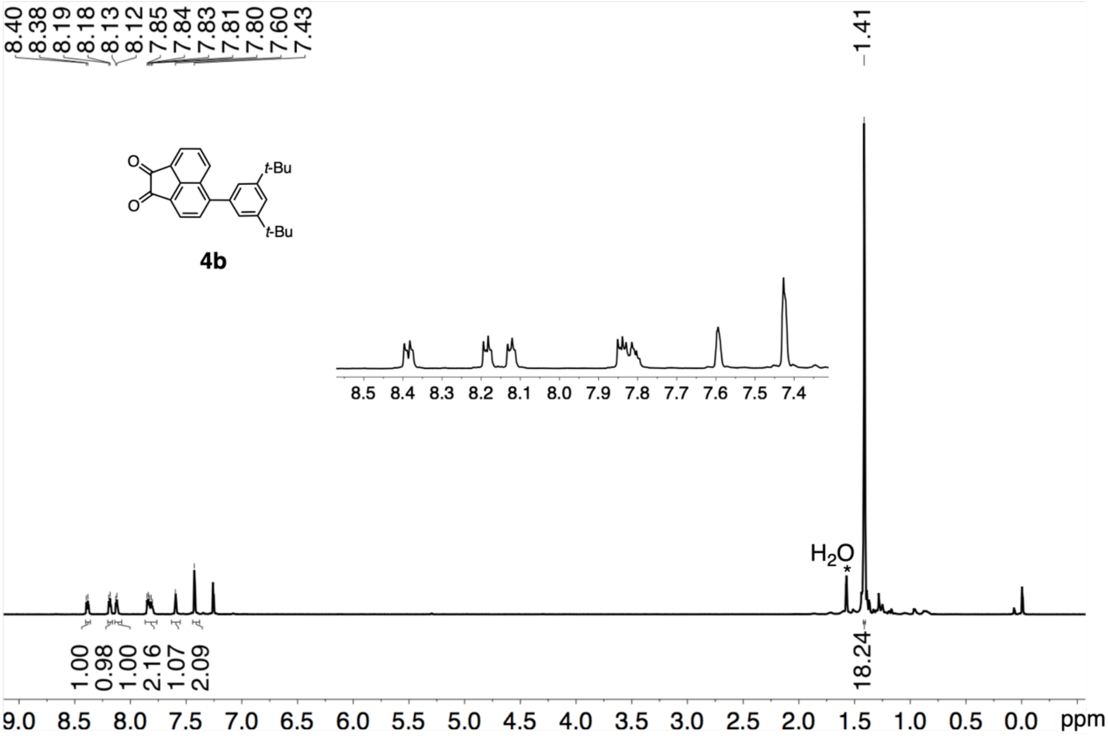

Figure S24. ^{1}H NMR spectrum of compound **4b** in $CDCl_3$ (293 K, 600 MHz). Asterisk indicates signal of solvents.

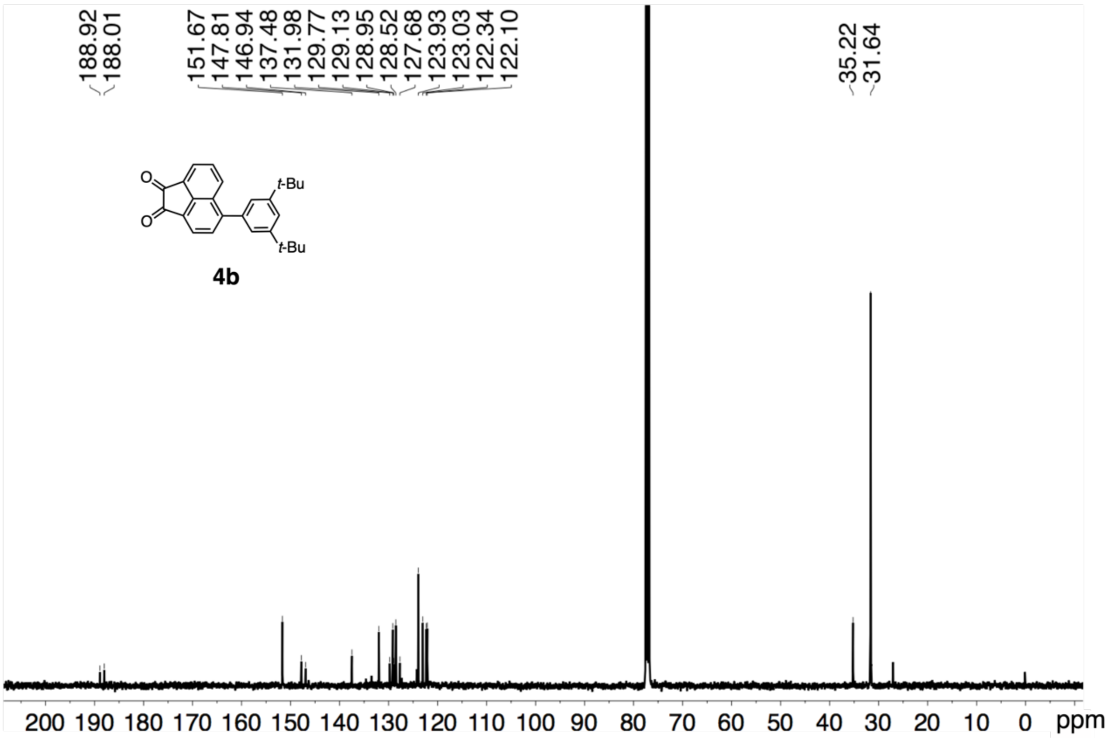

Figure S25. ^{13}C NMR spectrum of compound **4b** in $CDCl_3$ (293 K, 150 MHz).

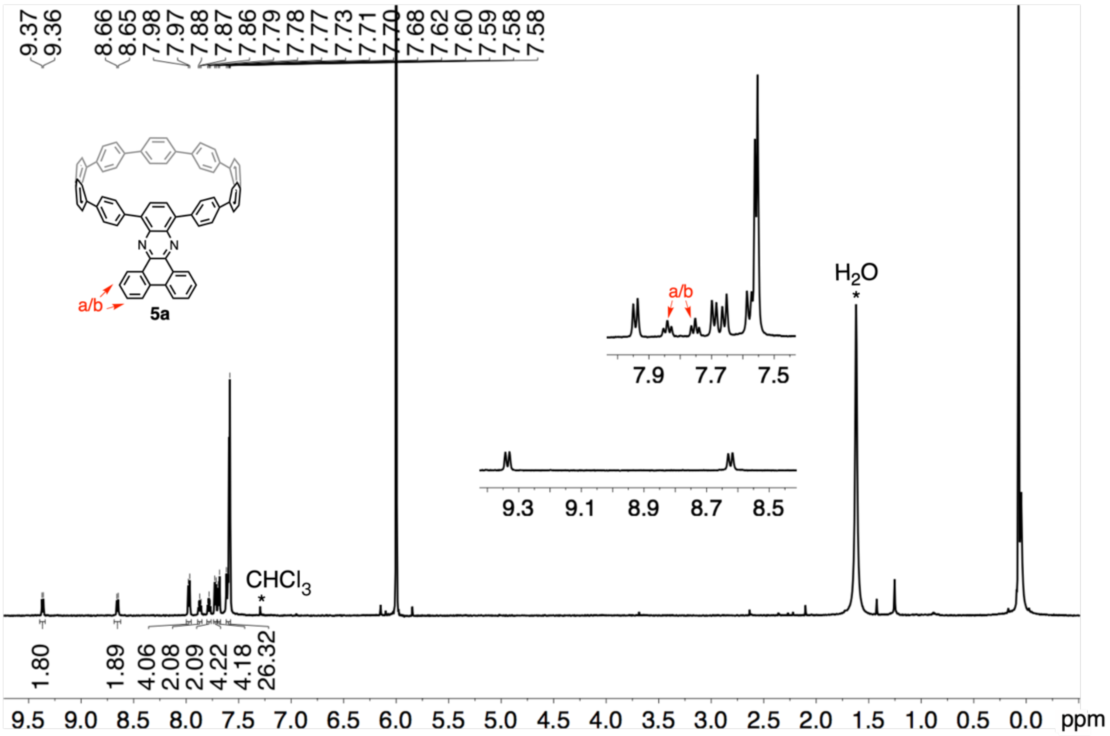

Figure S26. ^{1}H NMR spectrum of compound **5a** in 1,1,2,2-$C_2D_2Cl_4$ (293 K, 600 MHz). Asterisk indicates signal of solvents.

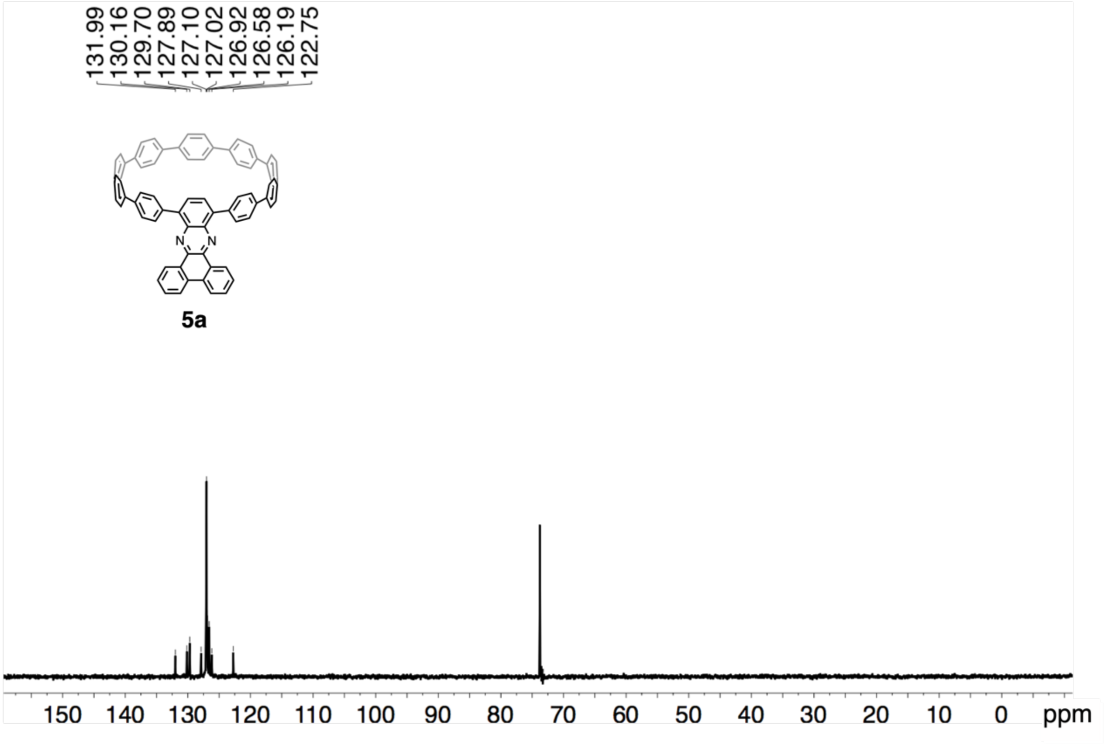

Figure S27. DEPT 135 ^{13}C NMR spectrum of compound **5a** in 1,1,2,2-$C_2D_2Cl_4$ (293 K, 150 MHz).

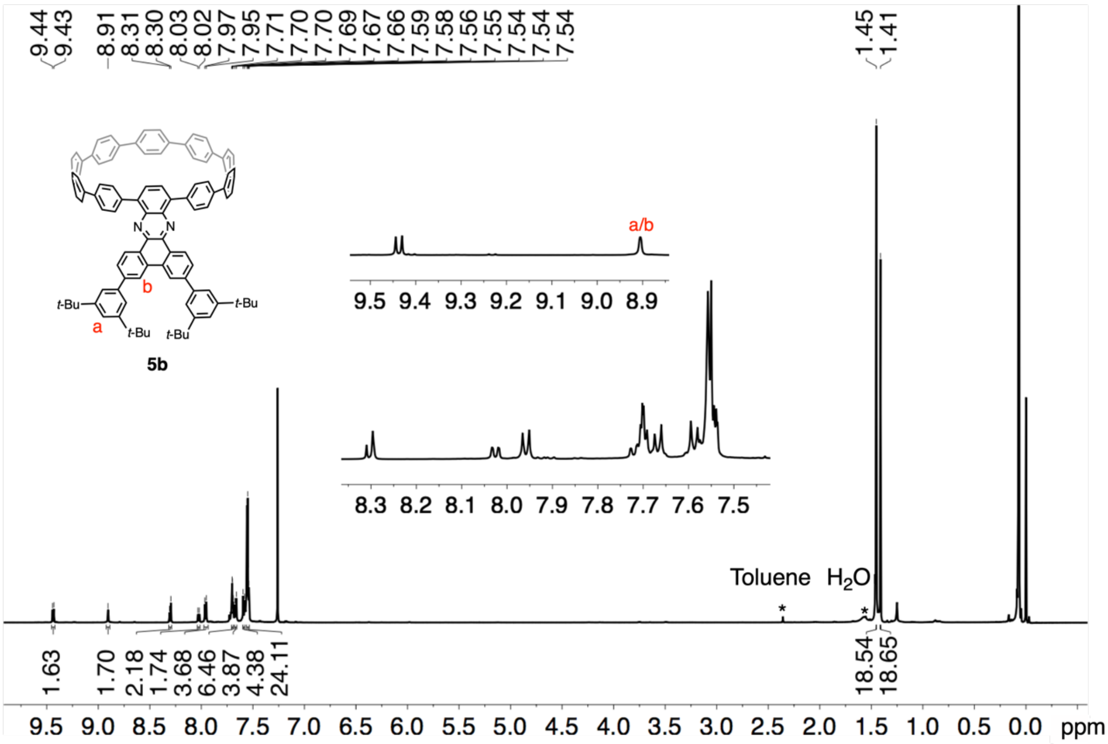

Figure S28. ^{1}H NMR spectrum of compound **5b** in $CDCl_3$ (293 K, 600 MHz). Asterisk indicates signal of solvents.

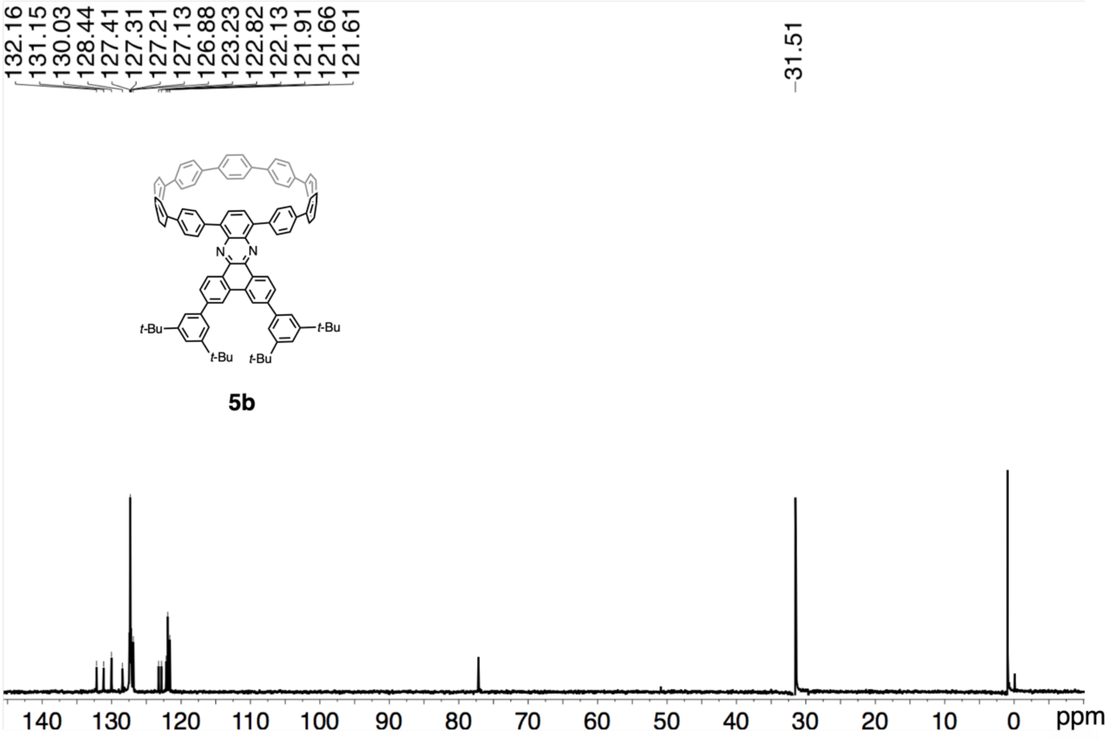

Figure S29. DEPT 135 ^{13}C NMR spectrum of compound **5b** in $CDCl_3$ (293 K, 150 MHz).

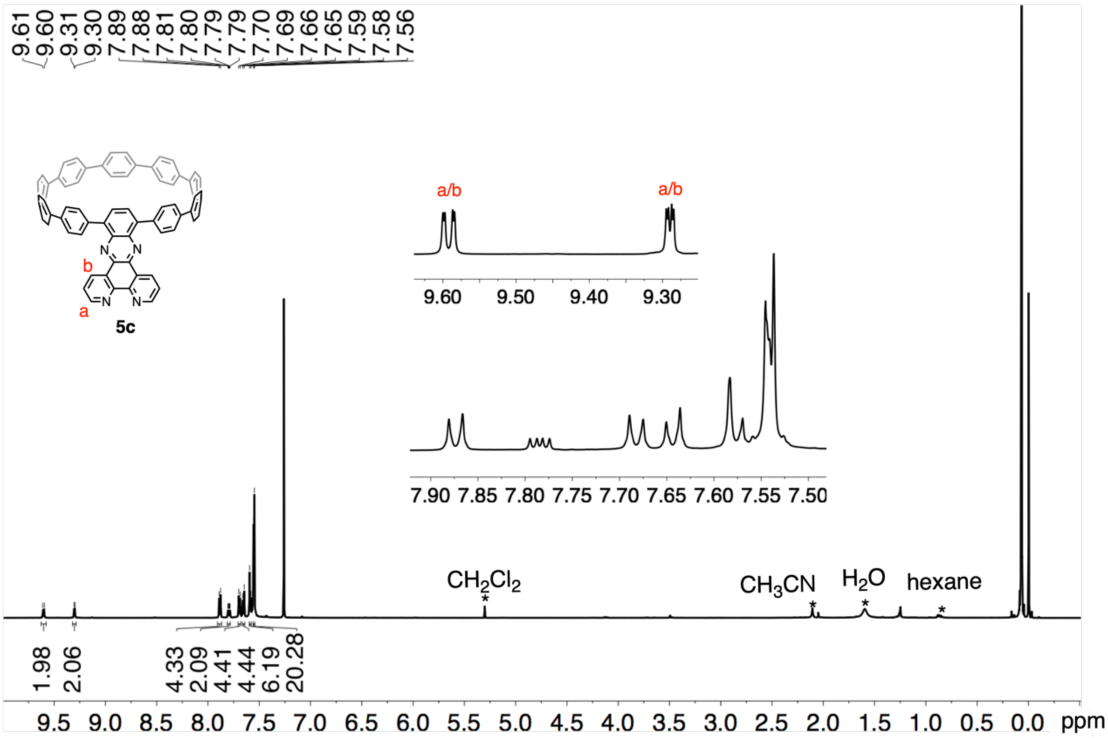

Figure S30. ^{1}H NMR spectrum of compound **5c** in $CDCl_3$ (293 K, 600 MHz). Asterisk indicates signal of solvents.

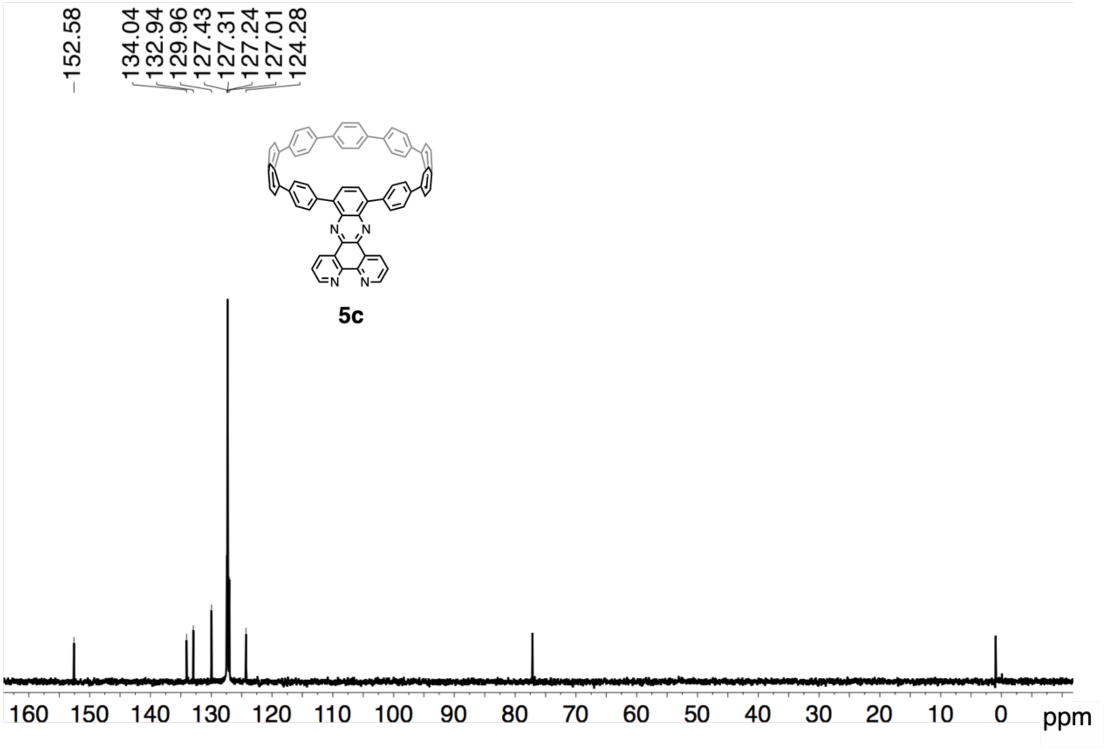

Figure S31. DEPT 135 ^{13}C NMR spectrum of compound **5c** in $CDCl_3$ (293 K, 150 MHz).

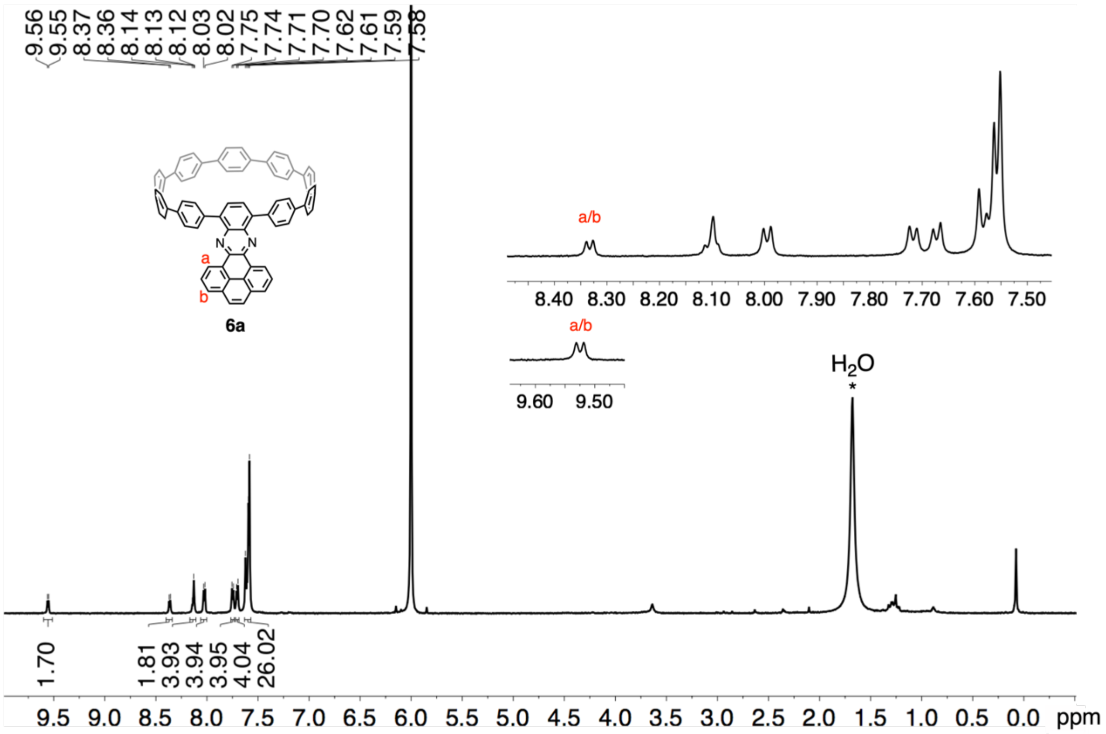

Figure S32. ^{1}H NMR spectrum of compound **6a** in 1,1,2,2-$C_2D_2Cl_4$ (293 K, 600 MHz). Asterisk indicates signal of solvents.

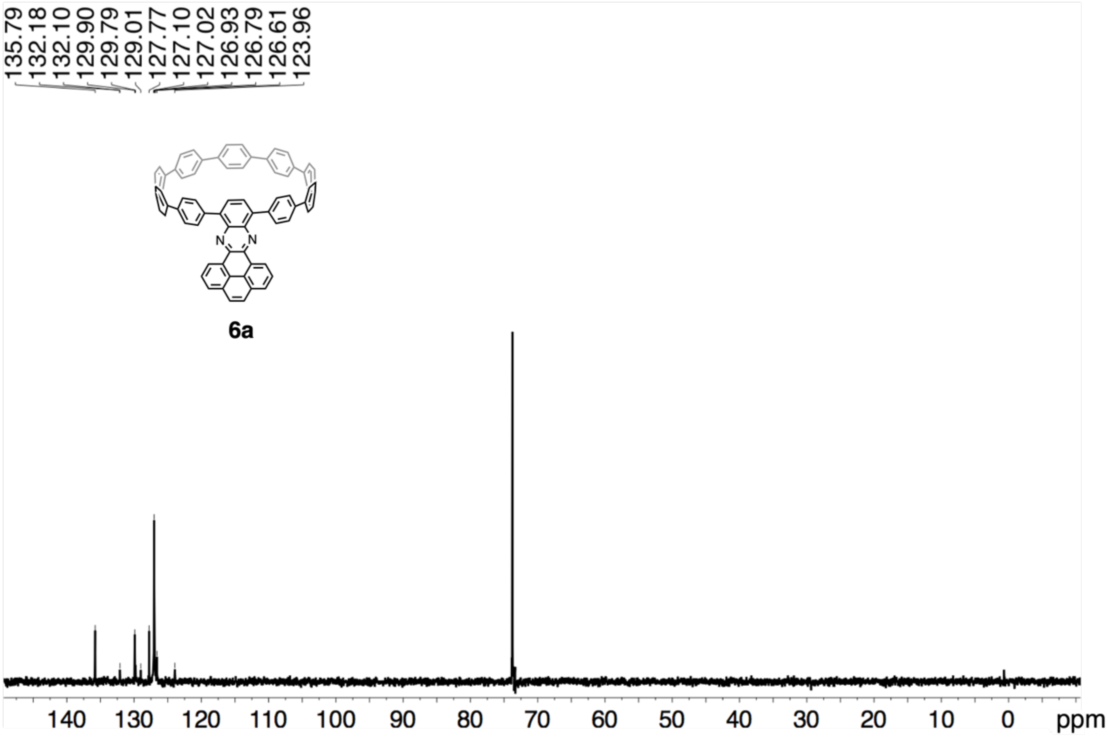

Figure S33. DEPT 135 ^{13}C NMR spectrum of compound **6a** in 1,1,2,2-$C_2D_2Cl_4$ (293 K, 150 MHz).

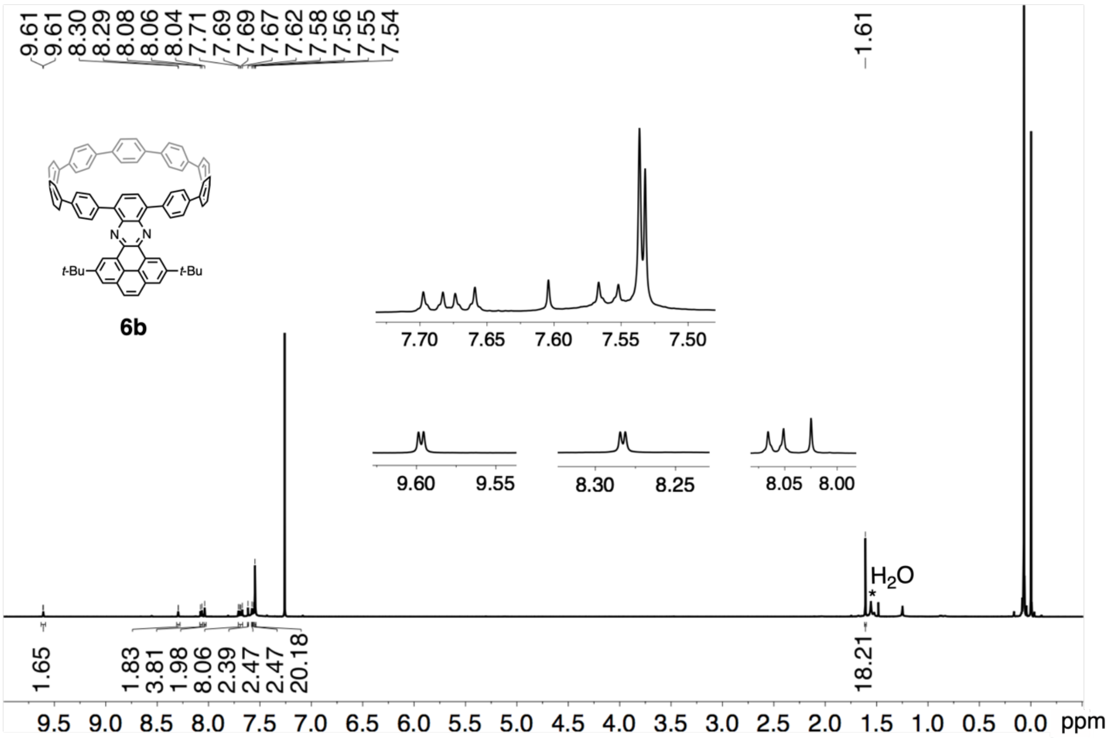

Figure S34. ^{1}H NMR spectrum of compound **6b** in $CDCl_3$ (293 K, 600 MHz). Asterisk indicates signal of solvents.

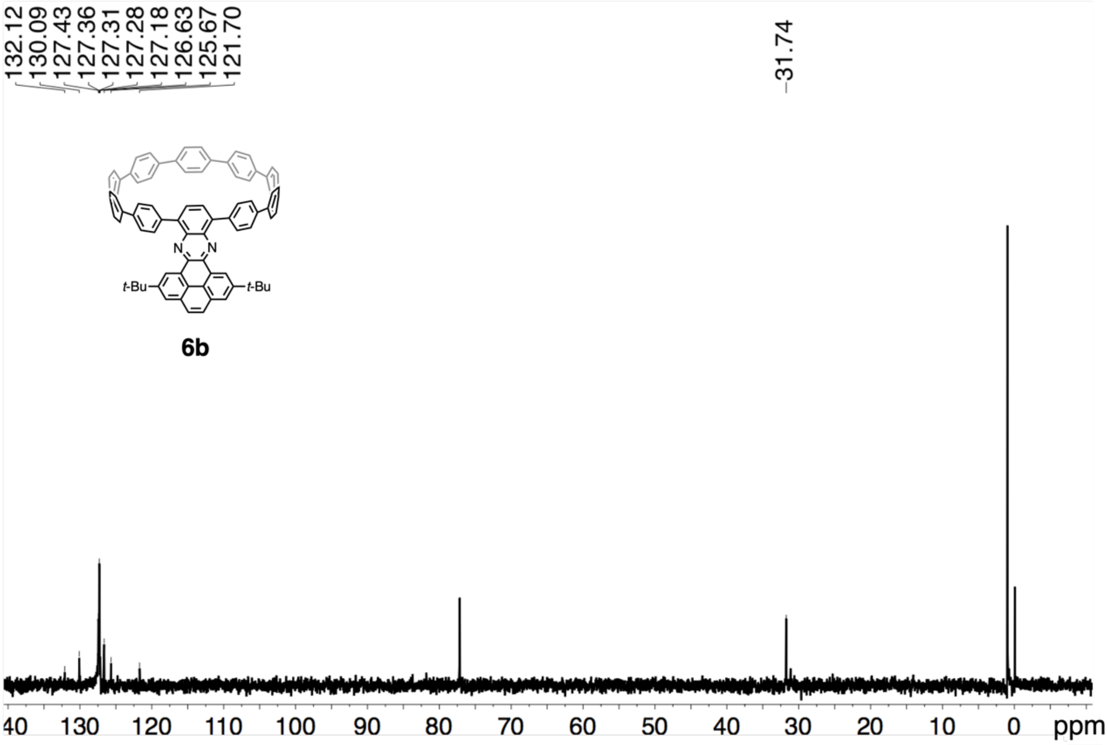

Figure S35. DEPT 135 ^{13}C NMR spectrum of compound **6b** in $CDCl_3$ (293 K, 150 MHz).

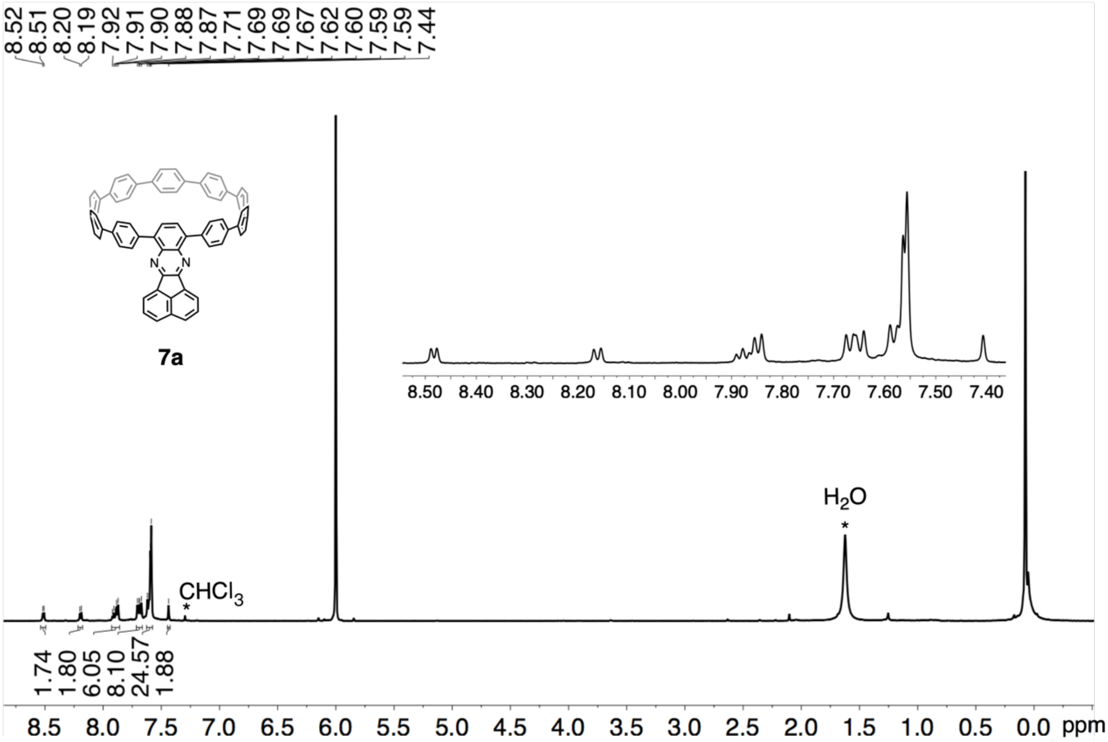

Figure S36. ^{1}H NMR spectrum of compound **7a** in 1,1,2,2-$C_2D_2Cl_4$ (293 K, 600 MHz). Asterisk indicates signal of solvents.

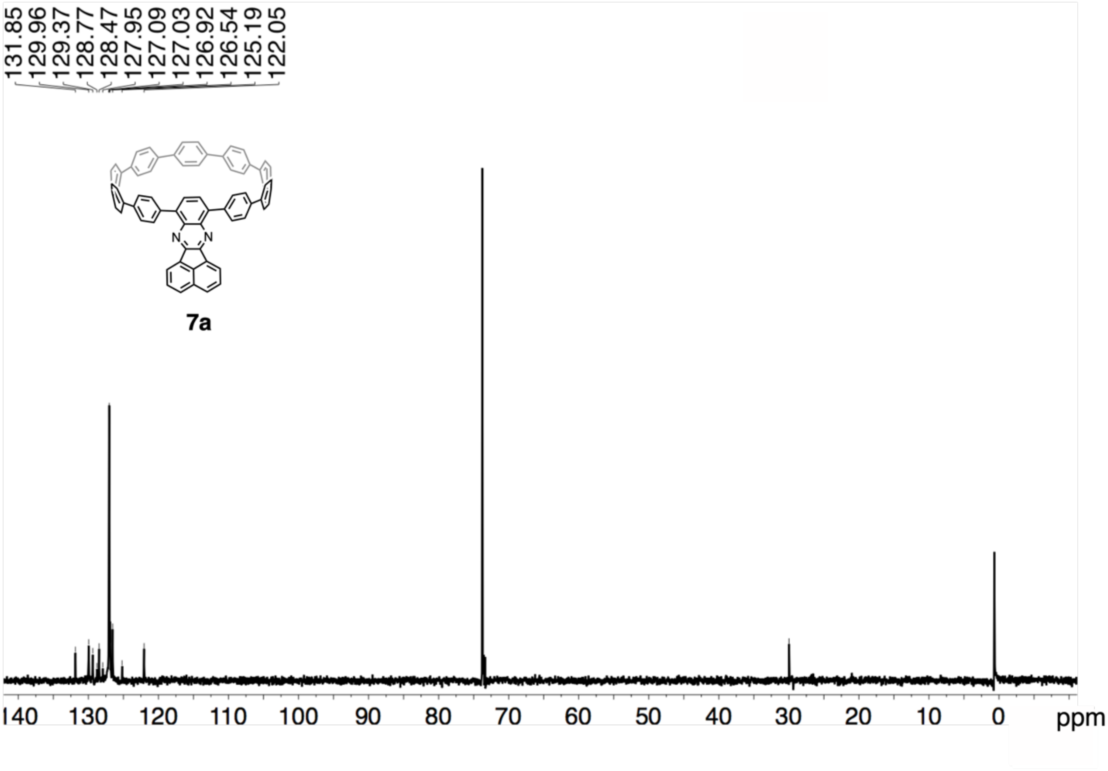

Figure S37. DEPT 135 ^{13}C NMR spectrum of compound **7a** in 1,1,2,2-$C_2D_2Cl_4$ (293 K, 150 MHz).

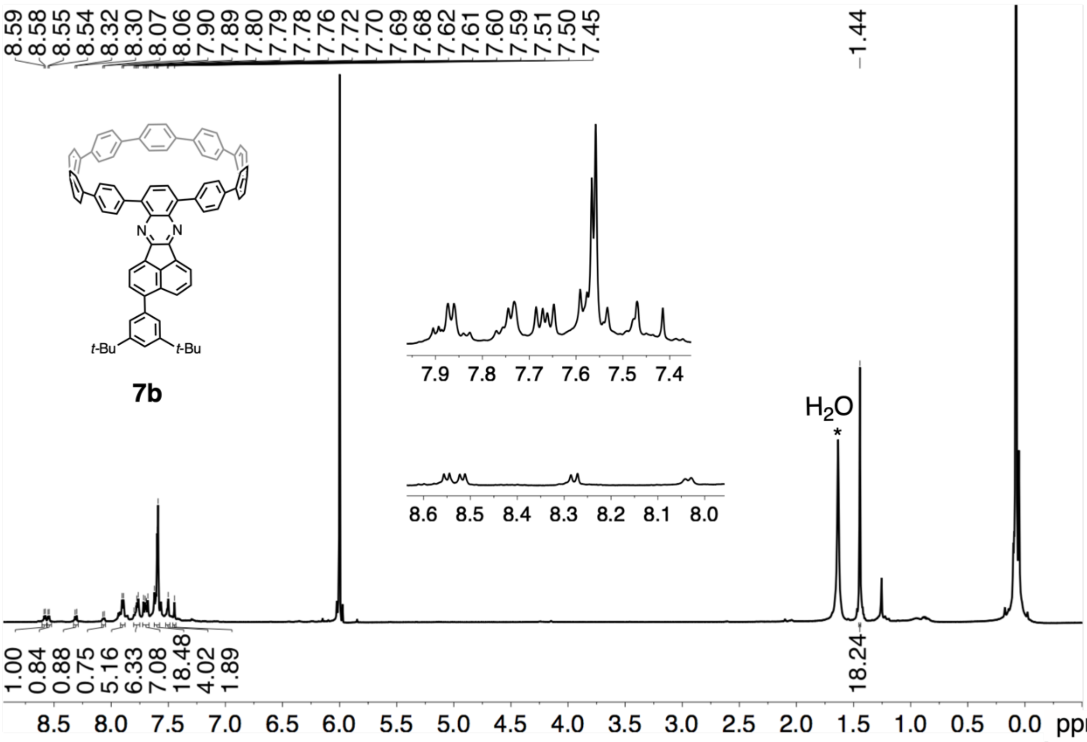

Figure S38. ^{1}H NMR spectrum of compound **7b** in 1,1,2,2-$C_2D_2Cl_4$ (293 K, 600 MHz). Asterisk indicates signal of solvents.

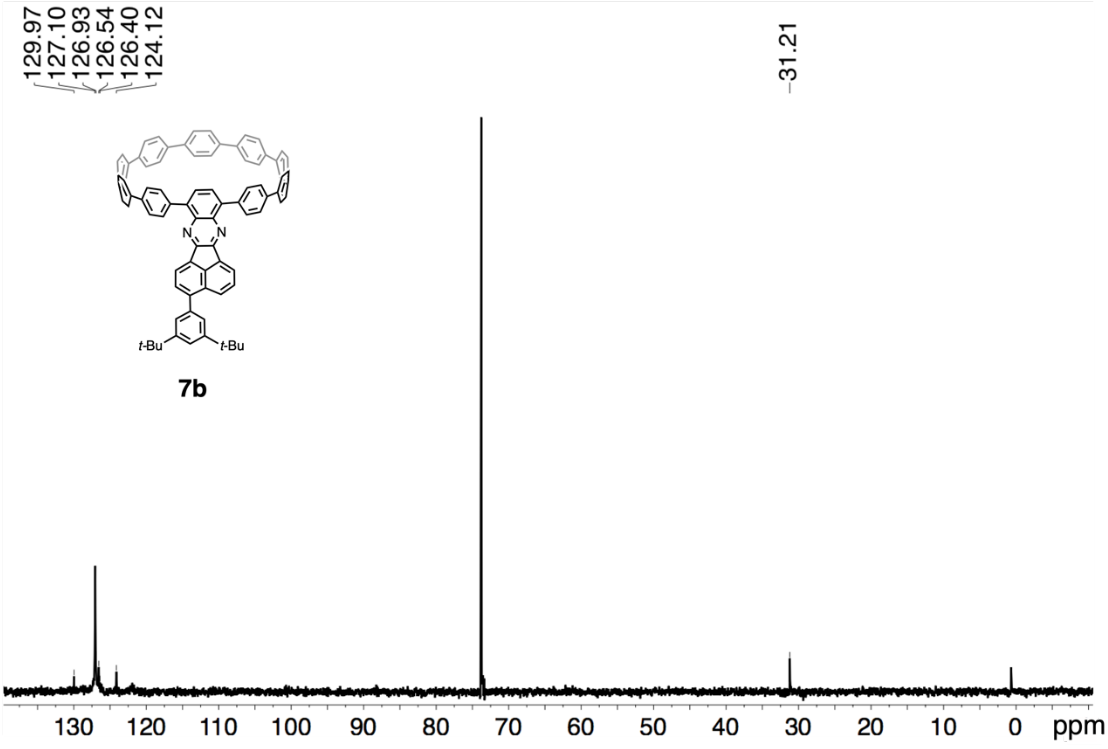

Figure S39. DEPT 135 ^{13}C NMR spectrum of compound **7b** in 1,1,2,2-$C_2D_2Cl_4$ (293 K, 150 MHz).

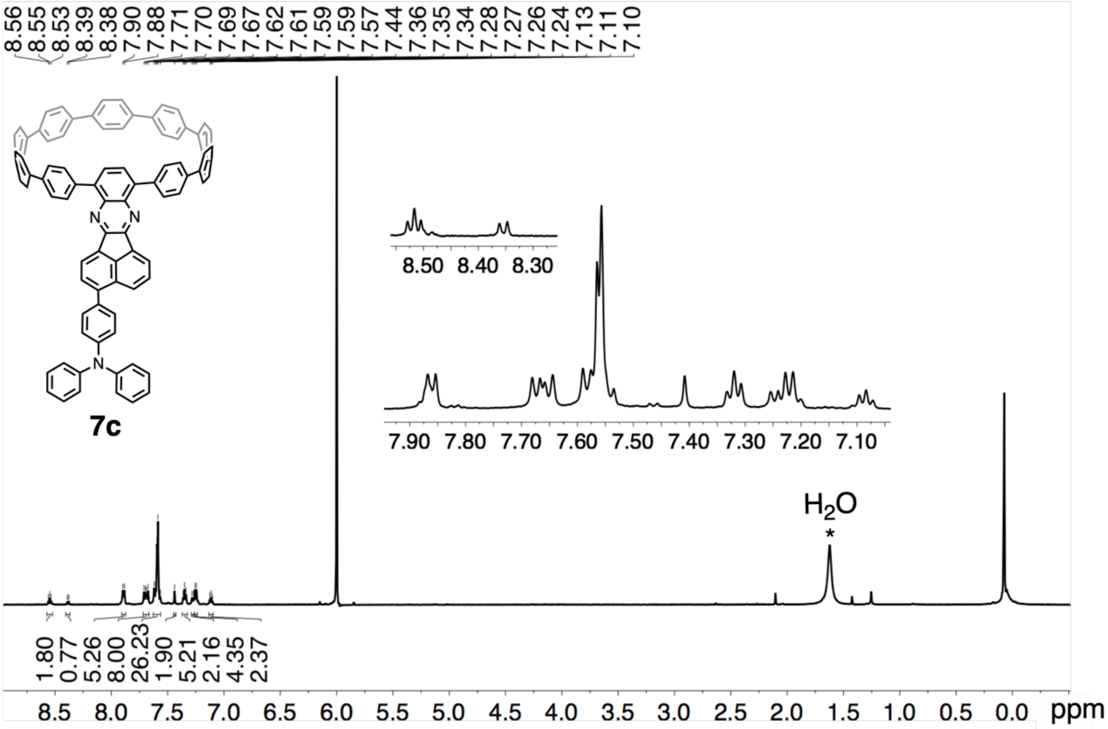

Figure S40. ^{1}H NMR spectrum of compound **7c** in 1,1,2,2-$C_2D_2Cl_4$ (293 K, 600 MHz). Asterisk indicates signal of solvents.

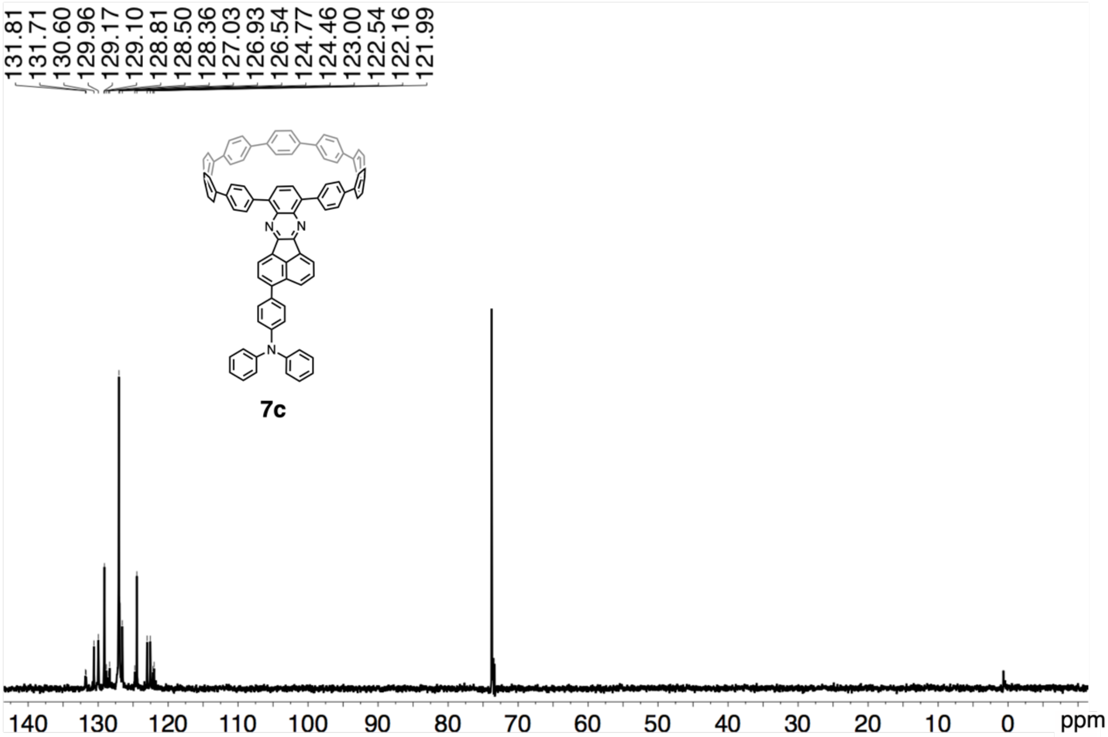

Figure S41. DEPT 135 ^{13}C NMR spectrum of compound **7c** in 1,1,2,2-$C_2D_2Cl_4$ (293 K, 150 MHz).

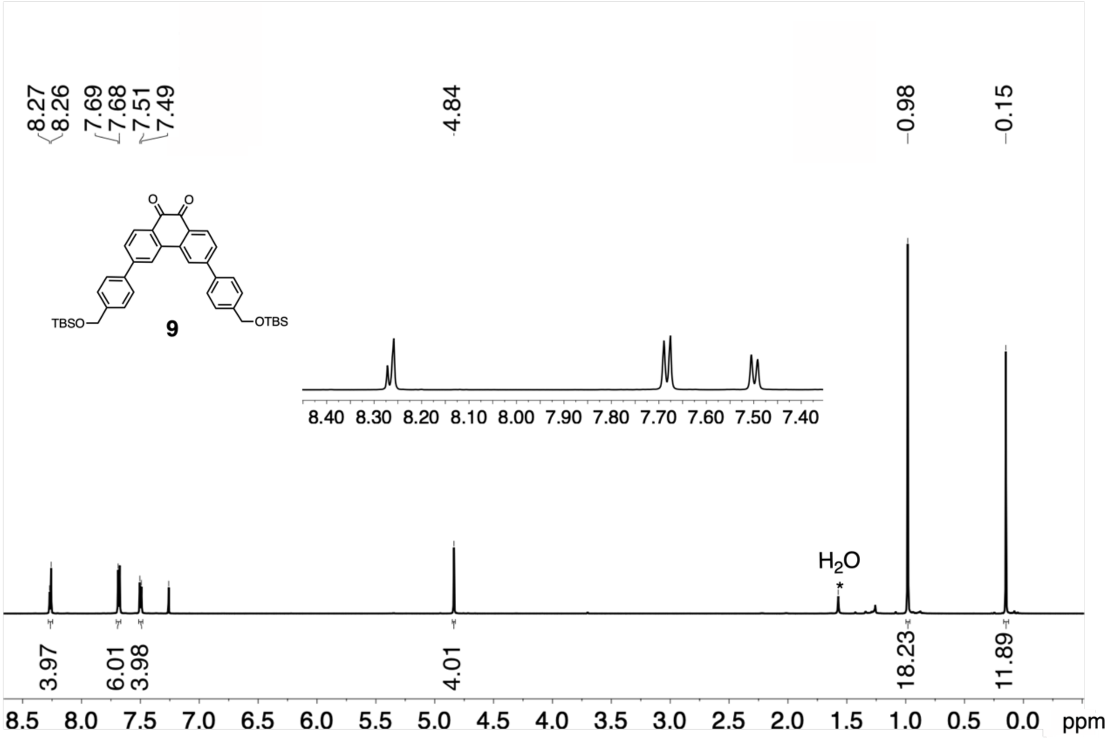

Figure S42. ^{1}H NMR spectrum of compound **9** in $CDCl_3$ (293 K, 600 MHz). Asterisk indicates signal of solvents.

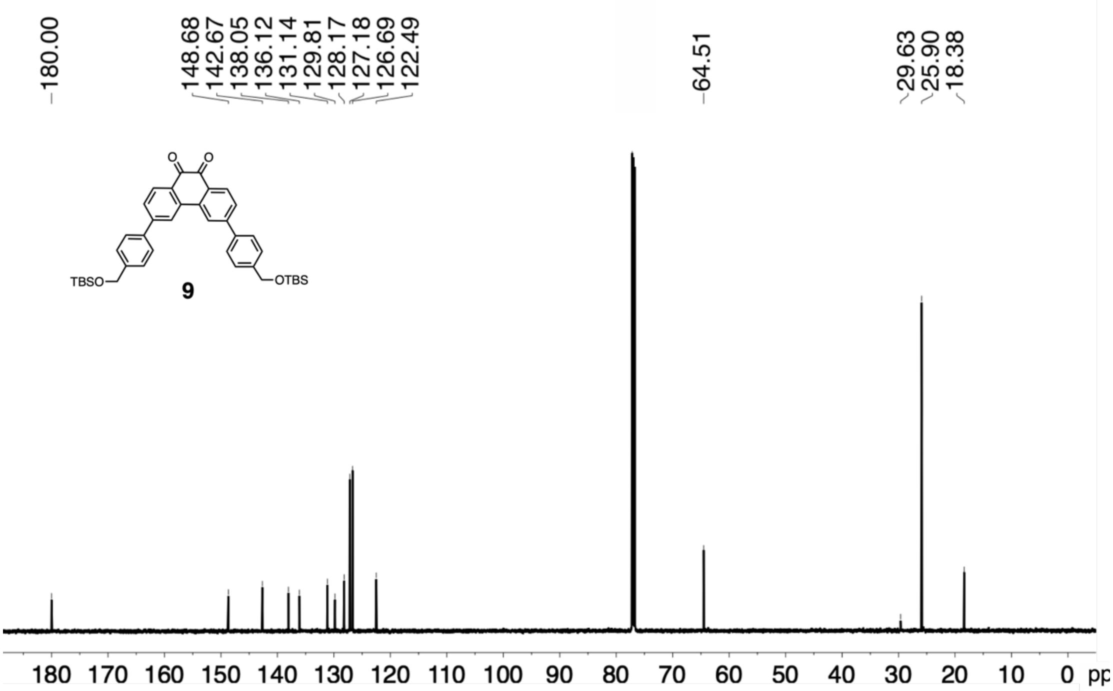

Figure S43. ^{13}C NMR spectrum of compound **9** in $CDCl_3$ (293 K, 150 MHz).

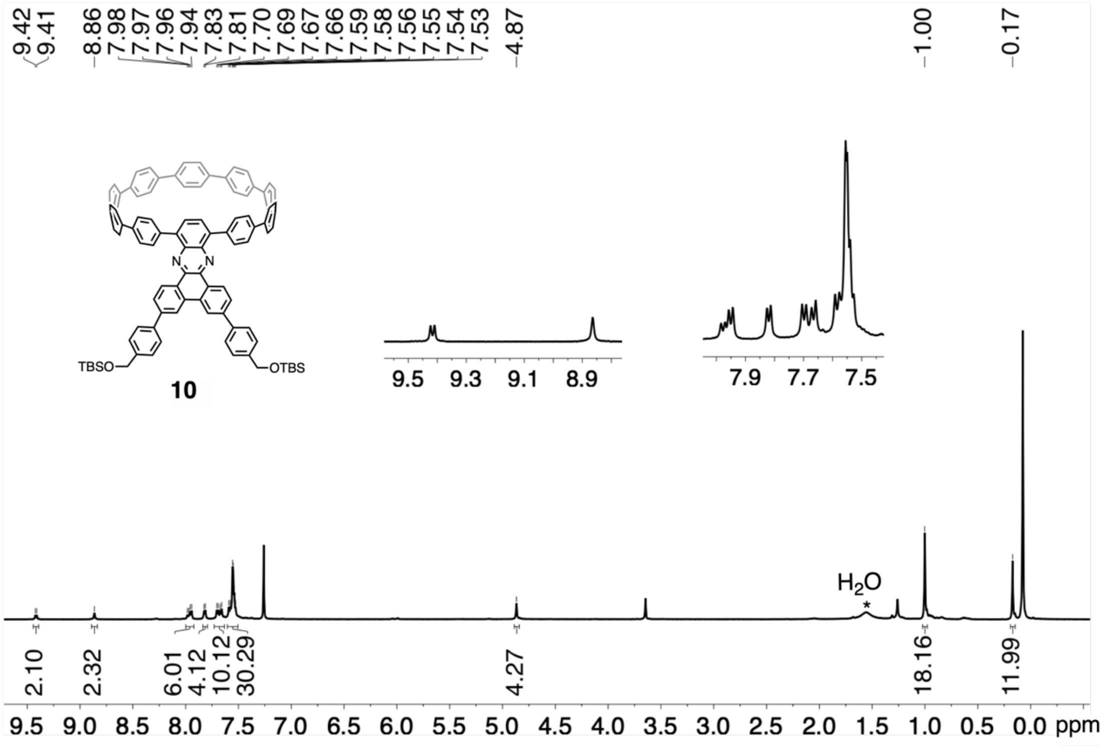

Figure S44. ^{1}H NMR spectrum of compound **10** in $CDCl_3$ (293 K, 400 MHz). Asterisk indicates signal of solvents.

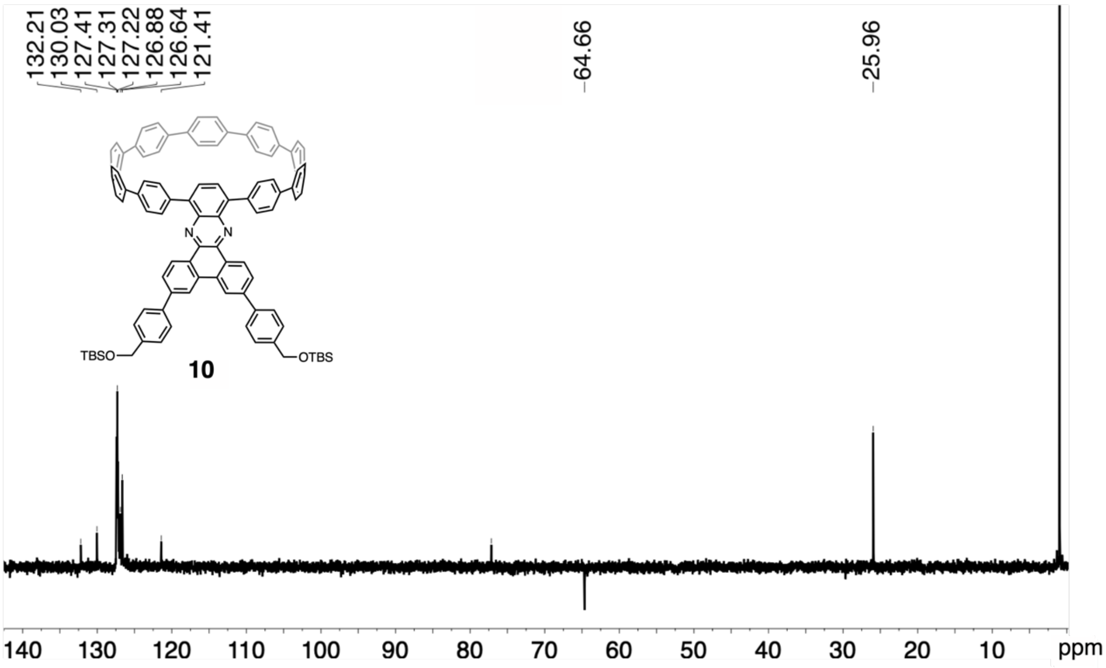

Figure S45. DEPT 135 ^{13}C NMR spectrum of compound **10** in $CDCl_3$ (293 K, 100 MHz).

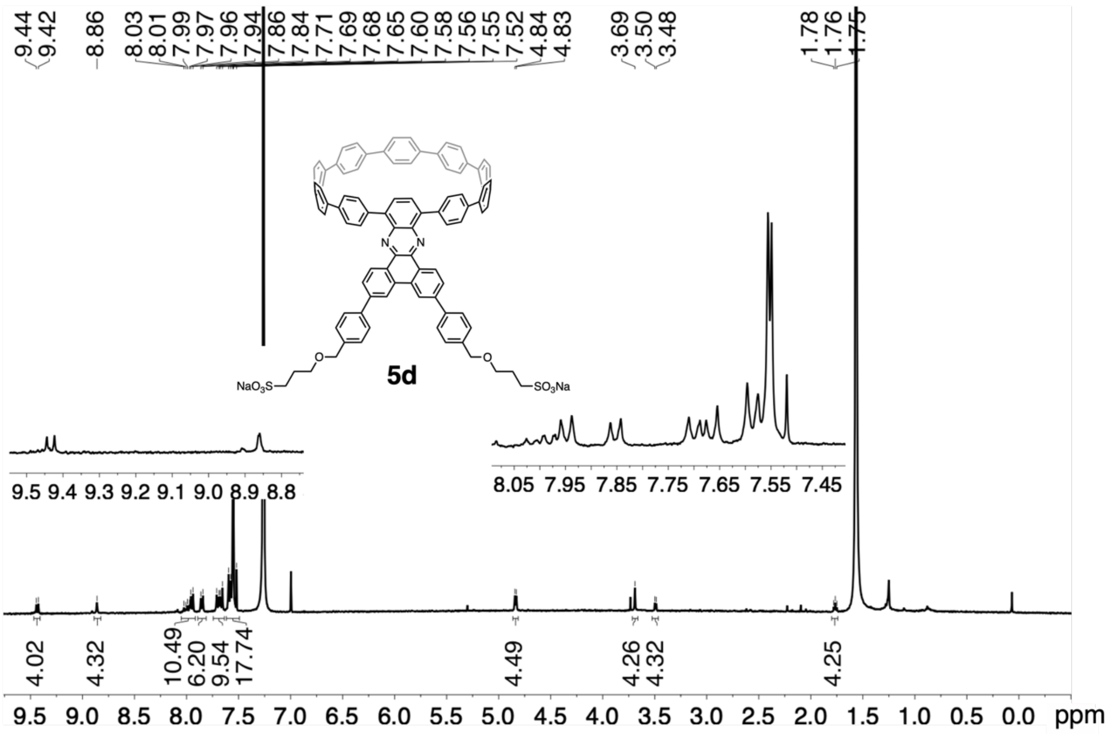

Figure S46. ^{1}H NMR spectrum of compound **5d** in $CDCl_3$ (293 K, 400 MHz).

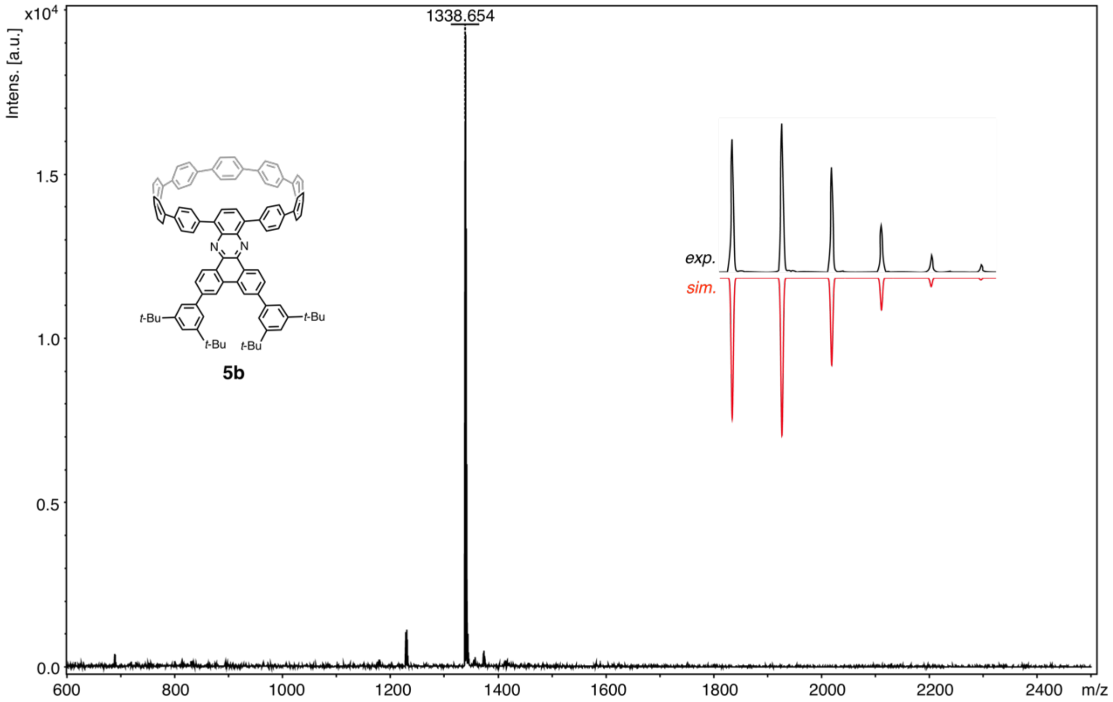

Figure S47. MALDI-TOF MS spectrum of **5b**.

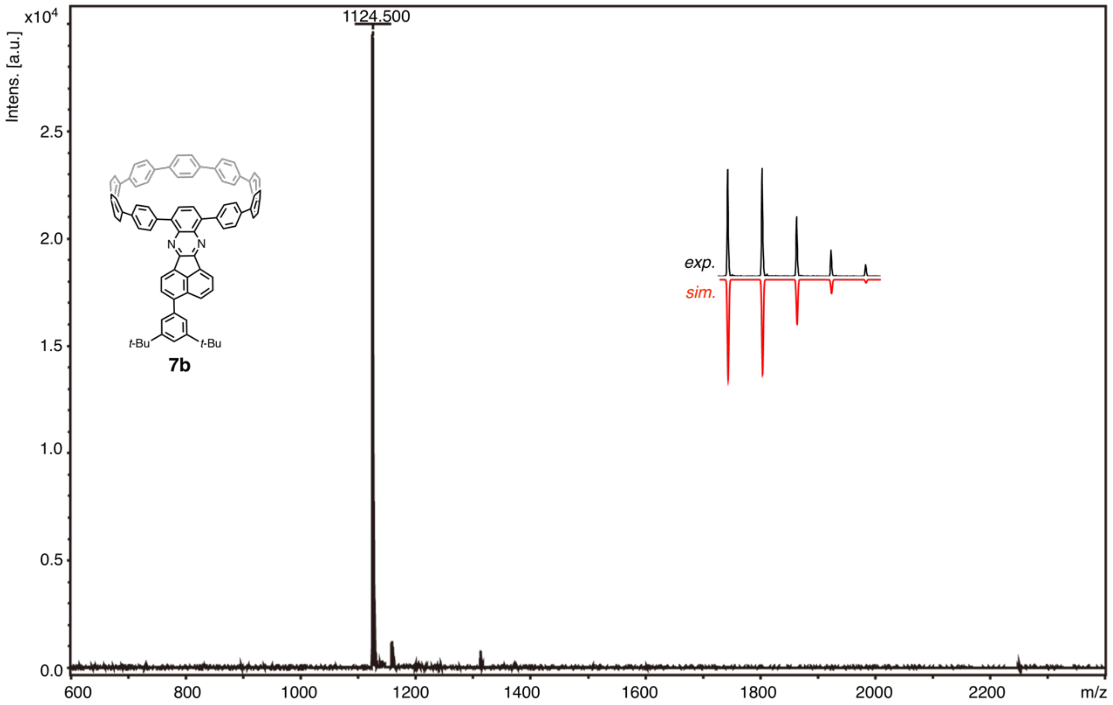

Figure S48. MALDI-TOF MS spectrum of **7b**.

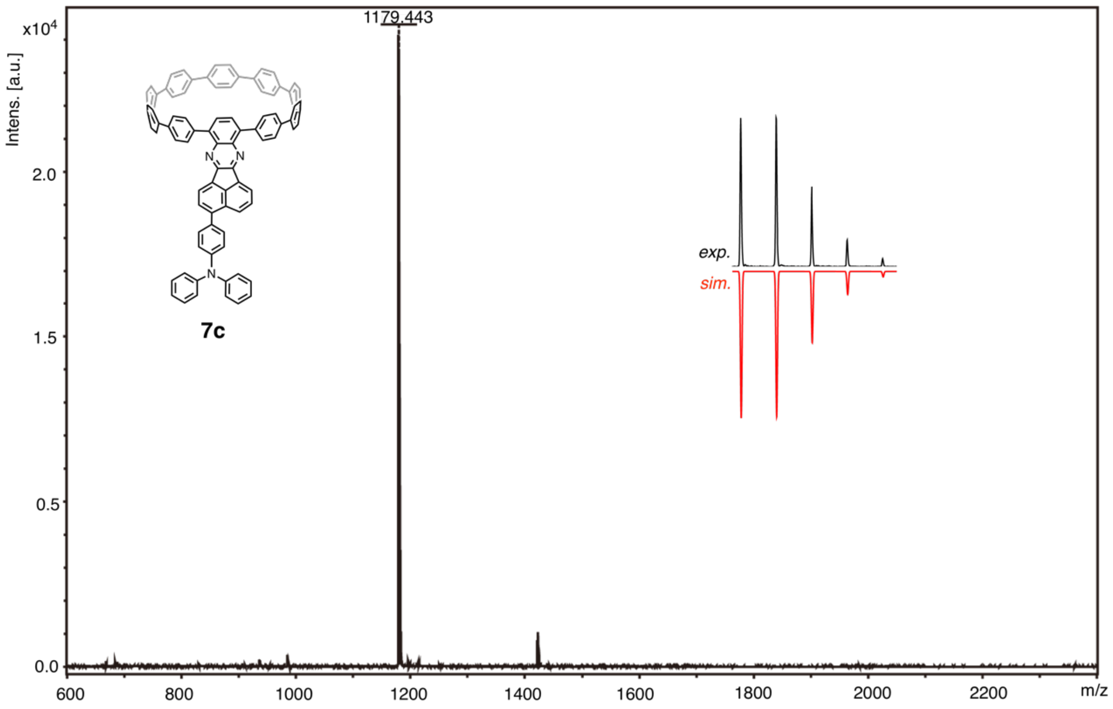

Figure S49. MALDI-TOF MS spectrum of **7c**.

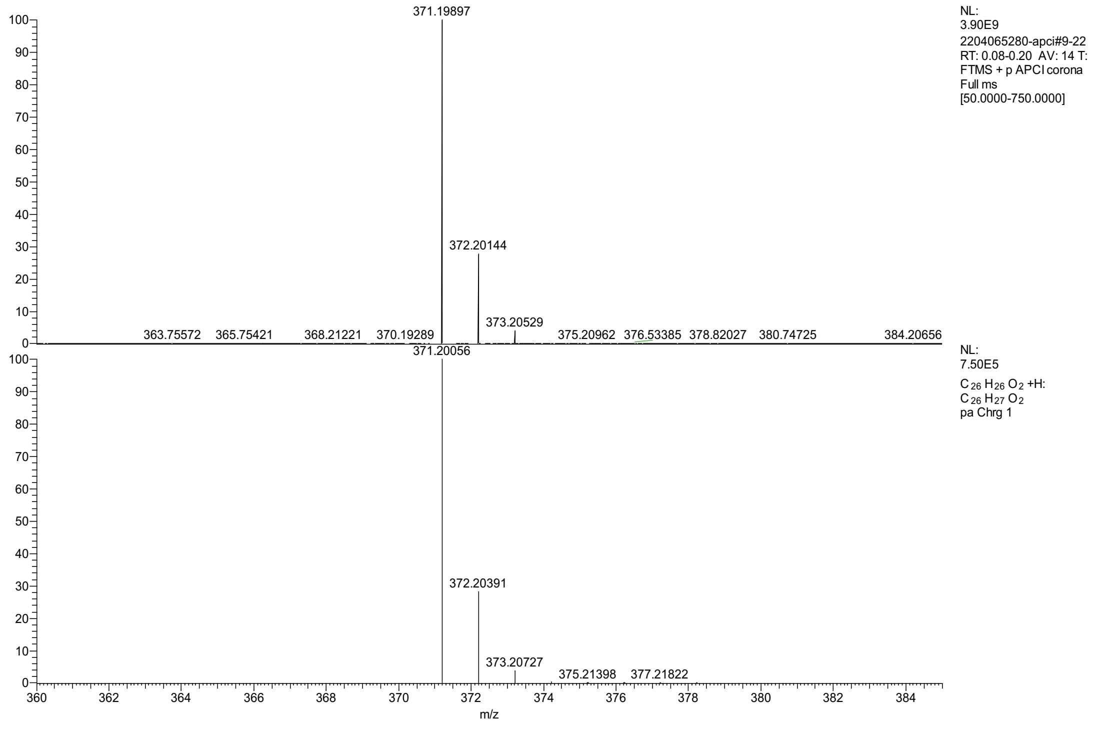

Figure S50. HR-ESI MS spectrum of **4b**.

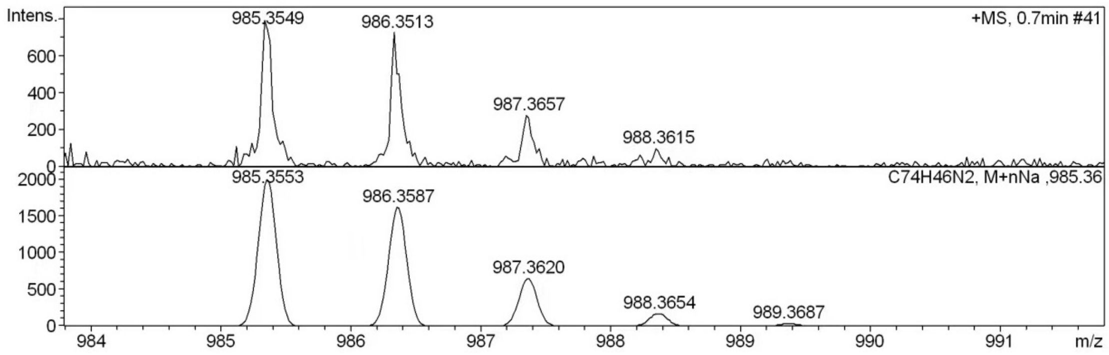

Figure S51. HR-ESI MS spectrum of **5a**.

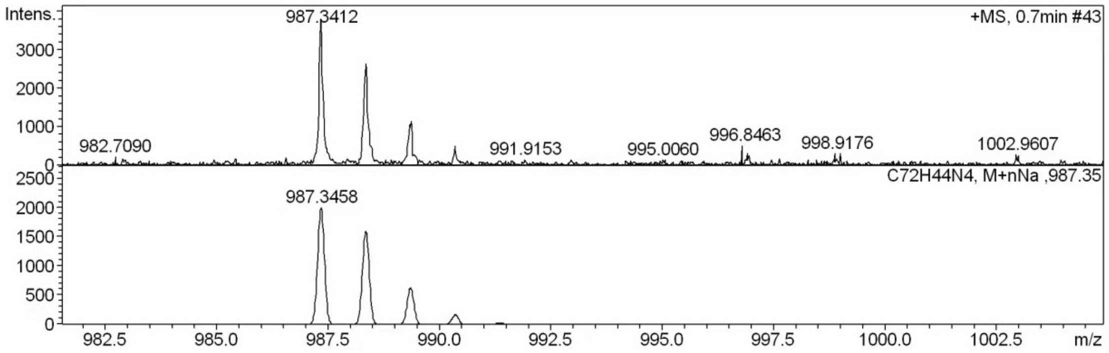

Figure S52. HR-ESI MS spectrum of **5c**.

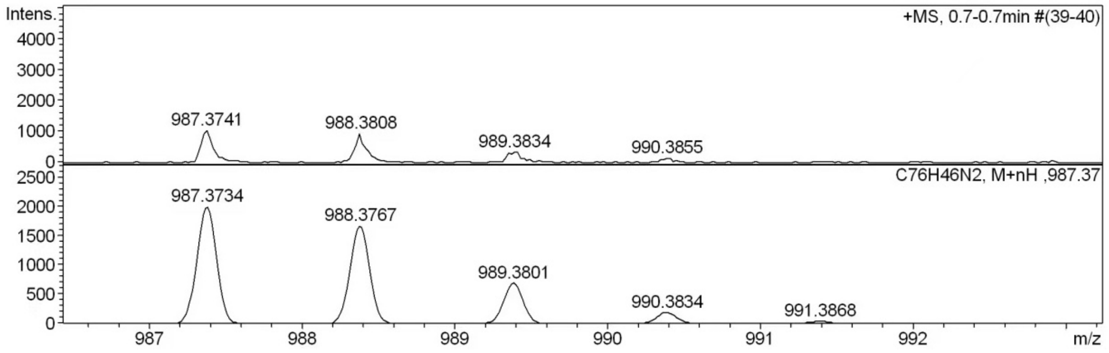

Figure S53. HR-ESI MS spectrum of **6a**.

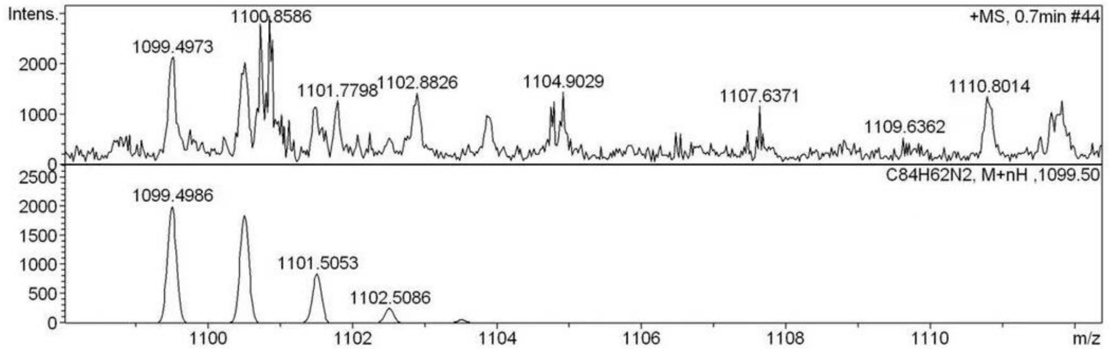

Figure S54. HR-ESI MS spectrum of **6b**.

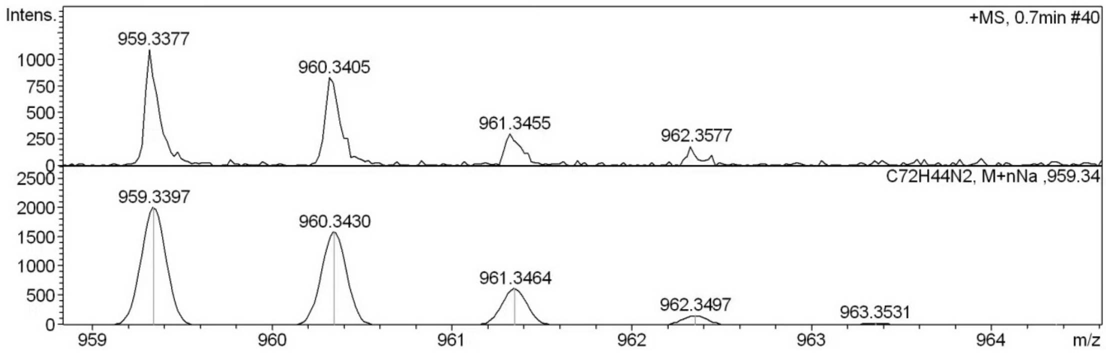

Figure S55. HR-ESI MS spectrum of **7a**.

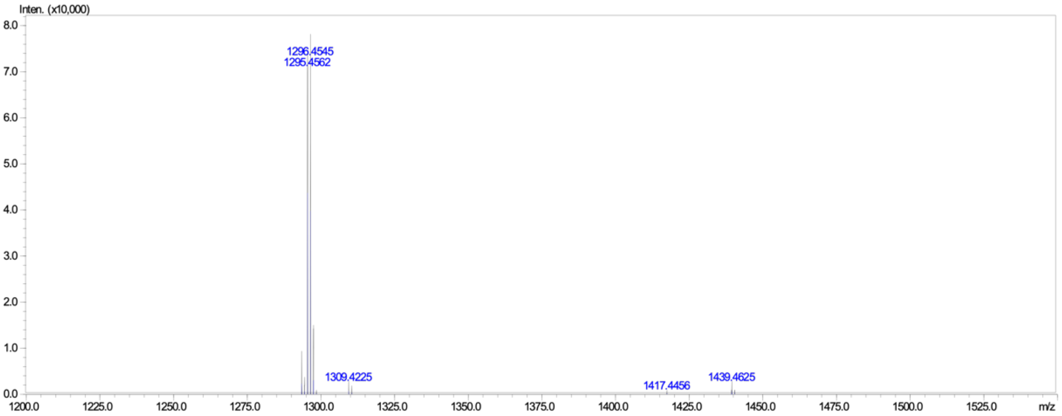

Figure S56. HR-ESI MS spectrum of **5d**.

9. References

1. K. Li, Z. Xu, H. Deng, Z. Zhou, Y. Dang and Z. Sun, *Angew. Chem. Int. Ed*., 2021, **60**, 7649-7653.
2. H. Li, F. Zhou, T. L. D. Tam, Y. M. Lam, S. G. Mhaisalkar, H. Su and A. C. Grimsdale, *J. Mater. Chem. C,* 2013, **1**, 1745-1752.
3. J. Merz, M. Dietz, Y. Vonhausen, F. Wber, A. Friedrich, D. Sieh, I. Krummenacher, H. Braunschweig, M. Moos, M. Holzapfel, C. Lambert and T. B. Marder, *Chem. Eur. J*., 2020, **26**, 438-453.
4. Y. Hu, Y. Yuan, Y. L. Shi, J. D. Lin, Z. Q. Jiang and L. S. Liao, *J. Mater. Chem. C,* 2018, **6**, 1407-1412.
5. M. Wollenburg, D. Moock and F. Glorius, *Angew. Chem. Int. Ed*., 2019, **58**, 6549-6553.
6. G. M. Sheldrick, *Acta Cryst. A,* 2015, **71**, 3-8.
7. G. M. Sheldrick, *Acta Cryst. C,* 2015, **71**, 3-8.
8. O. V. Dolomanov, L. J. Bourhis, R. J. Gildea and J. A. K. Howard and H. Puschmann, *J. Appl. Cryst.,* 2009, **42**, 339-341.
9. (a) P. Thordarson, *Chem. Soc. Rev.* **2011**, 40, 1305-1323; (b) http://app.supramolecular.org/bindfit.
10. N. Mataga, *Bull. Chem. Soc. Jpn.,* 1963, **36**, 654-662.
11. N. Mataga, Y. Kaifu and M. Koizumi, *Bull. Chem. Soc. Jpn.,* 1956, **29**, 465-470.
12. M. Frisch, G. Trucks, H. Schlegel, G. Scuseria, M. Robb, J. Cheeseman, G. Scalmani, V. Barone, B. Mennucci, G. Petersson, H. Nakatsuji, M. Caricato, X. Li, H. Hratchian, A. Izmaylov, J. Bloino, G. Zheng, J. Sonnenberg, M. Hada, M. Ehara, K. Toyota, R. Fukuda, J. Hasegawa, M. Ishida, T. Nakajima, Y. Honda, O. Kitao, H. Nakai, T. Vreven, J. Montgomery, J. Peralta, F. Ogliaro, M. Bearpark, J. Heyd, E. Brothers, K. Kudin, V. Staroverov, R. Kobayashi, J. Normand, K. Raghavachari, A. Rendell, J. Burant, S. Iyengar, J. Tomasi, M. Cossi, N. Rega, J. Millam, M. Klene, J. Knox, J. Cross, V. Bakken, C. Adamo, J. Jaramillo, R. Gomperts, R. Stratmann, O. Yazyev, A. Austin, R. Cammi, C. Pomelli, J. Ochterski, R. Martin, K. Morokuma, V. Zakrzewski, G. Voth, P. Salvador, J. Dannenberg, S. Dapprich, A. Daniels, Farkas, J. Foresman, J. Ortiz, J. Cioslowski and D. Fox, Gaussian 09, Revision D.01. Gaussian 09, Revision D.01, Gaussian, Inc., Wallingford CT, 2013.
13. (a) A. D. Becke, *J. Chem. Phys.,* 1993, **98**, 5648-5652; (b) C. Lee, W. Yang and R. G. Parr, *Phys. Rev. B: Condens. Matter Mater. Phys.,* 1988, **37**, 785-789; (c) T. Yanai, D. Tew and N. Handy, *Chem. Phys. Lett.,* 2004, **393**, 51-57; (d) R. Ditchfield, J. W. Hehre and J. A. Pople, *J. Chem. Phys.,* 1971, **54**, 724-728; (e) W. J. Hehre, R. Ditchfield and J. A. Pople, *J. Chem. Phys.,* 1972, **56**, 2257-2261; (f) P. C. Hariharan, *J. A. Pople*, 1973, **28**, 213-222.
14. T. Lu and F. Chen, *J. Comput. Chem.,* 2012, **33**, 580-592.
15. J. Yuan, Y. Yuan, X. Tian, Y. Liu and J. Sun, *J. Phys. Chem. C,* 2017, **121**, 8091-8108.
16. X. Tang, L. S. Cui, H. C. Li, A. J. Gillett, F. Auras, Y. K. Qu, C. Zhong, S. T. E. Jones, Z. Q. Jiang, R. H. Friend and L.S. Liao, *Nat. Mater.,* 2020, **19**, 1332-1338.
17. H. Isobe, K. Nakamura, S. Hitosugi, S. Sato, H. Tokoyama, H. Yamakado, K. Ohnobd and H. Kono, *Chem. Sci.,* 2015, **6**, 2746-2753.
18. I. H. M. Van Stokkum, D. S. Larsen and R. Van Grondelle, Biochim. *Biophys. Acta. Bioenerg.*, 2004, *1657*, 82-104.
19. K. M. Mullen and I. H. M. Van Stokkum, *J. Stat. Soft.,* 2007, **18**, 1-46.
20. Z. Kuang, G. He, H. Song, X. Wang, Z. Hu, H. Sun, Y. Wan, Q. Guo and A. Xia, *J. Phys. Chem. C*, 2018, **122**, 3727-3737.
